# Reduced ING1 levels in breast cancer promotes metastasis

**DOI:** 10.18632/oncotarget.1988

**Published:** 2014-05-19

**Authors:** Satbir Thakur, Arvind K. Singla, Jie Chen, Uyen Tran, Yang Yang, Carolina Salazar, Anthony Magliocco, Alexander Klimowicz, Frank R. Jirik, Karl Riabowol

**Affiliations:** ^1^ Department of Biochemistry and Molecular Biology, University of Calgary, Calgary, Alberta, CANADA; ^2^ Department of Oncology, University of Calgary, Calgary, Alberta, CANADA; ^3^ Moffitt Cancer Center, Tampa, Florida USA; ^4^ Functional Tissue Imaging Unit, Translational Research Laboratory, Tom Baker Cancer Center, Calgary, Alberta, CANADA; ^5^ Present Address: Boehringer Ingelheim Pharmaceuticals, Inc., Ridgefield, CT, USA

**Keywords:** ING1, epigenetics, breast cancer, metastasis, survival

## Abstract

INhibitor of Growth 1 (ING1) expression is repressed in breast carcinomas, but its role in breast cancer development and metastasis is unknown. ING1 levels were quantified in >500 patient samples using automated quantitative fluorescence immunohistochemistry, and data were analysed for correlations to patient outcome. Effects of altering ING levels were examined in microarrays and metastasis assays *in vitro*, and in a mouse metastasis model *in vivo*. ING1 levels were lower in tumors compared to adjacent normal breast tissue and correlated with tumor size (p=0.019) and distant recurrence (p=0.001) in ER- or Her2+ patients. In these patients ING1 predicted disease-specific and distant metastasis-free survival. Transcriptome analysis showed that the pathway most affected by ING1 was breast cancer (p = 0.0008). Decreasing levels of ING1 increased, and increasing levels decreased, migration and invasion of MDA-MB231 cells *in vitro*. ING1 overexpression also blocked cancer cell metastasis *in vivo* and eliminated tumor-induced mortality in mouse models. Our data show that ING1 protein levels are downregulated in breast cancer and for the first time, we show that altering their levels regulates metastasis *in vitro* and *in vivo*, which indicates that ING1 may have a therapeutic role for inhibiting metastasis of breast cancer.

## Introduction

Breast cancer, the most common type of cancer diagnosed in women worldwide is heterogeneous in molecular profile and pathology, making it difficult to diagnose and treat. Triple-negative breast cancers, in particular, are difficult to treat because lack of epidermal growth factor receptor 2 (HER2), estrogen receptor (ER) and progesterone receptor (PR) expression makes them resistant to hormone or HER2 targeted therapies.

ING1 was initially identified in a screen to identify genes repressed in breast cancer [1] and it was subsequently seen to be downregulated in both familial and sporadic breast cancers [2, 3]. Based upon sequence homology to ING1, INGs 2-5 were identified and they all serve as stoichiometric components of histone deacetylase (HDAC; ING1 and ING2) and histone acetyl transferase (HAT; ING3-5) complexes [4], altering chromatin structure [5, 6] and affecting transcription [7]. Since their stoichiometry is important for proper HAT and HDAC function, alteration of their levels affects the epigenome and cell viability [6-16]. All ING proteins contain plant homeo domains (PHDs) that avidly interact with trimethylated lysine 4 of histone H3 (H3K4Me3), making them epigenetic histone readers [8, 9] that target HAT and HDAC complexes to sites of active gene transcription [6, 10]. Apart from being histone readers, ING proteins also affect DNA demethylation [11].

Metastasis and invasion are thought to be responsible for ~90% of cancer-associated mortality. For metastasis to occur, cancer cells attain a specific genotype and epigenotype, which allows them to disseminate from the primary tumor mass and survive and proliferate at secondary sites. Epigenetic silencing of the E-cadherin gene *CDH1* through DNA methylation [12] and recruitment of HDACs [13] to its promoter have been previously linked to metastasis and tumor progression. Also, repressive chromatin modifications such as H3K27Me3 and DNA methylation restrict the expression of metastasis associated genes in various cancers [14] and breast cancers have a distinct epigenomic DNA methylation profile, which affects their metastatic potential [15].

ING proteins inhibit the growth of cancer cells *in vitro* and *in vivo* when overexpressed from adenoviral vectors [16-18]. They also enhance chemosensitivity in combination with etoposide, doxorubicin [19] and epigenetic drugs like panobinostat and 5-azacytidine [20]. Due to their tumor suppressive nature and stabilization of the pro-apoptotic p53 protein [21], expression of ING proteins and cancer specific survival has previously been studied. Loss of ING proteins was generally found to correlate with cancer progression [22-25] and inhibiting the function of ING1 in chromatin modification by cytoplasmic localization, also positively correlated with tumor progression in head and neck cancers [22]. ING4 inhibited invasion and migration in a melanoma cell model *in vitro* [23] while ING1 and ING4 were reported to inhibit angiogenesis in glioblastoma [26, 27] and to negatively correlate with microvessel density in ovarian cancers [28]. *ING1* was also reported to be a target of miR-622, which inhibits cell migration and invasion [29].

In this study, we evaluate the prognostic significance of ING1 in breast cancer with particular emphasis on distant metastasis-free survival. Results obtained were independently validated *in vitro* using cell based assays and an *in vivo* mouse experimental metastasis model, which establish that a strong negative correlation exists between metastasis in breast cancer patients and ING1 expression. These data indicate that ING functions in regulating cell motility and by affecting the invasive properties of cells that underlie metastasis.

## Results

### ING1 regulates genes related to breast cancer

Prior studies showed that ING1 overexpression selectively killed breast cancer cells *in vitro* and in a mouse mammary model [20] while reduced ING1 expression was seen in >40% of primary breast tumors [20]. To examine how ING1 might limit cancer cell growth and survival, we identified genes that were regulated by ING1 using a Nimblegen microarray-based platform. As shown in supplementary Fig 1A, the analysis identified 844 genes that were reproducibly induced, and 1,500 that were repressed at least two-fold in response to ING1b overexpression. As shown in the complete list of regulated genes in supplementary Table 1, 14-3-3 sigma (SFN), a gene frequently repressed in breast cancer [30] was the gene most highly induced by ING1, while a PDGF receptor gene (PDGFRA) was most highly repressed. Pathway analysis of ING1-repressed genes showed that breast cancer had the strongest association (p=0.0008; kappa similarity score=1.0 where 0.75-1.0=very high; 0.5-0.75=high, 0.25-0.5=moderate and below 0.25=low) followed by colorectal cancer (supplementary Fig1B, 1C), while genes transcriptionally activated had less clear links to cancer pathways (data not shown).

### ING1 levels are reduced in breast cancer cells

Our study using the retrospective Calgary Tamoxifen Breast Cancer Cohort, included 532 patients diagnosed with invasive breast cancer, treated at the Tom Baker Cancer Centre (TBCC) between 1985 and 2000. Selection criteria are outlined in Materials and Methods and clinico-pathologic characteristics are shown in supplementary Table 2. Median follow-up time for the cohort was 82.1 months. Mean age was 66 years and the majority of patients (85%; n=451) were postmenopausal women when age was dichotomized around the median age of menopause in Canada (52 years). Patients were distributed between stages [31], with 44% (n=233) stage I, 31% stage II (n=163), 8.0% stage III (n=40), and 1% stage IV (n=7). 79% of patients had a low-grade tumor (n=419, tumor grade 1 or 2), 51% (n=271) had a tumor size of less than 2cm, and 64.0% (n=342) were lymph node negative. A minority of patients had disease progression within 5 years of diagnosis (18%, n=95), and the majority of these patients also developed distant metastatic disease within this timeframe (14%, n=74). ER, PR, and Her2 status were not systematically performed at the time of diagnosis for many of the patients in this cohort; retrospective IHC-based analysis of the TMAs was performed to determine the status of each of these biomarkers.

**Figure 1:**
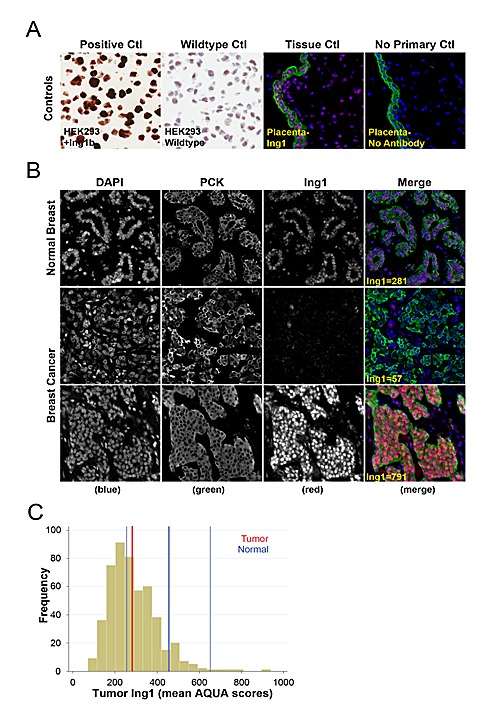
Immunohistochemical staining and quantitation of ING1 using the HistoRx AQUA platform **(A)** Representative images showing specificity of the ING1 monoclonal antibody in HEK293 cells and HEK293 cells overexpressing ING1 (left panels) and in placenta treated with or without the ING1 antibody (right panels). **(B)** Representative examples of quantitative fluorescent IHC images for ING1 expression in normal breast tissue (top row of panels) and breast cancer tissue (two bottom rows of panels). AQUA scores represent the expression level of ING1 within the pan-cytokeratin defined epithelial/tumour compartment. DAPI-stained nuclei are depicted in blue, pan-cytokeratin-stained epithelial/tumour cells are depicted in green, and ING1 protein expression is depicted in red. **(C)** Histogram distribution representing ING1 expression within breast cancer patient samples. The solid blue line represents median ING1 expression in normal breast tissue, the broken blue lines represent 95% CI from median ING1 expression in normal breast tissue, and the solid red line represents median ING1 expression in breast cancer patient samples.

ING1 protein level was measured using quantitative fluorescence immunohistochemistry on the HistoRx AQUA® platform [32]. The specificity of the ING1 monoclonal antibody used for fluorescence IHC was assessed using control transfected 293 cells and ING1 overexpressing 293 cells [33]. Endogenous ING1 expression was weak and nuclear in the control 293 cells, whereas overexpressed ING1 was strong and present in both the nuclear and cytoplasmic compartments (Fig 1A, left panels). The specificity of the ING1 fluorescence IHC assay was confirmed by comparing Cy3 signal detection in placenta treated with or without the ING1 antibody (Fig 1A, right panels). ING1 staining in normal breast tissue was weak and predominantly nuclear. ING1 levels were similar in ductal epithelium, myoepithelium, and stromal cells (Fig 1B, top panels). In breast cancers with low ING levels, staining was weaker than in surrounding non-malignant stromal cells (Fig 1B, mid panels). In tumors expressing high levels of ING1, ING1 staining was strongly nuclear with clearly detectable cytoplasmic protein compared with the weaker nuclear and diffuse cytoplasmic ING1 staining in surrounding non-malignant stromal cells (Fig 1B, lower panels).

To determine whether ING1 expression was altered in breast cancer cells compared to normal breast epithelial cells we compared ING1 expression within our Tamoxifen-treated breast cancer cohort to a 95% confidence interval (C.I.) around the median results obtained from normal breast epithelium (Fig 1C). Median tumor ING1 expression was 267 (red line) and fell at the lower end of the normal breast 95% C.I. (ING1=254-660, thin blue lines) indicating that ING1 expression tends to be lost in breast cancer cells as compared to the normal epithelium from which they are derived.

### Prognostic value of ING1 protein expression

**Figure 2:**
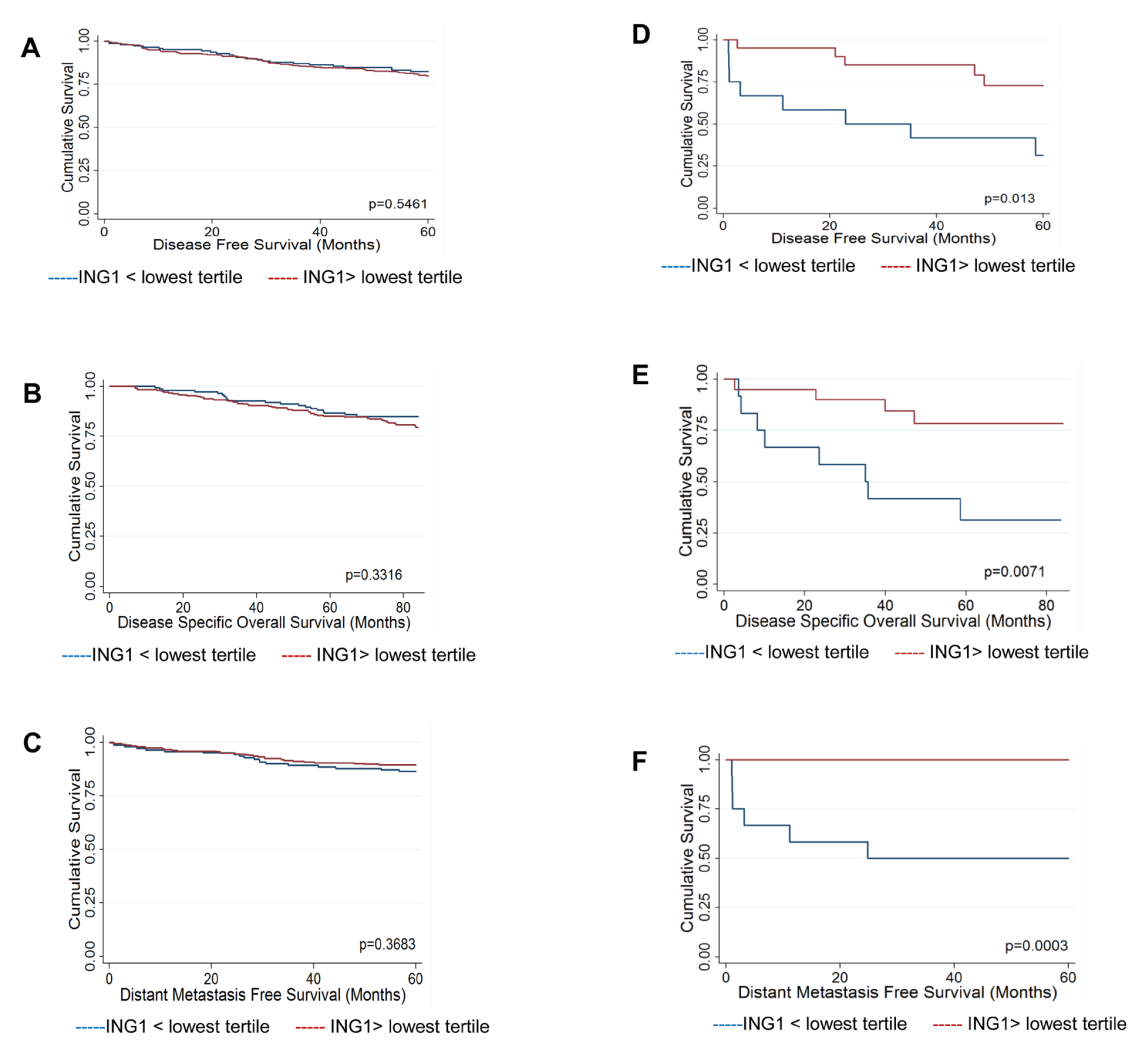
Kaplan-Meier survival analysis. (A-C) Survival of patients in the ER+/Her2- group Kaplan-Meier survival curves for **(A)** disease free survival **(B)** disease specific overall survival and **(C)** distant metastasis free survival. No difference was noted between breast cancer patients with below lowest tertile or above lowest tertile of ING1levels. **(D-F)** Survival of patients in the ER- or Her2+ group. Kaplan-Meier survival curves for **(D)** disease free survival **(E)** disease specific overall survival and **(F)** distant metastasis free survival. ING1 levels positively correlate with the three categories of survival in ER- or Her2+ breast cancer patients.

As the Calgary Tamoxifen Breast Cancer Cohort is not defined by a particular subtype of breast cancer and the different subtypes are known to have distinct biology, we classified patients for which there was corresponding ING1 expression data into luminal breast cancer (ER positive and Her2 negative, n=430) and non-luminal breast cancer (ER negative or Her2 positive, n=32) groups for analysis. Breast cancer patients were further dichotomized at the lowest tertile of ING1 expression (ING1< 226), as assessed in all patients for which there was an ING1 score (n=501), to identify low and high ING1 expressing tumors within the luminal and non-luminal subtypes. This cutpoint was selected as, unlike median ING1 expression (ING1=267), the lowest tertile falls below the 95% confidence interval for ING1 expression in normal breast epithelium (ING1=254-660), identifying a population of tumors that have substantial loss of ING1 expression compared to normal tissue. Loss of ING1 did not correlate with any clinico-pathological variables in the luminal group, whereas low ING1 expression correlated with tumor size greater than 2cm (p=0.019) as well as recurrence (p=0.030) and distant recurrence (p=0.001) in the non-luminal group (supplementary Table 2). No differences in survival outcomes were seen by Kaplan Meier analysis in the luminal group dichotomized by ING1 expression (Fig 2A-C). However, in the non-luminal group low ING1 levels correlated with survival outcomes, including: disease free survival (Fig 2D, logrank p=0.013), disease specific overall survival (Fig 2E, logrank p=0.0071), and distant metastasis free survival (Fig 2F, logrank p=0.0003).

### ING1 protein levels regulate migration and invasion of MDA-MB231 cells

**Figure 3:**
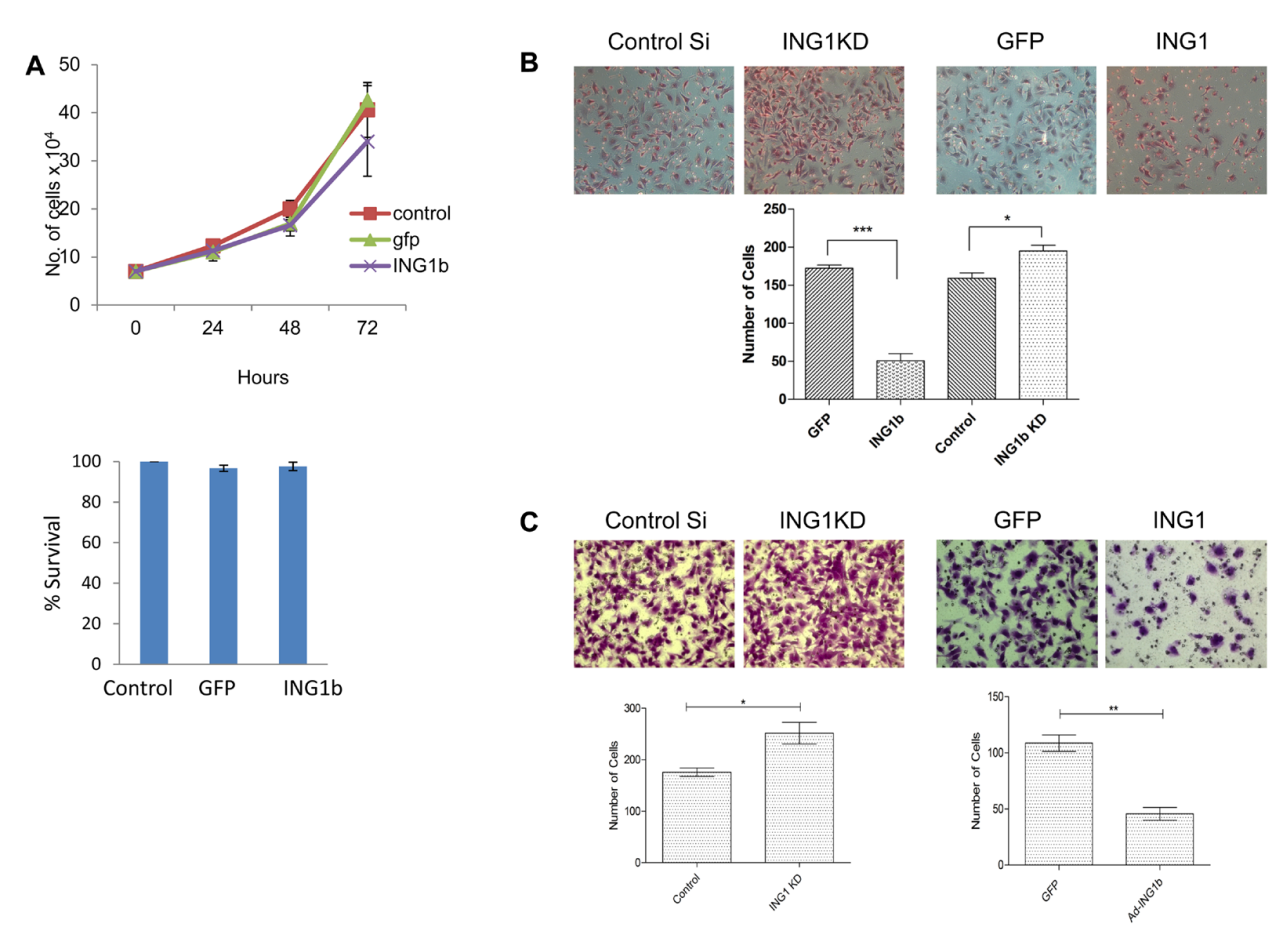
ING1 protein levels regulate migration and invasion of MDA-MB231 cells *in vitro* **(A)** Change in number/survival of MDA-MB231 cells upon infection with adenovirus expressing GFP or GFP + ING1 determined by cell count (top) and MTT assay (bottom). **(B)** Representative images and quantification from transwell migration assays (n=3) and **(C)** matrigel invasion assays upon ING1 overexpression or knockdown in MDA-MB231 cells (n=3; *p<0.05, ***p<0.001).

We next evaluated the ability of ING1 to regulate migratory and invasive behavior of the MDA-MB231 triple negative breast cancer cell line. Ectopic expression of ING1 did not appear to block growth or induce cell death in MDA-MB231 cells as reported previously for INGs in other cell types [8, 9] (Fig 3A). Consistent with this, and with ING1 inhibiting migratory behavior, initial scratch tests suggested that expression of ING1 by infection with adenovirus inhibited the ability of MDA-MB231 cells to migrate to fill in wounds in cell monolayers (supplementary Fig 2). We then checked the migratory properties of these cells upon ING1 overexpression and knockdown using a transwell migration assay. ING1 inhibited migration to the lower chamber by ~3.5 fold (Fig 3B) whereas ING1 knockdown increased the number of migratory cells by 1.3 fold, compared to controls. These reciprocal results corroborate results from the scratch tests and are consistent with ING1 negatively regulating cell migration.

Metastatic cancer cells actively penetrate the basement membrane to migrate and form tumors at distant sites. Given the link between low ING1 levels and lymph node involvement, we asked if ING1 could play a role in breast cancer cell invasion. As shown in Fig 3C, ING1 overexpression reduced the ability of MDA-MB231 cells to invade through the matrigel membrane. Similarly, ING1 knockdown had a reciprocal effect as cells with reduced levels of ING1 showed increased invasive capacity compared to control cells. Similar results were obtained when ING1 levels were modulated in normal mesenchymal human foreskin fibroblasts (HS68) as ING1 also regulated the invasive capacity of these cells *in vitro* (supplementary Fig 3).

### Mechanism of metastasis inhibition by ING1

**Figure 4:**
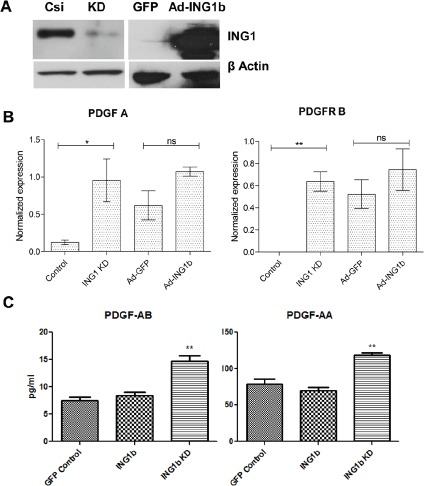
ING1 affects the PDGF/PDGFR pathway **(A)** Representative western blot image showing levels of ING1 protein upon knockdown using siRNA and overexpression using an adenoviral construct encoding GFP and ING1 under separate promoters. **(B)** Expression of PDGF-A and PDGFR-B in MDA-MB231 cells upon ING1 overexpression or knockdown as determined by Q-RT PCR (n=3; *p<0.05, **p<0.001). **(C)** Amount of PDGF AB/AA protein present in the media supernatant of ING1 overexpressing or knockdown MDA-MB231 cells determined by ELISA (**p<0.001).

To identify mechanisms potentially responsible for altered invasive ability of MDA-MB231 cells upon changing ING1 protein levels, we quantified expression of various EMT related genes that were also regulated by ING1 (Yang *et al*, in preparation). The PDGF/PDGFR pathway was of particular interest because of its established role in promoting metastasis in various types of cancers [34, 35] and our initial microarray results. To test the effects of ING1 on PDGF signaling, ING1 was overexpressed or knocked down to levels seen in Fig 4A. Both PDGF A and PDGFR B mRNA were upregulated by knockdown of ING1 as assessed by quantitative real time PCR (Fig 4B). It was also observed that PDGF-AA/AB protein levels were increased in the conditioned media of the ING1 knockdown cells as compared to control cells (Fig 4C). These data indicate that ING1 knockdown activates the PDGF/PDGFR pathway, which increases motility and invasiveness [35,36].

### ING1b overexpression inhibits the development of metastases and improves survival

**Figure 5:**
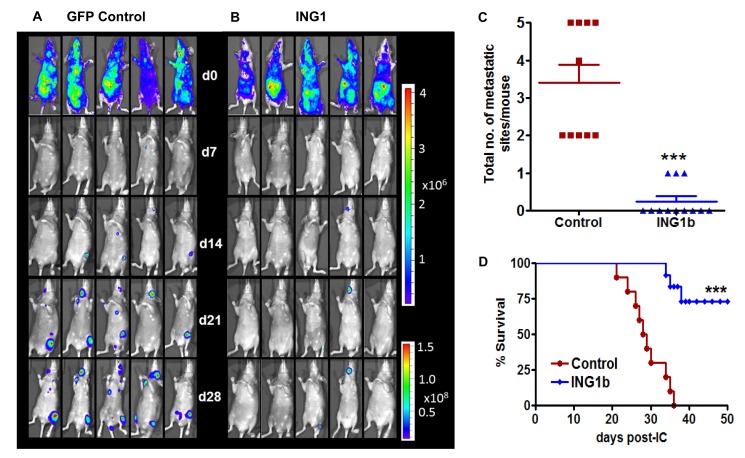
ING1b overexpression inhibits metastasis *in vivo* and improves survival Representative ventral bioluminescence images (BLI) taken on days 0, 7, 14, 21 and 28 from mice injected with control MDA-MB-231-luc2 cells **(A)** or ING1b overexpressing MDA-MB-231-luc2 cells **(B)**. Subsets of mice were sacrificed between days 28-35 due to ethics guidelines for permissible tumour burden. **(C)** Scatter dot plot showing decrease in total number of metastatic sites per mouse in ING1b overexpressing group (n=12) compared to controls (n=10; p=0.0001). **(D)** Kaplan–Meier survival curve showing increase in overall survival for ING1b overexpressing group of mice (n=12) compared to controls (n=10, log-rank test ***p<0.001).

Given the *in vitro* results, ING1b overexpression in highly metastatic MDA-MB231-luc2 cells might also reduce the development and progression of metastasis *in vivo*. To test this, MDA-MB231-luc2 cells were infected with adeno-ING1b GFP or adeno-GFP for 24hrs. The cells were then injected into the arterial circulation of NIH-III (*nu/nu; beige/beige*) mice. To monitor location and growth of metastatic tumors, bioluminescence imaging (BLI) was carried out at 7, 14, 21 and 28 days post-tumor cell inoculation in both control and ING1b overexpressing groups (Fig 5A, 5B). While metastatic burden as estimated by bioluminescence increased in the control group of mice from day 7 (1.3 × 10^6^) to 28 (2.2 × 10^9^) by ~1,700-fold, the ING1b mice showed dramatically reduced bioluminescence levels with bioluminescence increasing ~24-fold (3 × 10^6^ to 7.3 × 10^7^) in the same time frame for an overall ~70-fold reduction (p<0.001). ING1b overexpressing mice also had fewer metastatic sites when compared to controls (Fig 5C; p=0.0001). Notably, only 3 out of 12 mice in the ING1b overexpressing group developed a single metastatic site per mouse whereas 2-5 metastatic sites per mouse were found in the control group. ING1b overexpressing mice also showed enhanced survival compared to the control mice (Figure 5D; p<0.0001).

### ING1b overexpression completely blocks the development of knee metastases

**Figure 6:**
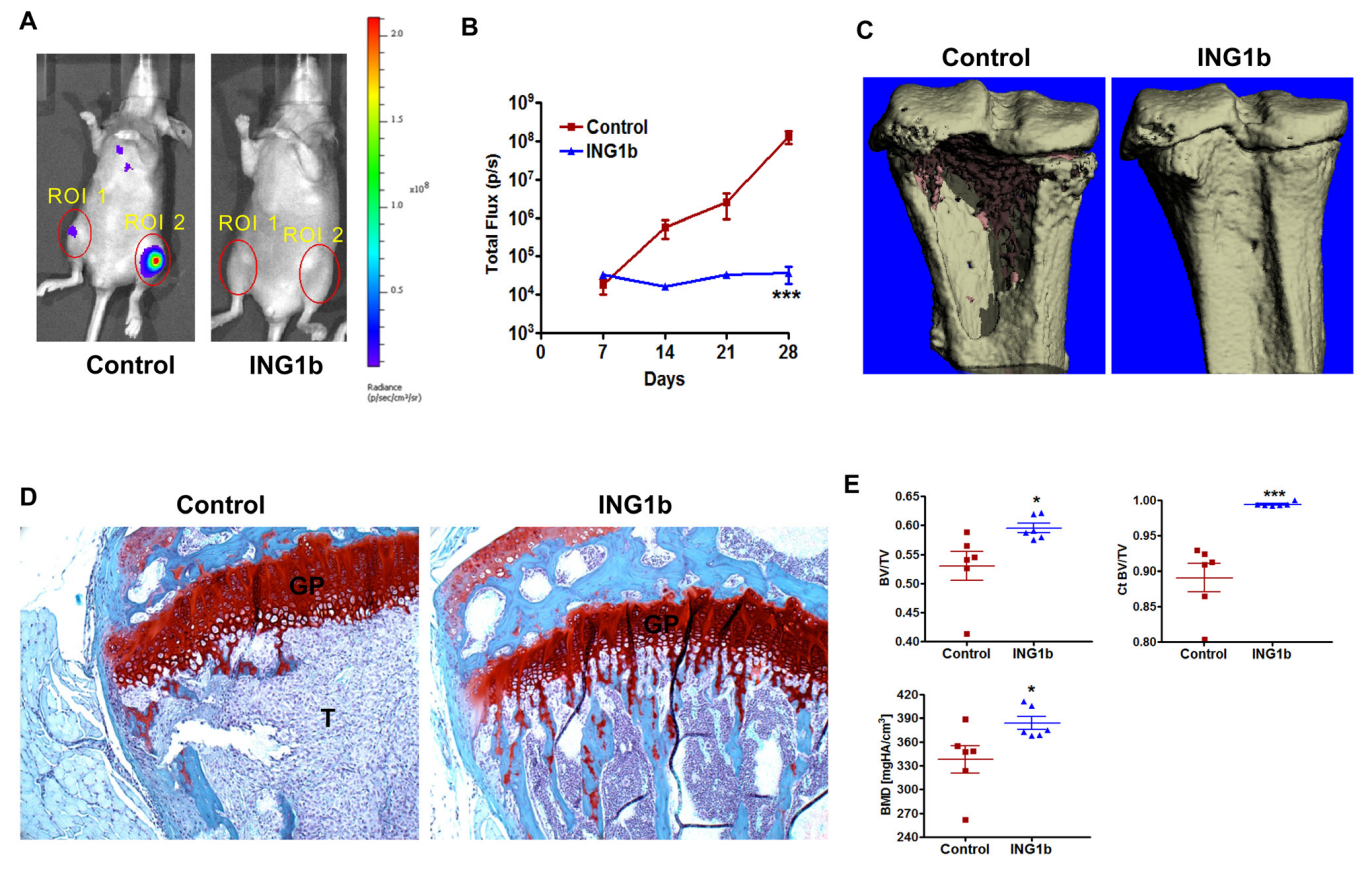
ING1b completely blocks the initiation and progression of knee metastasis **(A)** Representative ventral BLI are shown from control and ING1b overexpressing mice to visualize differences in knee metastasis between these groups. Combined regions of interest (ROI 1+ ROI 2) are shown with red circles over the knees. **(B)** A line graph showing combined knee bone metastatic growth comparison between control and the ING1b overexpressing group showing complete inhibition of knee metastasis progression in the ING1b group of mice (***p<0.001). **(C)** Representative μCT images from the proximal tibia of control and ING1b overexpressing mice. Extensive tumor-induced cortical bone loss is seen in the control group of mice compared to ING1b overexpressing mice where no bone loss was observed. **(D)** Histology of knee bone stained with trichrome. Control bone displays a large tumor (T=tumor area) whereas bone from an ING1b overexpressing mouse shows no tumor. GP-growth plate. **(E)** μCT bone parameter analysis between control and ING1b overexpressing groups of mice showing data on bone volume/total volume (BV/TV), Cortical bone volume/total volume (Ct BV/TV) and bone mineral density (BMD) (n=6 per group; * p<0.05; *** p<0.001).

MDA-MB231-luc2 cells used in this study have a strong propensity to generate knee osteolytic metastases [36]. BLI of these mice show that unilateral or bilateral knee metastases develop within 2-3 weeks (Fig 5A). Representative knee regions of interest (ROI) from control and ING1b overexpressing mice are shown in Fig 6A. BLI quantification of knee ROI revealed that there was no increase in photon emission in the ING1b overexpressing mice for the entire duration of the experiment, which sharply differed from control mice (Fig 6B; p<0.001). 3D μCT imaging of knees from the control group confirmed extensive bone osteolytic damage from metastases compared to the ING1b overexpressing group of animals (Fig 6C). Similarly, trichrome staining of control knee bones showed the presence of large tumors, while no tumors were apparent in the knee bones of ING1b mice (Fig 6D). ING1b overexpression resulted in the complete inhibition of tumor-induced bone osteolysis and a consistent preservation of bone integrity as assessed by bone parameters, bone volume divided by total volume (BV/TV), cortical bone volume divided by total volume (Ct BV/TV) and bone mineral density (BMD) (Fig 6E).

## Discussion

In this study we show that altering the levels of ING1 affects the expression of genes known to be altered in breast cancer, consistent with ING1 being initially identified as a gene repressed in breast cancer cell lines [1] that can regulate gene expression [7]. AQUA analysis of samples from 532 breast cancer patients extends previous studies that have used limited numbers of samples and were lacking internal controls that reduction of ING1 levels frequently occurs in primary breast. Here we also show that higher levels of ING1 correlate strongly with disease free survival (logrank p=0.013), disease specific overall survival (logrank p=0.0071), and distant metastasis free survival (logrank p=0.0003) in non-luminal, but not in luminal breast cancers. These data suggest that ING1 plays a role in curbing metastasis, and indeed, knockdown of ING1 promoted, and overexpression of ING1 inhibited cell migration and invasion by several experimental measures. These included scratch and transwell migration assays and a matrigel membrane invasion assay. By examination of genes that were found previously to be associated with the epithelial-mesenchymal transition [37] and that were also found to be regulated by ING1 (Yang *et al*., in preparation), we identified platelet-derived growth factor A (PDGF A) and the platelet-derived growth factor receptor B (PDGFR B) as genes that were regulated by ING1 in a similar manner in the MDA-MB231 human breast adenocarcinoma cell line that is used widely for invasion assays. Knockdown of ING1 by ~90% caused very robust increases in both PDGF-A and PDGFR-B, as might be expected if the usual function of ING1 was to repress these genes whose products are associated with invasion and metastasis [35, 38]. Although a logical expectation might be to see a decrease in these gene transcripts in response to ING1 overexpression, we did not find this to be the case. Overexpression of ING1 caused no significant changes in transcript levels of either of these genes, suggesting that overexpression of ING1 did not increase the activity of the Sin3A HDAC complex that contains ING1 as a stoichiometric member [4]. Both ING1 and the closely related ING2 protein are believed to exert cellular effects primarily by altering gene expression through epigenetic mechanisms, specifically binding to, and targeting the HDAC complex to the H3K4Me3 mark [8, 9]. If anything, some increase in gene expression might be expected if overexpression impaired the function of the Sin3A complex as previously proposed, due to altering stoichiometry [41]. Although not to statistically significant levels, such an increase in response to ING1 overexpression was indeed noted in this study (Fig 4B).

ING1 overexpression also significantly reduced the number of metastatic tumors in the experimental mouse model as evident from BLI (Fig 5A). Animals with MDA-MB231 cells infected with ING1 + GFP adenovirus had significantly fewer metastatic growth sites than mice injected with MDA-MB231 cells infected with control GFP adenovirus and in fact only three mice in the group (n=12) had any signal detected at all after 28 days (Fig 5C). Experimental mice also showed increased survival compared to animals with GFP only expressing cells in this model developed for examining skeletal metastasis of MDA-MB231 cells specifically to the knee bone [36]. Our findings demonstrated that ING1 overexpression completely abrogated the ability of MDA-MB231 cells to produce metastatic growths in the knee. Thus, mice with ING1 overexpressing breast cancer cells had no tumor burden in the knees, compared to controls which had a clear burden as determined by the BLI, μCT imaging and bone histology studies (Fig 6).

All of the ING proteins affect histone acetylation in yeast through human cells [6, 10, 39] and ING2 serves as the major target of the HDAC inhibitor SAHA [40]. Several HDAC inhibitors are in different phases of clinical trials where they have shown promising results for treating breast cancer as part of combination therapies [41, 42]. Since ING1 and ING2 are very closely related evolutionarily [43] and functionally [4], it is likely that both target the Sin3A HDAC complex to chromatin locales containing relatively higher density of the H3K4Me3 chromatin mark. This mechanism is consistent with recent observations that the epigenetic targeted drugs 5-azacytidine and the LBH589 HDAC inhibitor can act additively, or in some cases synergistically with ING1 in killing cells in breast cancer cell and animal models [20]. Data generated in this study provide mechanistic insight into why breast cancer cells may be selectively sensitive to HDAC inhibitors compared to normal breast epithelium; down-regulation of ING1 would already reduce the ability of cancer cells to accurately target the Sin3A complex and so treatment with HDAC inhibitors such as SAHA that selectively target ING2 and/or ING1 would be expected to have greater effects upon the epigenomes of cancerous versus normal epithelial cells. Since HDACs are also components of estrogen receptor complexes and HDAC inhibitors have been reported to be able to restore sensitivity of breast cancer cells to tamoxifen [44], this may explain, in part, why ING1 levels are able to predict survival in non-luminal forms of breast cancer as we report here for the first time.

## Materials and Methods

### Patient Cohort

The Calgary Tamoxifen Cohort contains demographic, clinical and pathology data for 819 breast cancer patients diagnosed between 1985 and 2000 at the Tom Baker Cancer Centre in Calgary, Canada. Inclusion criteria were a confirmed diagnosis of invasive breast carcinoma, primary surgical intervention, and adjuvant tamoxifen therapy (20 mg p.o./day). Exclusion criteria were the absence of available surgical formalin-fixed paraffin-embedded (FFPE) tissue, prior cancer diagnosis (except non-melanoma skin cancer), or treatment with primary or adjuvant chemotherapy. For more details please see supplementary material. Supplementary table 2 shows clinical-pathological characteristics of the subjects.

### Fluorescence Immunohistochemistry

After tissue microarray construction, 4 m thick sections were cut from the TMA block and deparaffinized in xylene, rinsed in ethanol, and rehydrated. Heat-induced epitope retrieval was performed by heating slides to 121°C in a citrate-based buffer (pH 6.0) target retrieval solution (Dako, Mississauga, ON, Canada) for 6 minutes, in a decloaking chamber (Biocare Medical, Concord, CA, USA). Slides were stained using a Dako Autostainer. Endogenous peroxidase activity was quenched with a 10 minute incubation of peroxidase block (Dako) followed by a 15 minute protein block (Signal Stain, Cell Signaling, Danvers, MA, USA) to eliminate non-specific antibody binding. Slides were washed with TBST wash buffer (Dako) and then incubated at room temperature for 60 minutes with Signal Stain protein block (Cell Signaling) containing a 1:500 dilution of mouse anti-ING1 mAb, clone CAb5 (SACRI Antibody Facility, University of Calgary, Calgary, AB, Canada). Additional antibodies including anti-pan-cytokeratin guinea pig monoclonal (Acris, San Diego, CA, USA), anti-vimentin rabbit mAb, clone EPR3776 (Epitomics, Burlingame, CA, USA) and Alexa-488 conjugated goat anti-guinea pig antibody (Invitrogen, Burlington, ON, Canada) were used as suggested by suppliers.

### Automated image acquisition and analysis

Automated image acquisition was performed using an Aperio Scanscope FL (Aperio Inc., Vista, CA, USA). High-resolution slide images were acquired using the Scanscope FL 8/10-bit monochrome TDI line-image capture camera using filters specific for DAPI to define the nuclear compartment, FITC to define cytokeratin positive cells and the tumor cytosolic compartment, Cy3 to define the target biomarker ING1, and Cy5 to define vimentin positive non-malignant stromal cells.

Images were analysed using the AQUAnalysis® program, version 2.3.4.1 as previously described [32]. Briefly, a tumor-specific mask was generated to distinguish breast cancer cells from surrounding stromal tissue by thresholding the pan-cytokeratin images. Threshold levels were verified and adjusted by spot-checking a small sample of images to determine an optimal threshold value. All images were then processed using this optimal threshold value and all subsequent image manipulations involved only image information from the masked area. Images were validated according to the following: 1) >10% of the tissue area is pan-cytokeratin positive, 2) >50% of the image was usable (i.e. not compromised due to overlapping or out of focus tissue). Unusable areas within each image were manually cropped and excluded from the final analysis.

### Assessment of ING1 Expression

The average intensity of target ING1 signal in the tumor mask was tabulated and used to generate tumor specific AQUA scores, which reflect the average signal intensity per tumor area. The ING1 expression score was defined as the mean ING1 malignant cell-specific AQUA score from triplicate cores for each patient sample. Patients were dichotomized at the lowest tertile of ING1 expression within the entire cohort, to define Low ING1 and High ING1 expression categories.

### Cell culture and transfection

MDA-MB231 cells from ATCC (HTB-26) and MDA-MB231 cells stably expressing an EGFP-luc2 fusion protein [36] were cultured in H-DMEM (Lonza) supplemented with 10% FBS, 0.1 mg/ml streptomycin and 100U/ml penicillin were maintained as per ATCC guidelines. Cells were confirmed to be free of pathogenic murine viruses and mycoplasma by PCR testing at Charles River Laboratories. siRNA transfections were done using Lipofectamine 2000 (InVitrogen) according to the manufacturer's guidelines. ING1 siRNA smartpool and scrambled siRNA were from Thermoscientific.

### Cell Motility and Invasion Assays

For experiments involving ING1 knockdown, cells were transfected with an ING1 siRNA pool or scrambled siRNA and incubated for 48 hours. For experiments involving ING1 overexpression, cells were infected with Ad-ING1b + GFP or Ad-GFP at an MOI of 15. After 24 hours, cells were trypsinized and 2.5x10^4^ cells were added to 8μm pore size inserts (BD Biosciences) to perform transwell migration assay as per manufacturer's instructions. For invasion assays, cells were treated as described, but 3.5x10^4^ were placed in 8μm pore size Matrigel coated invasion chamber inserts (BD BioCoat) and incubated for 24 hours.

### Multiplex Assay for Cytokine and Chemokine screening

Media from transfected/infected MDA-MB231 cells were collected and screened for released cytokines and chemokines using an ELISA based assay (Eve Technologies, Calgary Alberta).

### NIH-III (*nu/nu; beige/beige*) mice

NIH-III (*nu/nu; beige/beige*) female mice (5-week old) were purchased from Charles River Laboratories (St. Constant, QC). Mice were housed in a biohazard facility at the Animal Resource Centre (ARC) of the University of Calgary. Housing and treatments were in compliance with Canadian Council of Animal Care guidelines and ethical approval from the University of Calgary Animal Care Committee.

### Breast cancer experimental metastasis model

To generate metastases, 6-week old female (16-18 grams) NIH-III mice were anesthetized by intraperitoneal (i.p) injection of ketamine (100mg/kg) and xylazine (10mg/kg), and then given 150 mg/kg D-luciferin (Gold Biotechnology, St. Louis, MO). Mice were then inoculated with 2x10^5^ MDA-MB231-luc2 cells suspended in 100uL of PBS, by intra-cardiac (i.c.) injection in the left ventricle of the heart. Metastases were monitored by bioluminescence imaging on day 7, 10, 14, 17, 21, 28 and 35 post-injection. To visualize and to quantify metastatic growth, bioluminescence imaging (Xenogen/Caliper) was used, and anatomical sites of soft tissue metastasis were confirmed by *ex vivo* bioluminescence of organs at necropsy. Image analysis was performed using the Living Image® 4.1 software from Caliper Life Sciences. The bioluminescence signal intensity was quantified with the Living Image 4.2 software, as total photon flux (photons/s) in a uniform region of interest (ROI) or flux from the whole body. For *ex vivo* imaging, organs were placed in 24-well cell culture plates along with 200 uL of D-luciferin (15 mg/mL) and imaged for 2 minutes.

### Micro-computed tomography (μCT)

Knee bone loss induced by bone metastases was assessed by μCT. Hind limbs were dissected and cleaned of muscle tissue before fixation in 4% PFA for 7 days and scanned in a μCT scanner (vivaCT 40, Scanco Medical, Switzerland). For more details refer to supplementary section.

### Histology

Fresh hind limbs with bone metastasis tumors from control and ING1b overexpressed group of mice were fixed in 4% PFA and decalcified for 2 weeks in 14% EDTA at pH 8.0 with changes every 24 hrs. Tissues were embedded in paraffin (Paraplast-Plus, -X-tra (50), McCormick Scientific), and 4-8 μm sections were cut. A tri-chrome stain was performed for histological examination of knee sections.

### Statistical Analysis

Statistical analyses were performed using Stata 12 (StataCorp LP). Histogram distributions were used to compare the distributions of tumor ING1 expression scores to those from normal breast epithelium (n=7). For survival analysis, the events under study were disease free survival (DFS), defined as the time of diagnosis to recurrence, metastatic disease, or death from breast cancer; distant metastasis free survival (DMFS), defined as the time of diagnosis to recurrent metastatic disease; and disease specific overall survival (DSOS), defined as the time of diagnosis to death from breast cancer. Patients were censored at the time a patient died from another cause, or the follow-up period ended. Kaplan Meier survival analysis was performed to estimate the probability of 5-year DFS, 5-year DMFS, or 7.5-year DSOS. For cell-based assays, mean±sem were used for data presentation. Differences were evaluated by Student's t-test to estimate statistical significance when two groups were compared and by ANOVA when three or more groups were compared. A value of p<0.05 was considered to be significant. For *in vivo* experiments, two way ANOVA with Bonferroni's test was used.

## SUPPLEMENTARY METHODS, FIGURES AND TABLE




