# Supplementary material for: Reduced ING1 levels in breast cancer promotes metastasis
Source: Oncotarget. 2014 May 19;5(12):4244–56. doi: 10.18632/oncotarget.1988 (PMC4147320; doi:10.18632/oncotarget.1988)
Supplement: Supplementary file 1 [file oncotarget-05-4244-s001.pdf]

Reduced ING1 levels in breast cancer promotes metastasis

Supplmentary Methods

Patient Cohort

532 subjects, with a median follow-up time of 82.1 months, met the criteria and had triplicate 0.6mm FFPE cores built into tissue microarrays (TMAs). While ER and PR ligand binding assays (LBA) were routinely performed during the diagnosis period for this cohort, all patients were treated with tamoxifen, as definitive evidence that ER poor patients did not respond to tamoxifen, was lacking at that time [1, 2]. Retrospective ER, PR and HER2 status was established using the Dako PharmDx ER and PR immunohistochemistry assays and the Herceptest immunohistochemistry assay, as per the manufacturer's instructions. ER+ or PR+ or Her2+ status was defined as maximum ER or PR Allred score <3 across triplicate cores, or an average Her2 ASCO score >2 across triplicate cores. Table 1 shows clinical-pathological characteristics of the subjects. All tissues were fixed and embedded using a standard protocol.

Microarrays

RNA from treated cells was isolated using Trizol (Invitrogen) and purified using the RNeasy Mini Kit (Qiagen). The microarray was designed on a Nimblegen platform (HD1) and contained oligonucleotide probes for about 19,000 protein-coding genes. Each transcript was represented by 1-5 probes and was selected from 5 databases (NCBI RefSeq, UCSC knowngenes, RNAdb 2.0, NRED, and UCRs) and specific references.

After cDNA preparation, the double stranded cDNA samples were labeled with Cy3 using the NimbleGen One-Color DNA Labeling Kit (Roche NimbleGen) and hybridized to the 12x135K mRNA expression microarray for 16 to 20 hours. Three technical replicates were performed for each sample and scanned with the Axon GenePix 4000B scanner (Molecular Devices Corporation). Raw data were analyzed using Aligent GeneSpring GX software (version 11.5.1). Volcano Plot filtering identified genes of interest based on ≥ 2-fold changes and by statistical analyses (P-value<0.05). Genetic association and pathway analyses were then done to identify candidate roles and functions of protein coding genes in biological pathways. Since ING1 expression was reduced in tumor samples, analyses were performed according to protocols from the DAVID database.

RNA isolation and Quantitative real time PCR

Total RNA from cells was isolated using RNeasy Kits (Qiagen) according to the manufacturer's guidelines and reverse transcribed using an ABI Reverse Transcription kit (Applied Biosystems). Real time PCR was carried out in triplicate using SYBR Select Mastermix (Invitrogen) on an Applied Biosystems 7900HT Fast Real-time PCR system using a standard protocol. GAPDH expression was used as a normalization control. The ΔCT method was used for analysis of real time PCR products.

Micro-computed tomography (µCT)

For scanning, the bones were placed in a special sample holder with 6 upright cylinders that fit one mouse hind limb each. The holder was placed in the µCT scanner (vivaCT 40, Scanco Medical, Switzerland) and a region of interest, in this case the whole limb, was selected following a scout-view of the samples in the holder. Approximately, 1000 tomographic images per stack were acquired in a period of 3 hours at 70 kVp (applied peak, 114 µA, and 200 ms integration time). The scan generated 555 cross-sectional slices that were used to reconstruct a three-dimensional (3D) image of the sample. For the analysis, a region of proximal tibia was used to determine the bone morphometric parameters bone volume/total volume (BV/TV), Cortical bone volume/total volume (Ct BV/TV) and bone mineral density (BMD) that were used to analyze the magnitude of tumor induced osteolysis.

References

1. Systemic treatment of early breast cancer by hormonal, cytotoxic, or immune therapy. 133 randomised trials involving 31,000 recurrences and 24,000 deaths among 75,000 women. Early Breast Cancer Trialists' Collaborative Group. Lancet. 1992; 339(8784):1-15.
2. Tamoxifen for early breast cancer: an overview of the randomised trials. Early Breast Cancer Trialists' Collaborative Group. Lancet. 1998; 351(9114):1451-1467.
3. Huang da W, Sherman BT and Lempicki RA. Systematic and integrative analysis of large gene lists using DAVID bioinformatics resources. Nature protocols. 2009; 4(1):44-57.

A

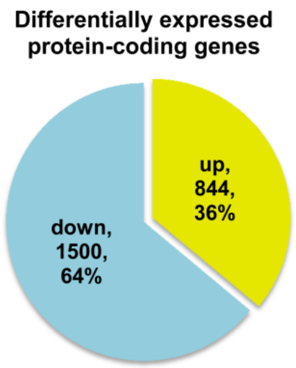

B

Term	P-Value
Breast cancer	8.0E-4
Colorectal cancer; Tourette syndrome; Bone density; Pregnancy loss, recurrent; Cleft lip without cleft palate; Juvenile polyposis; Cleft palate	8.7E-4
Diabetes, type 2; Nephropathy in other diseases	1.1E-3
Ovarian cancer	3.0E-3
Colon cancer	6.4E-3
Colorectal cancer	1.2E-2
Stomach cancer	2.3E-2
Atopy beta-lactam allergy	2.4E-2
Prostate cancer	4.4E-2
Colorectal cancer; Endometrial cancer	4.7E-2

C

#	Category	Term	Kappa
1	GENETIC_ASSOCIATION_DB_DISEASE	Breast cancer	1.00
2	GENETIC_ASSOCIATION_DB_DISEASE	Colorectal cancer	0.60
3	GENETIC_ASSOCIATION_DB_DISEASE	Lung cancer	0.42
4	GENETIC_ASSOCIATION_DB_DISEASE	Prostate cancer	0.41
5	GENETIC_ASSOCIATION_DB_DISEASE	Stomach cancer	0.34
6	GENETIC_ASSOCIATION_DB_DISEASE	Ovarian cancer	0.33
7	GENETIC_ASSOCIATION_DB_DISEASE	Bladder cancer	0.32
8	GOTERM_BP_FAT	Regulation of cell cycle	0.32
9	GENETIC_ASSOCIATION_DB_DISEASE	Esophageal cancer	0.30

Supplementary Figure 1: ING1b regulated genes. (A) Ectopic expression of ING1 reproducibly induced 844 and repressed 1,500 genes by >2-fold in three separate trials. (B) Analysis of the 1,500 repressed genes using the Genetic_Association_DB_Disease and analysis using DAVID [3]. Pathways with $p<0.05$ are shown. (C) Disease profiling of the 1,500 repressed genes by similarity score gave values of Kappa between 1.0 and 0.3 where 0.75-1.0 is very high, 0.5-0.75 is high, 0.25-0.5 is moderate and < 0.25 is a low score. Pathways showing scores above low are shown.

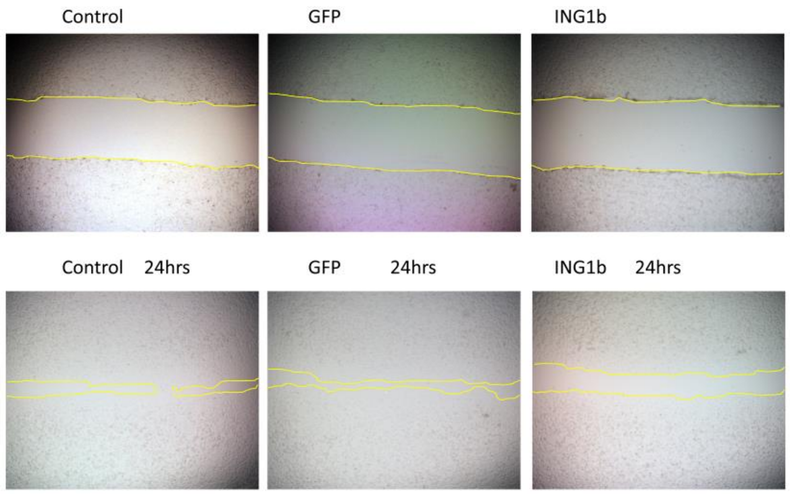

Supplementary Figure 2: Scratch wound healing assay. Monolayers of MDA-MB231 cells infected with GFP or ING1 expressing viral constructs were scratched and the amount of void space filled was visualized 24 hours later.

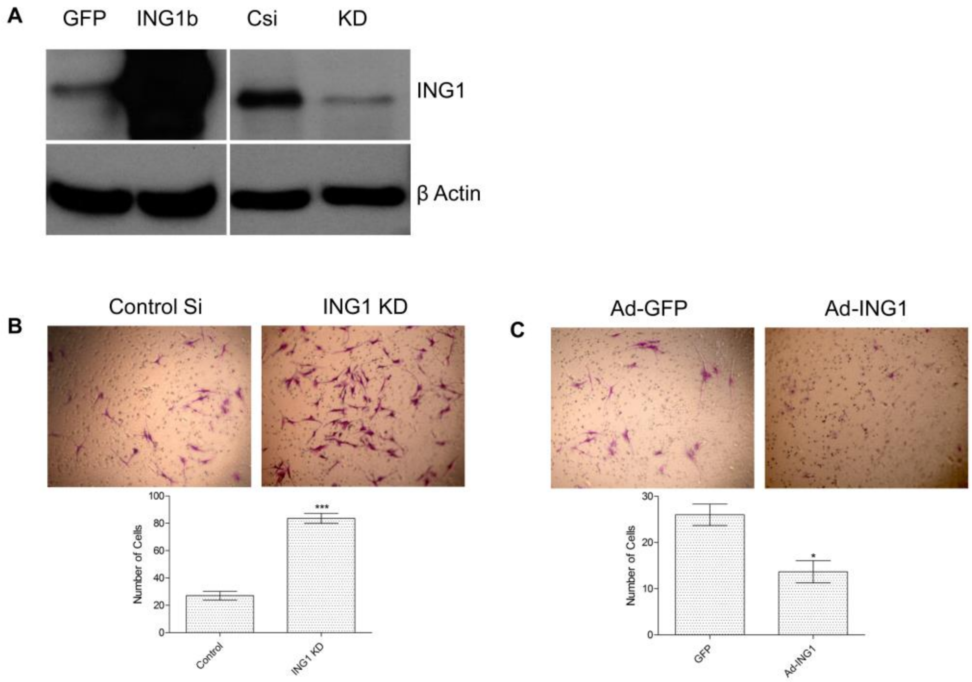

Supplementary Figure 3: ING1 inhibits invasion of HS68 fibroblasts. Representative images of matrigel invasion assays upon ING1 overexpression or knockdown in HS68 cells.

Supplementary Table 1: Complete list of genes showing significant differential expression in response to ING1 overexpression.

Table 1: Cohort Clinico-Pathologic Characteristics (Total n=532, Ing1 and ER/Her2 n=462)

		ER+/Her2-			ER- or Her2+		
**Characteristic**	**# of Cases (%)**	**Low Ing1**	**High Ing1**	**χ^2 p-value**	**Low Ing1**	**High Ing1**	**Fisher's Exact p-value**
**Age**							
Range	35.8-95.5						
Median	66						
< 53	81 (15)	18	49	0.298	1	2	1.000
≥ 53	451 (85)	121	242		11	18	
**Body Mass Index**							
Normal	136 (26)	36	74	0.869	5	7	0.747
Underweight	8 (1)	2	2		0	0	
Overweight	136 (26)	38	71		5	7	
Obese	107 (20)	29	62		2	1	
Unknown	145 (27)						
**Stage**							
I	233 (44)	54	134	0.345	1	7	0.239
II	163 (31)	48	86		7	6	
III	40 (8)	10	19		2	4	
IV	7 (1)	0	3		1	1	
Unknown	89 (17)						
**Tumor Grade**							
Low (1/2)	419 (79)	117	229	0.466	4	13	0.119
High (3)	68 (13)	14	35		7	5	
Unknown	45 (8)						
**Tumor Size**							
< 2cm	271 (51)	70	154	0.629	1	10	0.019*
≥ 2cm	221 (42)	59	117		10	8	
Unknown	40 (7)						
**Lymph Node Status**							
Negative	342 (64)	81	192	0.085	3	13	0.205
Positive	117 (22)	38	59		4	5	
Unknown	73 (14)						
**ER status**							
No	16 (3)	0	0	nd	6	9	1.000
Yes	473 (89)	139	291		6	11	
Unknown	43 (8)						
**PR status**							
No	56 (11)	12	24	0.937	4	10	0.466
Yes	416 (78)	118	243		7	9	
Unknown	60 (11)						
**Her2 status**							
No	505 (95)	139	291	nd	5	8	1.000
Yes	22 (4)	0	0		7	12	
Unknown	5 (1)						
**Any Recurrence**							
No	437 (82)	114	234	0.692	6	18	0.030*
Yes	95 (18)	25	57		6	2	
**Distant Recurrence**							
No	458 (86)	117	248	0.776	6	20	0.001*
Yes	74 (14)	22	43		6	0	
**Radiation Therapy**							
No	199 (37)	45	117	0.079	7	8	0.710
Yes	319 (60)	93	165		5	10	
Unknown	14 (3)						
