# Supplementary material for: Reduced ING1 levels in breast cancer promotes metastasis
Source: Oncotarget. 2014 May 19;5(12):4244–56. doi: 10.18632/oncotarget.1988 (PMC4147320; doi:10.18632/oncotarget.1988)
Supplement: Supplementary file 2 [file oncotarget-05-4244-s002.pdf]

SEQ_ID	GeneSymbol	p-value	FoldChange	regulation	EntrezGene	Chromosome	Strand
NM_006142	SFN	2.60E-04	47.471848	up	2810	chr1	+
NM_005557	KRT16	5.78E-05	46.86887	up	3868	chr17	-
NM_002928	RGS16	1.26E-04	40.40026	up	6004	chr1	-
NM_006072	CCL26	4.73E-05	26.032455	up	10344	chr7	-
NM_005382	NEFM	5.46E-05	22.885656	up	4741	chr8	+
NM_001130046	CCL20	1.97E-05	22.46914	up	6364	chr2	+
NM_015675	GADD45B	5.65E-05	16.336153	up	4616	chr19	+
NM_000526	KRT14	6.23E-06	15.570794	up	3861	chr17	-
NM_003811	TNFSF9	5.60E-04	15.554252	up	8744	chr19	+
NM_014400	LYPD3	4.54E-06	14.959286	up	27076	chr19	-
NM_002135	NR4A1	3.52E-04	13.816516	up	3164	chr12	+
NM_173354	SNF1LK	0.00199493	12.928187	up	150094	chr21	-
NM_001032409	OAS1	1.86E-04	11.293347	up	4938	chr12	+
NM_002523	NPTX2	4.10E-04	11.212401	up	4885	chr7	+
NM_005252	FOS	5.72E-04	10.844219	up	2353	chr14	+
NM_000422	KRT17	3.45E-04	10.6585865	up	3872	chr17	-
NM_004031	IRF7	6.25E-05	10.256829	up	3665	chr11	-
NM_005985	SNAI1	0.00267544	9.931202	up	6615	chr20	+
NM_002281	KRT81	6.75E-04	9.92921	up	3887	chr12	-
NM_145288	ZNF342	4.41E-04	9.625938	up	162979	chr19	-
NM_003518	HIST1H2BG	5.28E-04	9.523214	up	8339	chr6	-
NM_003468	FZD5	1.97E-04	9.421069	up	7855	chr2	-
NM_002534	OAS1	0.0037811	9.062821	up	4938	chr12	+
NM_198538	SBSN	2.77E-05	8.254546	up	374897	chr19	-
NM_001081492	KRT80	2.95E-04	8.193607	up	144501	chr12	-
NM_001572	IRF7	4.72E-04	7.5172544	up	3665	chr11	-
NM_024518	ULBP3	1.27E-04	7.497238	up	79465	chr6	-
NM_001145774	GPR56	1.29E-05	7.3240204	up	9289	chr16	+
NM_133467	CITED4	4.66E-04	7.323965	up	163732	chr1	-
NM_006145	DNAJB1	4.05E-05	7.13665	up	3337	chr19	-
NM_025217	ULBP2	0.00174524	6.819724	up	80328	chr6	+
NM_004952	EFNA3	1.46E-04	6.710254	up	1944	chr1	+
NM_004418	DUSP2	0.00118108	6.6276417	up	1844	chr2	-
NM_004925	AQP3	2.92E-04	6.312436	up	360	chr9	-
NM_000735	CGA	0.00586137	6.3077416	up	1081	chr6	-
NM_001145771	GPR56	4.73E-05	6.29032	up	9289	chr16	+
NM_013376	SERTAD1	2.21E-04	6.2857184	up	29950	chr19	-
NM_002866	RAB3A	2.89E-06	6.2484937	up	5864	chr19	-
NM_016352	CPA4	1.80E-04	6.227758	up	51200	chr7	+
NM_203444	ABCB9	4.15E-04	6.019358	up	23457	chr12	-
NM_004864	GDF15	0.00156725	5.976982	up	9518	chr19	+
NM_012342	BAMBI	1.94E-04	5.946053	up	25805	chr10	+
NM_016323	HERC5	7.19E-04	5.8358483	up	51191	chr4	+
NM_019625	ABCB9	0.00130527	5.7683	up	23457	chr12	-
NM_004252	SLC9A3R1	0.00121211	5.668831	up	9368	chr17	+
NM_031449	ZMIZ2	5.61E-05	5.6597996	up	83637	chr7	+
NM_003528	HIST2H2BE	1.85E-04	5.6536264	up	8349	chr1	-
NM_001335	CTSW	2.88E-04	5.6174965	up	1521	chr11	+

NM_001042680	C19orf28	9.35E-04	5.558996	up	126321	chr19	-
NM_001085425	ARSA	0.00153001	5.4041467	up	410	chr22	-
NM_181900	STARD5	6.25E-05	5.2870326	up	80765	chr15	-
NM_025080	ASRGL1	2.02E-04	5.252914	up	80150	chr11	+
NM_001040439	MAPK8IP3	0.00393616	5.139602	up	23162	chr16	+
NM_005345	HSPA1A	7.32E-04	5.0716424	up	3303	chr6_cox_hap1	+
NM_015404	DFNB31	0.00107896	5.0685973	up	25861	chr9	-
NM_001017402	LAMB3	3.02E-04	5.0591393	up	3914	chr1	-
NM_000064	C3	0.00178214	5.055205	up	718	chr19	-
NM_000641	IL11	1.15E-04	4.9224057	up	3589	chr19	-
NM_002038	IFI6	0.0104574	4.8925657	up	2537	chr1	-
NM_014807	TMEM24	0.00704917	4.872669	up	9854	chr11	+
NM_198445	RINL	0.0127601	4.8696265	up	126432	chr19	-
NM_152657	GGN	4.50E-04	4.8372993	up	199720	chr19	-
NM_003311	PHLDA2	0.00485985	4.8364825	up	7262	chr11	-
NM_001144925	MX1	0.02343049	4.8120103	up	4599	chr21	+
NM_004335	BST2	0.00169062	4.810347	up	684	chr19	-
NM_024628	SLC12A8	2.23E-04	4.7583256	up	84561	chr3	-
NM_001024213	S100A13	0.00120257	4.6644807	up	6284	chr1	-
NM_018645	HES6	0.00171403	4.6567535	up	55502	chr2	-
NM_015201	BOP1	3.80E-05	4.650151	up	23246	chr8	-
NM_005165	ALDOC	1.01E-06	4.644434	up	230	chr17	-
NM_022872	IFI6	8.09E-05	4.637399	up	2537	chr1	-
NM_014391	ANKRD1	7.81E-04	4.60108	up	27063	chr10	-
NM_001040161	C16orf13	0.00232558	4.5350847	up	84326	chr16	-
NM_020376	PNPLA2	8.87E-04	4.499172	up	57104	chr11	+
NM_174929	ZMIZ2	8.35E-04	4.4738317	up	83637	chr7	+
NM_017842	FLJ20489	0.00334467	4.4402003	up	55652	chr12	+
NM_002632	PGF	0.00180852	4.4335675	up	5228	chr14	-
NM_198188	ASTN2	0.00279693	4.334596	up	23245	chr9	-
NM_001006665	RPS6KA1	0.00127695	4.321809	up	6195	chr1	+
NM_015683	ARRDC2	1.22E-04	4.2975764	up	27106	chr19	+
NM_001037335	PRIC285	0.00377851	4.265735	up	85441	chr20	-
NM_145664	SPANXB2	0.00635132	4.254309	up	64694	chrX	+
NM_002206	ITGA7	0.00467827	4.252084	up	3679	chr12	-
NM_022153	C10orf54	0.00813613	4.2354627	up	64115	chr10	-
NM_001040280	CD83	0.00312412	4.2096086	up	9308	chr6	+
NM_003841	TNFRSF10C	2.47E-04	4.1755657	up	8794	chr8	+
NM_182958	MYST1	0.01915508	4.170395	up	84148	chr16	+
NM_020040	TUBB4Q	3.54E-04	4.1645713	up	56604	chr4	-
NM_005101	ISG15	1.96E-04	4.1626625	up	9636	chr1	+
NM_001002029	C4B	0.00133007	4.1379967	up	721	chr6_cox_hap1	+
NM_000247	MICA	4.85E-04	4.1029787	up	4276	chr6	+
NM_016368	ISYNA1	3.71E-04	4.098904	up	51477	chr19	-
NM_001005785	DAZ2	0.00528911	4.096633	up	57055	chrY	+
NM_000033	ABCD1	2.45E-04	4.05111	up	215	chrX	+
NM_006763	BTG2	1.38E-05	4.034714	up	7832	chr1	+
NM_022873	IFI6	7.30E-04	3.989208	up	2537	chr1	-
NM_002905	RDH5	3.65E-04	3.9399962	up	5959	chr12	+

NM_198707	HSD11B1L	0.00350635	3.9391382	up	374875 chr19	+
NM_017914	C19orf24	9.06E-06	3.9295194	up	55009 chr19	+
NM_001080453	INTS1	1.21E-04	3.8925235	up	26173 chr7	-
NM_177457	LYNX1	6.43E-05	3.8278034	up	66004 chr8	-
NM_001130183	DNAJA4	0.01875345	3.8271394	up	55466 chr15	+
NM_021731	C19orf28	0.02554223	3.8182812	up	126321 chr19	-
NM_002543	OLR1	7.75E-05	3.8052793	up	4973 chr12	-
NM_014291	GCAT	8.40E-04	3.792006	up	23464 chr22	+
NM_052943	FAM46B	0.00104101	3.7624578	up	115572 chr1	-
NM_001013257	BCAM	0.00847048	3.752468	up	4059 chr19	+
NM_004721	MAP3K13	8.68E-04	3.752355	up	9175 chr3	+
NM_152892	DKFZp434K1:	1.64E-04	3.7465863	up	222229 chr7	+
NM_002298	LCP1	4.97E-04	3.7458038	up	3936 chr13	-
NM_001135057	LRRC15	1.04E-04	3.7358878	up	131578 chr3	-
NM_019034	RHOF	2.03E-04	3.7280428	up	54509 chr12	-
NM_001126057	DMKN	0.01767816	3.7056158	up	93099 chr19	-
NM_014634	PPM1F	0.00244725	3.6996706	up	9647 chr22	-
NM_006762	LAPTM5	7.24E-04	3.6979284	up	7805 chr1	-
NM_198516	GALNTL4	0.00883041	3.691139	up	374378 chr11	-
NM_001126058	DMKN	0.00834228	3.6896815	up	93099 chr19	-
NM_138414	CCDC101	3.87E-04	3.6761649	up	112869 chr16	+
NM_198202	POP5	0.00656345	3.6673083	up	51367 chr12	-
NM_006675	TSPAN9	0.00136687	3.6526613	up	10867 chr12	+
NM_005694	COX17	6.12E-05	3.6516232	up	10063 chr3	-
NM_178031	TMEM132A	4.07E-04	3.648366	up	54972 chr11	+
NM_022464	SIL1	0.02687102	3.6369908	up	64374 chr5	-
NM_015104	KIAA0404	0.00281499	3.6365402	up	23130 chr11	-
NM_000331	SAA1	0.01061126	3.6358948	up	6288 chr11	+
NM_001039211	ATAD3C	0.00223706	3.621991	up	219293 chr1	+
NM_001141974	ATP13A2	0.00537982	3.6094308	up	23400 chr1	-
NM_001242	CD27	0.00451717	3.6054091	up	939 chr12	+
NM_012267	HSPBP1	2.06E-04	3.6042118	up	23640 chr19	-
NM_018234	STEAP3	6.73E-04	3.601461	up	55240 chr2	+
NM_181701	QSOX2	0.02217923	3.595494	up	169714 chr9	-
NM_000024	ADRB2	0.00343261	3.5895605	up	154 chr5	+
NM_199245	VAMP1	0.00623459	3.5181394	up	6843 chr12	-
NM_170726	ALDH4A1	6.10E-04	3.514332	up	8659 chr1	-
NM_018948	ERRFI1	1.71E-04	3.4958727	up	54206 chr1	-
NM_001657	AREG	7.71E-05	3.4956727	up	374 chr4	+
NM_183008	UBXN11	4.66E-05	3.480647	up	91544 chr1	-
NM_001442	FABP4	0.00156289	3.4800742	up	2167 chr8	-
NM_033049	MUC13	0.00855102	3.4709063	up	56667 chr3	-
NM_001130979	DYSF	0.03129629	3.469913	up	8291 chr2	+
NM_004093	EFNB2	0.00330327	3.4697638	up	1948 chr13	-
NM_013976	GCDH	1.01E-04	3.4590428	up	2639 chr19	+
NM_018009	TAPBPL	0.01784009	3.4565308	up	55080 chr12	+
NM_177939	P4HTM	0.01443748	3.451195	up	54681 chr3	+
NM_003801	GPAA1	2.57E-04	3.4450524	up	8733 chr8	+
NM_005581	BCAM	0.00406957	3.4205675	up	4059 chr19	+

NM_003273	TM7SF2	2.50E-05	3.4099545	up	7108	chr11	+
NM_001040874	HIST2H2AA4	2.73E-04	3.4023645	up	723790	chr1	+
NM_001098785	FAM89B	5.05E-05	3.402351	up	23625	chr11	+
NM_001077494	NFKB2	0.00509659	3.3874767	up	4791	chr10	+
NM_005623	CCL8	0.01999329	3.3853467	up	6355	chr17	+
NM_177477	LYNX1	0.00382951	3.3754754	up	66004	chr8	-
NM_032648	C1orf90	0.00187036	3.3739586	up	84734	chr1	+
NM_004603	STX1A	7.39E-04	3.3723419	up	6804	chr7	-
NM_031923	TAF3	2.97E-05	3.3421113	up	83860	chr10	+
NM_001433	ERN1	0.00149773	3.3405323	up	2081	chr17	-
NM_004613	TGM2	0.0050157	3.3371444	up	7052	chr20	-
NM_015983	UBE2D4	7.35E-05	3.3366525	up	51619	chr7	+
NM_001042461	TRAPPC5	1.28E-04	3.3253808	up	126003	chr19	+
NM_002616	PER1	1.56E-04	3.3238864	up	5187	chr17	-
NM_000156	GAMT	1.22E-04	3.3222091	up	2593	chr19	-
NM_003673	TCAP	0.00125176	3.3085895	up	8557	chr17	+
NM_133373	PLCD3	1.43E-04	3.2964406	up	113026	chr17	-
NM_001099281	HEATR7A	0.02342081	3.2955124	up	727957	chr8	+
NM_134470	IL1RAP	0.00456846	3.292486	up	3556	chr3	+
NM_033015	FASTK	0.00930569	3.2828538	up	10922	chr7	-
NM_001040097	MOSPD3	0.00627381	3.2801967	up	64598	chr7	+
NM_006622	PLK2	5.38E-04	3.2780745	up	10769	chr5	-
NM_138432	SDSL	8.22E-04	3.2708666	up	113675	chr12	+
NM_001098784	FAM89B	0.00149773	3.2502465	up	23625	chr11	+
NM_020365	EIF2B3	5.82E-05	3.2470467	up	8891	chr1	-
NM_000729	CCK	0.04770354	3.2459877	up	885	chr3	-
NM_013439	PILRA	0.00124713	3.2297008	up	29992	chr7	+
NM_003082	SNAPC1	8.84E-04	3.2266898	up	6617	chr14	+
NM_001079803	GAA	0.01175742	3.212042	up	2548	chr17	+
NM_145182	PYCARD	0.03873961	3.2011173	up	29108	chr16	-
NM_004910	PITPNM1	0.0025864	3.194881	up	9600	chr11	-
NM_002081	GPC1	9.87E-04	3.1826909	up	2817	chr2	+
NM_032433	ZNF333	0.00829146	3.1817844	up	84449	chr19	+
NM_014424	HSPB7	0.00544337	3.180733	up	27129	chr1	-
NM_004049	BCL2A1	0.01008133	3.1803892	up	597	chr15	-
NM_005341	ZBTB48	6.81E-04	3.175889	up	3104	chr1	+
NM_001002249	ANAPC11	0.00182349	3.169349	up	51529	chr17	+
NM_032868	MPND	3.22E-04	3.1634057	up	84954	chr19	+
NM_002273	KRT8	6.20E-04	3.161477	up	3856	chr12	-
NM_032319	C2orf7	8.82E-04	3.1504703	up	84279	chr2	-
NM_212492	GPS1	3.82E-04	3.1464326	up	2873	chr17	+
NM_002774	KLK6	0.01437637	3.14563	up	5653	chr19	-
NM_000148	FUT1	0.04305741	3.1361196	up	2523	chr19	-
NM_014506	TOR1B	7.75E-04	3.133464	up	27348	chr9	+
NM_015133	MAPK8IP3	0.00335142	3.1332405	up	23162	chr16	+
NM_032520	GNPTG	5.64E-04	3.1303008	up	84572	chr16	+
NM_031466	NIBP	0.00112239	3.1222348	up	83696	chr8	-
NM_052990	IFT122	7.71E-04	3.1177254	up	55764	chr3	+
NM_001025604	ARRDC2	0.01730092	3.117641	up	27106	chr19	+

NM_005072	SLC12A4	4.95E-05	3.1092203	up	6560	chr16	-
NM_004431	EPHA2	3.92E-05	3.1007311	up	1969	chr1	-
NM_001130867	ERCC2	0.00193176	3.0994053	up	2068	chr19	-
NM_001012759	C16orf84	2.41E-04	3.0880613	up	348180	chr16	+
NM_007343	PRSS3	0.00182799	3.083066	up	5646	chr9	+
NM_001100817	TCEB3CL	0.03029101	3.079014	up	728929	chr18	-
NM_014367	C3orf28	2.32E-04	3.0744104	up	26355	chr3	+
NM_005343	HRAS	1.58E-04	3.0733213	up	3265	chr11	-
NM_058190	C21orf70	0.01514483	3.069192	up	85395	chr21	+
NM_005393	PLXNB3	0.00164755	3.0658047	up	5365	chrX	+
NM_003550	MAD1L1	3.28E-04	3.065757	up	8379	chr7	-
NM_022164	TINAGL1	0.04324013	3.0605066	up	64129	chr1	+
NM_004269	CRSP8	0.00149846	3.0585692	up	9442	chr9	-
NM_000709	BCKDHA	9.81E-04	3.0583527	up	593	chr19	+
NM_153741	DPM3	4.69E-04	3.0502284	up	54344	chr1	-
NM_017522	LRP8	0.02901298	3.049161	up	7804	chr1	-
NM_013366	ANAPC2	0.00724775	3.0485976	up	29882	chr9	-
NM_007183	PKP3	0.02862635	3.0445375	up	11187	chr11	+
NM_016234	ACSL5	5.81E-04	3.0407925	up	51703	chr10	+
NM_001017963	HSP90AA1	4.59E-04	3.0229294	up	3320	chr14	-
NM_016538	SIRT7	0.00213639	3.0093815	up	51547	chr17	-
NM_001002248	ANAPC11	0.02139911	3.009131	up	51529	chr17	+
NM_005346	HSPA1B	0.0069255	3.0087512	up	3304	chr6_cox_hap1	+
NM_004106	FCER1G	0.00857588	3.007078	up	2207	chr1	+
NM_145017	C11orf66	0.00912698	3.0032132	up	220004	chr11	+
NM_001001977	ATP5E	0.04127635	2.9930708	up	514	chr20	-
NM_016222	DDX41	1.02E-04	2.98956	up	51428	chr5	-
NM_001040162	C16orf13	0.00282049	2.9866002	up	84326	chr16	-
NM_005166	APLP1	0.04355142	2.9832764	up	333	chr19	+
NM_012090	MACF1	0.01393783	2.9804597	up	23499	chr1	+
NM_032326	TMEM175	8.19E-04	2.9786742	up	84286	chr4	+
NM_005458	GABBR2	0.0242939	2.9765465	up	9568	chr9	-
NM_001012634	IL32	0.01039165	2.9718916	up	9235	chr16	+
NM_020158	EXOSC5	0.00439757	2.971265	up	56915	chr19	-
NM_013365	GGA1	9.67E-04	2.9688148	up	26088	chr22	+
NM_003020	SCG5	0.00871802	2.9680252	up	6447	chr15	+
NM_001097620	TMEM184A	0.00307177	2.9585512	up	202915	chr7	-
NM_006688	C1QL1	0.00284463	2.9567955	up	10882	chr17	-
NM_000548	TSC2	6.48E-05	2.9511132	up	7249	chr16	+
NM_176795	HRAS	6.00E-04	2.946663	up	3265	chr11	-
NM_001143780	SLC25A39	0.00718646	2.9415698	up	51629	chr17	-
NM_003784	SERPINB7	0.00115214	2.9351115	up	8710	chr18	+
NM_014347	ZNF324	5.67E-04	2.9327745	up	25799	chr19	+
NM_001018109	PIR	0.01585735	2.9315999	up	8544	chrX	-
NM_002972	SBF1	4.59E-04	2.930664	up	6305	chr22	-
NM_145201	NAPRT1	3.45E-04	2.9298098	up	93100	chr8	-
NM_001670	ARVCF	0.00293827	2.9292336	up	421	chr22	-
NM_000920	PC	0.04417141	2.925001	up	5091	chr11	-
NM_001004426	PLA2G6	6.61E-04	2.9249246	up	8398	chr22	-

NM_001005909	IP6K2	0.01083706	2.9247072	up	51447	chr3	-
NM_021063	HIST1H2BD	0.02726985	2.9218338	up	3017	chr6	+
NM_001011669	CHCHD7	0.002008	2.9187179	up	79145	chr8	+
NM_001002901	FCRLB	0.01971415	2.9182396	up	127943	chr1	+
NM_198187	ASTN2	0.0079878	2.9128952	up	23245	chr9	-
NM_001077493	NFKB2	0.0068341	2.9125793	up	4791	chr10	+
NM_001135217	LRRC23	0.0058437	2.9100082	up	10233	chr12	+
NM_000076	CDKN1C	2.11E-04	2.9090917	up	1028	chr11	-
NM_201398	FLAD1	0.00709594	2.9018962	up	80308	chr1	+
NM_002727	SRGN	0.00511918	2.9009736	up	5552	chr10	+
NM_001130442	HRAS	0.00327993	2.899068	up	3265	chr11	-
NM_001012631	IL32	0.00583203	2.8954082	up	9235	chr16	+
NM_003006	SELPLG	0.01808531	2.8873444	up	6404	chr12	-
NM_004045	ATOX1	2.79E-04	2.8868558	up	475	chr5	-
NM_145167	PIGM	0.00104649	2.8845153	up	93183	chr1	-
NM_002541	OGDH	1.15E-05	2.880571	up	4967	chr7	+
NM_016286	DCXR	3.52E-04	2.880032	up	51181	chr17	-
NM_001085426	ARSA	4.32E-04	2.8791242	up	410	chr22	-
NM_005319	HIST1H1C	3.16E-05	2.8737223	up	3006	chr6	-
NM_000400	ERCC2	0.00111607	2.8716447	up	2068	chr19	-
NM_001005416	2-Mar	0.00787431	2.865486	up	51257	chr19	+
NM_002953	RPS6KA1	0.00335883	2.8645134	up	6195	chr1	+
NM_032490	C14orf142	7.40E-04	2.8637512	up	84520	chr14	-
NM_001105540	DGKZ	3.36E-06	2.8614068	up	8525	chr11	+
NM_139179	DAGLB	0.00314988	2.8582857	up	221955	chr7	-
NM_005854	RAMP2	0.00363265	2.8580787	up	10266	chr17	+
NM_003748	ALDH4A1	0.00307599	2.8576527	up	8659	chr1	-
NM_001039182	BOLA2B	4.64E-04	2.8558965	up	654483	chr16	-
NM_177938	P4HTM	0.01174464	2.8525474	up	54681	chr3	+
NM_001024943	ASL	0.00288805	2.845805	up	435	chr7	+
NM_182614	FAM70B	5.00E-04	2.836004	up	348013	chr13	-
NM_013321	SNX8	3.50E-04	2.833006	up	29886	chr7	-
NM_182527	CABP7	5.82E-05	2.8315158	up	164633	chr22	+
NM_021076	NEFH	4.44E-04	2.8285038	up	4744	chr22	+
NM_198098	AQP1	0.00452374	2.8280084	up	358	chr7	+
NM_032564	DGAT2	0.01489361	2.8146763	up	84649	chr11	+
NM_001019	RPS15A	0.00132482	2.8138158	up	6210	chr16	-
NM_001081491	NXF1	0.00789165	2.8131986	up	10482	chr11	-
NM_015939	CGI-09	0.00792673	2.8102062	up	51605	chr20	-
NM_198383	CACNA1G	0.00818028	2.80813	up	8913	chr17	+
NM_020170	NCLN	2.60E-04	2.7978096	up	56926	chr19	+
NM_002256	KISS1	0.00659079	2.7975729	up	3814	chr1	-
NM_152339	SPATA2L	9.39E-04	2.7963645	up	124044	chr16	-
NM_001011667	CHCHD7	0.0253096	2.7961197	up	79145	chr8	+
NM_006929	SKIV2L	0.00390088	2.7929108	up	6499	chr6	+
NM_015386	COG4	3.37E-04	2.792119	up	25839	chr16	-
NM_001040160	C16orf13	0.00142774	2.7918403	up	84326	chr16	-
NM_001105564	CCHCR1	0.00323234	2.788629	up	54535	chr6	-
NM_016830	VAMP1	5.45E-04	2.7858396	up	6843	chr12	-

NM_198955	MGAT5B	0.00182962	2.777745	up	146664	chr17	+
NM_015953	NOSIP	5.82E-04	2.7730372	up	51070	chr19	-
NM_001017389	SULT1A4	0.00482903	2.7701848	up	445329	chr16	+
NM_015179	RRP12	0.00146566	2.7700162	up	23223	chr10	-
NM_004750	CRLF1	0.0180662	2.760811	up	9244	chr19	-
NM_015399	BRMS1	0.00408128	2.7589622	up	25855	chr11	-
NM_183241	C9orf142	5.36E-04	2.757518	up	286257	chr9	+
NM_001114382	TSC2	2.81E-04	2.757188	up	7249	chr16	+
NM_020196	XAB2	0.00692752	2.75598	up	56949	chr19	-
NM_001024807	APLP1	0.00710837	2.7537146	up	333	chr19	+
NM_139235	NOL6	2.08E-04	2.7537074	up	65083	chr9	-
NM_002463	MX2	0.02242158	2.7507	up	4600	chr21	+
NM_016564	CEND1	9.20E-04	2.7501318	up	51286	chr11	-
NM_016567	BCCIP	0.01378989	2.7469006	up	56647	chr10	+
NM_004584	RAD9A	0.00270288	2.7458186	up	5883	chr11	+
NM_001142547	RPUSD3	0.04405528	2.7445781	up	285367	chr3	-
NM_006396	SSSCA1	0.00722722	2.7396216	up	10534	chr11	+
NM_001124767	LOC440957	0.00523976	2.733294	up	440957	chr3	+
NM_001500	GMDS	0.01958611	2.7314155	up	2762	chr6	-
NM_024959	SLC24A6	0.02101912	2.726773	up	80024	chr12	-
NM_020182	TMEPAI	0.01478542	2.726518	up	56937	chr20	-
NM_004328	BCS1L	0.00698838	2.7241578	up	617	chr2	+
NM_021197	WFDC1	0.00231345	2.7194405	up	58189	chr16	+
NM_001040716	PC	0.00286631	2.7193155	up	5091	chr11	-
NM_145196	LIPT1	0.00757085	2.719078	up	51601	chr2	+
NM_016243	CYB5R1	1.99E-05	2.715431	up	51706	chr1	-
NM_014427	CPNE7	0.00168356	2.7141173	up	27132	chr16	+
NM_182556	SLC25A45	0.00441004	2.7124808	up	283130	chr11	-
NM_017884	PINX1	0.00499239	2.7116373	up	54984	chr8	-
NM_002103	GYS1	0.00289846	2.7108593	up	2997	chr19	-
NM_007254	PNKP	1.12E-05	2.7063384	up	11284	chr19	-
NM_001985	ETFB	5.00E-05	2.7049415	up	2109	chr19	-
NM_032355	MON1A	1.57E-04	2.7047045	up	84315	chr3	-
NM_199132	ZNF468	9.55E-04	2.7027495	up	90333	chr19	-
NM_015629	PRPF31	2.54E-04	2.702075	up	26121	chr19	+
NM_145330	MRPL33	0.01101283	2.6971283	up	9553	chr2	+
NM_025236	RNF39	0.03359787	2.6968966	up	80352	chr6_qbl_hap2	-
NM_145754	KIFC2	1.07E-04	2.6957042	up	90990	chr8	+
NM_001009811	AZI1	0.00117519	2.6927013	up	22994	chr17	-
NM_001008223	C1QL4	0.0142259	2.6918178	up	338761	chr12	-
NM_004289	NFE2L3	0.03436866	2.6917503	up	9603	chr7	+
NM_138431	MFSD3	8.99E-04	2.6917458	up	113655	chr8	+
NM_001142936	DAGLB	8.36E-04	2.6878288	up	221955	chr7	-
NM_005001	NDUFA7	9.03E-04	2.6875818	up	4701	chr19	-
NM_001126337	TUFT1	0.01354424	2.6870027	up	7286	chr1	+
NM_130443	DPP3	8.00E-04	2.6777856	up	10072	chr11	+
NM_032450	HEATR7A	0.01661686	2.6755104	up	727957	chr8	+
NM_020127	TUFT1	0.00287245	2.6752062	up	7286	chr1	+
NM_000152	GAA	0.00342828	2.675063	up	2548	chr17	+

NM_007108	TCEB2	3.30E-05	2.672705 up	6923	chr16	-
NM_021259	TMEM8	0.0166537	2.6723287 up	58986	chr16	-
NM_001134492	HS2ST1	0.00835572	2.6705513 up	9653	chr1	+
NM_015853	LOC51035	0.00109819	2.6660068 up	51035	chr11	-
NM_001615	ACTG2	0.02362301	2.6651893 up	72	chr2	+
NM_001135208	FKBP2	0.02159596	2.659962 up	2286	chr11	+
NM_001002913	PTRH1	0.00431104	2.6597261 up	138428	chr9	-
NM_000041	APOE	0.01696506	2.6527414 up	348	chr19	+
NM_018973	DPM3	6.27E-04	2.6496255 up	54344	chr1	-
NM_005672	PSCA	0.00344414	2.6478937 up	8000	chr8	+
NM_001823	CKB	3.31E-05	2.6477923 up	1152	chr14	-
NM_024681	KCTD17	5.42E-04	2.6477468 up	79734	chr22	+
NM_001299	CNN1	0.01304485	2.639783 up	1264	chr19	+
NM_024324	CRELD2	0.00113625	2.6371388 up	79174	chr22	+
NM_004283	RAB3D	0.0467211	2.6363072 up	9545	chr19	-
NM_020812	DOCK6	7.54E-04	2.635424 up	57572	chr19	-
NM_001126056	DMKN	0.00290783	2.6344712 up	93099	chr19	-
NM_001080503	LOC126075	0.01638706	2.6335492 up	126075	chr19	+
NM_001144757	SCG5	0.0205719	2.6325552 up	6447	chr15	+
NM_000528	MAN2B1	0.00269714	2.630008 up	4125	chr19	-
NM_203286	PVRL1	0.00489177	2.629909 up	5818	chr11	-
NM_172088	TNFSF13	0.03403729	2.6283362 up	8741	chr17	+
NM_002889	RARRES2	0.00209986	2.626997 up	5919	chr7	-
NM_022808	SNRPN	5.00E-04	2.6266792 up	6638	chr15	+
NM_000159	GCDH	0.00726706	2.6254656 up	2639	chr19	+
NM_003840	TNFRSF10D	0.00170548	2.624749 up	8793	chr8	-
NM_014578	RHOD	0.00145232	2.6234307 up	29984	chr11	+
NM_153633	HOXC4	0.02043819	2.622524 up	3221	chr12	+
NM_001001671	MAP3K15	5.31E-04	2.616607 up	389840	chrX	-
NM_000854	GSTT2	0.00188733	2.615808 up	2953	chr22	-
NM_024598	C16orf57	1.35E-04	2.6154828 up	79650	chr16	+
NM_001687	ATP5D	0.00175976	2.6153169 up	513	chr19	+
NM_174983	C19orf28	0.03663361	2.6151536 up	126321	chr19	-
NM_016208	VPS28	5.16E-05	2.614209 up	51160	chr8	-
NM_019021	C11orf71	2.13E-04	2.6117985 up	54494	chr11	-
NM_138442	CCDC124	5.74E-04	2.6110606 up	115098	chr19	+
NM_012254	SLC27A5	0.02753017	2.6079943 up	10998	chr19	-
NM_002573	PAFAH1B3	5.42E-04	2.6047838 up	5050	chr19	-
NM_001047434	DPH3	0.00409353	2.603478 up	285381	chr3	-
NM_001004356	FGFRL1	0.01520918	2.6026204 up	53834	chr4	+
NM_138340	ABHD3	0.03407735	2.6023502 up	171586	chr18	-
NM_015354	NUP188	7.99E-05	2.601872 up	23511	chr9	+
NM_004146	NDUFB7	1.07E-04	2.5959897 up	4713	chr19	-
NM_001024944	ASL	0.0030365	2.5940974 up	435	chr7	+
NM_138720	HIST1H2BD	0.01137914	2.5934057 up	3017	chr6	+
NM_001142799	C17orf49	0.00233583	2.591213 up	124944	chr17	+
NM_001640	APEH	0.00527926	2.5905468 up	327	chr3	+
NM_005469	ACOT8	8.24E-05	2.5904305 up	10005	chr20	-
NM_021937	EEFSEC	0.00473988	2.5884328 up	60678	chr3	+

NM_025151	RAB11FIP1	0.00324766	2.5875552	up	80223	chr8	-
NM_182980	OSGIN1	0.02664476	2.5860488	up	29948	chr16	+
NM_001034833	NHP2	0.01528338	2.58503	up	55651	chr5	-
NM_001018136	NME1-NME2	7.14E-04	2.584439	up	654364	chr17	+
NM_003789	TRADD	4.57E-04	2.5836685	up	8717	chr16	-
NM_001099280	HEATR7A	0.01602613	2.583326	up	727957	chr8	+
NM_004040	RHOB	6.63E-04	2.5810413	up	388	chr2	+
NM_030593	SIRT2	2.57E-04	2.5797844	up	22933	chr19	-
NM_007059	KPTN	0.0198742	2.575721	up	11133	chr19	-
NM_203285	PVRL1	9.18E-05	2.5731864	up	5818	chr11	-
NM_133180	EPS8L1	0.01422111	2.5729685	up	54869	chr19	+
NM_001375	DNASE2	0.00310477	2.572052	up	1777	chr19	-
NM_018941	CLN8	0.00911788	2.5673356	up	2055	chr8	+
NM_022482	GZF1	0.00320489	2.5638072	up	64412	chr20	+
NM_015658	NOC2L	4.67E-05	2.563702	up	26155	chr1	-
NM_001136203	CCDC124	0.00883633	2.5632718	up	115098	chr19	+
NM_032750	ABHD14B	0.0010289	2.5604758	up	84836	chr3	-
NM_030974	SHARPIN	0.00290762	2.5589345	up	81858	chr8	-
NM_002503	NFKBIB	4.67E-05	2.557798	up	4793	chr19	+
NM_018462	C3orf10	2.28E-04	2.55759	up	55845	chr3	+
NM_003087	SNCG	0.00131531	2.5570354	up	6623	chr10	+
NM_015989	CSAD	0.01661804	2.5568986	up	51380	chr12	-
NM_005505	SCARB1	4.40E-04	2.5563931	up	949	chr12	-
NM_000485	APRT	1.63E-04	2.551688	up	353	chr16	-
NM_003549	HYAL3	0.03379302	2.550746	up	8372	chr3	-
NM_213720	C22orf16	0.00712906	2.550381	up	400916	chr22	-
NM_001312	CRIP2	0.00120394	2.5490031	up	1397	chr14	+
NM_007311	TSPO	0.01050889	2.5489535	up	706	chr22	+
NM_021102	SPINT2	0.0021486	2.5447242	up	10653	chr19	+
NM_014550	CARD10	0.03069731	2.54387	up	29775	chr22	-
NM_014678	SAPS2	0.00139035	2.5438263	up	9701	chr22	+
NM_001037633	SIL1	0.00765943	2.5415974	up	64374	chr5	-
NM_130830	LRRC15	0.00167301	2.539975	up	131578	chr3	-
NM_002754	MAPK13	0.00333987	2.5381975	up	5603	chr6	+
NM_031209	QTRT1	0.00182707	2.537099	up	81890	chr19	+
NM_001099661	EIF3CL	0.00569558	2.5367944	up	728689	chr16	-
NM_032356	LSMD1	5.32E-04	2.5329194	up	84316	chr17	-
NM_152573	RASEF	0.00586349	2.524993	up	158158	chr9	-
NM_005175	ATP5G1	0.00259843	2.5230951	up	516	chr17	+
NM_022127	SLC28A3	0.00366291	2.5182996	up	64078	chr9	-
NM_006187	OAS3	0.01209468	2.5162778	up	4940	chr12	+
NM_052868	IGSF8	0.01921162	2.5120938	up	93185	chr1	-
NM_015432	PLEKHG4	0.00237407	2.511681	up	25894	chr16	+
NM_001037808	EIF3C	0.00294129	2.511588	up	8663	chr16	-
NM_181514	MRPL21	6.15E-04	2.5115318	up	219927	chr11	-
NM_000858	GUK1	7.55E-04	2.509638	up	2987	chr1	+
NM_007255	B4GALT7	0.00101501	2.5094724	up	11285	chr5	+
NM_024738	C12orf49	0.00133725	2.5088685	up	79794	chr12	-
NM_003151	STAT4	0.02834276	2.5086603	up	6775	chr2	-

NM_006221	PIN1	0.03222706	2.505953	up	5300	chr19	+
NM_004946	DOCK2	6.48E-04	2.5020144	up	1794	chr5	+
NM_014652	IPO13	0.00824366	2.5013437	up	9670	chr1	+
NM_018198	DNAJC11	0.00152354	2.50058	up	55735	chr1	-
NM_001082538	TCTN1	8.73E-04	2.4981859	up	79600	chr12	+
NM_173564	FLJ37538	0.0106894	2.4949472	up	222950	chr7	+
NM_001145165	DOHH	8.62E-05	2.4943874	up	83475	chr19	-
NM_003172	SURF1	0.00855622	2.490835	up	6834	chr9	-
NM_015973	GAL	9.52E-04	2.488641	up	51083	chr11	+
NM_004186	SEMA3F	0.00885035	2.4838667	up	6405	chr3	+
NM_152395	NUDT16	0.00176812	2.4813132	up	131870	chr3	+
NM_001001975	ATP5D	1.10E-04	2.480731	up	513	chr19	+
NM_001012636	IL32	0.00280577	2.4805589	up	9235	chr16	+
NM_001079810	XIRP2	0.04362667	2.4796078	up	129446	chr2	+
NM_024028	PCYOX1L	0.00370125	2.4786181	up	78991	chr5	+
NM_000804	FOLR3	0.01017695	2.4744484	up	2352	chr11	+
NM_031206	LAS1L	0.00188651	2.4740803	up	81887	chrX	-
NM_015043	TBC1D9B	0.01086899	2.469845	up	23061	chr5	-
NM_006295	VARS	1.17E-05	2.4670906	up	7407	chr6	-
NM_153360	APCDD1L	0.00382655	2.4648361	up	164284	chr20	-
NM_001042428	ZNF205	1.63E-04	2.4604082	up	7755	chr16	+
NM_001040457	RHBDD2	0.00146675	2.4602115	up	57414	chr7	+
NM_004891	MRPL33	0.00626836	2.4566233	up	9553	chr2	+
NM_016035	COQ4	0.00114569	2.4517288	up	51117	chr9	+
NM_020850	RANBP10	0.00935533	2.4442382	up	57610	chr16	-
NM_007076	HYPE	0.00488325	2.4433706	up	11153	chr12	+
NM_017870	TMEM132A	0.00887076	2.443104	up	54972	chr11	+
NM_006332	IFI30	5.04E-04	2.4416027	up	10437	chr19	+
NM_005481	THRAP5	8.66E-04	2.440165	up	10025	chr19	-
NM_001014764	TMEM93	0.0098424	2.437439	up	83460	chr17	+
NM_004732	KCNAB3	0.00789385	2.435462	up	9196	chr17	-
NM_004793	LONP1	1.54E-04	2.4346097	up	9361	chr19	-
NM_001080543	C19orf29	2.93E-04	2.4336507	up	58509	chr19	-
NM_145283	C9orf121	0.01420071	2.4331398	up	158046	chr9	+
NM_172231	SF4	0.00230088	2.4318976	up	57794	chr19	-
NM_032038	SPNS1	2.08E-04	2.431795	up	83985	chr16	+
NM_181305	MRPL52	0.00663352	2.4317772	up	122704	chr14	+
NM_032377	ELOF1	0.00103503	2.4276078	up	84337	chr19	-
NM_020243	TOMM22	0.00876252	2.4207747	up	56993	chr22	+
NM_006362	NXF1	0.01120015	2.419039	up	10482	chr11	-
NM_144606	FLCN	0.01389377	2.4177954	up	201163	chr17	-
NM_138639	BCL2L12	0.01516311	2.4165874	up	83596	chr19	+
NM_030582	COL18A1	4.08E-04	2.4147623	up	80781	chr21	+
NM_012469	PRPF6	4.00E-04	2.4135525	up	24148	chr20	+
NM_001010982	AFMID	7.36E-05	2.4134696	up	125061	chr17	+
NM_018663	PXMP2	0.0163181	2.4127064	up	5827	chr12	+
NM_207013	TCEB2	2.20E-04	2.4126847	up	6923	chr16	-
NM_001398	ECH1	6.57E-04	2.4126832	up	1891	chr19	-
NM_080748	C20orf52	0.0104214	2.4120708	up	140823	chr20	+

NM_001037984	MGC15523	0.00483745	2.4118938	up	124565	chr17	-
NM_004319	ASTN1	0.00241377	2.4108903	up	460	chr1	-
NM_001082537	TCTN1	0.00216065	2.408376	up	79600	chr12	+
NM_001070	TUBG1	1.43E-05	2.4067624	up	7283	chr17	+
NM_014624	S100A6	0.00543741	2.406464	up	6277	chr1	-
NM_015907	LAP3	4.32E-04	2.4039764	up	51056	chr4	+
NM_017854	TMEM160	0.00611823	2.4018607	up	54958	chr19	-
NM_032339	C17orf37	4.59E-04	2.4002197	up	84299	chr17	-
NM_004819	SYMPK	0.0082543	2.3997025	up	8189	chr19	-
NM_174894	TRAPPC5	0.00666201	2.3996248	up	126003	chr19	+
NM_024316	LENG1	0.00365677	2.3991807	up	79165	chr19	-
NM_001030018	APRT	7.90E-04	2.3987637	up	353	chr16	-
NM_024555	FBXL6	0.00629332	2.3985639	up	26233	chr8	-
NM_015117	ZC3H3	0.00101666	2.395816	up	23144	chr8	-
NM_033058	TRIM55	0.00443749	2.3953924	up	84675	chr8	+
NM_016437	TUBG2	0.00920054	2.3946981	up	27175	chr17	+
NM_024292	UBL5	0.0016661	2.3918679	up	59286	chr19	+
NM_198471	ANKRD47	0.00519675	2.3885925	up	256949	chr19	-
NM_017728	TMEM104	1.07E-04	2.3868642	up	54868	chr17	+
NM_001862	COX5B	5.27E-04	2.3849857	up	1329	chr2	+
NM_133499	SYN1	0.00890824	2.3840983	up	6853	chrX	-
NM_178865	SERINC2	6.91E-04	2.3840525	up	347735	chr1	+
NM_001001502	SNCB	0.00566805	2.382052	up	6620	chr5	-
NM_018992	KCTD5	0.00408533	2.3813515	up	54442	chr16	+
NM_001229	CASP9	1.81E-04	2.3813505	up	842	chr1	-
NM_001009991	SYTL3	0.0387717	2.3806999	up	94120	chr6	+
NM_001039140	C20orf27	0.0019725	2.3761764	up	54976	chr20	-
NM_181304	MRPL52	3.99E-04	2.374214	up	122704	chr14	+
NM_144671	FAM109A	0.01000079	2.373383	up	144717	chr12	-
NM_024042	METRN	3.06E-04	2.3733053	up	79006	chr16	+
NM_032223	PCNXL3	1.35E-04	2.3711631	up	399909	chr11	+
NM_025161	C17orf70	0.00483465	2.3701885	up	80233	chr17	-
NM_134270	SMTN	0.00127908	2.3699064	up	6525	chr22	+
NM_147162	IL11RA	0.00426246	2.369717	up	3590	chr9	+
NM_001031693	HHLA3	0.00411078	2.3692455	up	11147	chr1	+
NM_181515	MRPL21	4.71E-04	2.3686614	up	219927	chr11	-
NM_000487	ARSA	0.04379324	2.3665845	up	410	chr22	-
NM_016292	TRAP1	0.0039489	2.365027	up	10131	chr16	-
NM_002975	CLEC11A	0.00313846	2.3639953	up	6320	chr19	+
NM_017503	SURF2	0.02252529	2.3608797	up	6835	chr9	+
NM_058177	HDAC9	0.01052352	2.357208	up	9734	chr7	+
NM_001145153	TMEM71	0.02682552	2.3546798	up	137835	chr8	-
NM_022135	POPDC2	0.03909033	2.3545709	up	64091	chr3	-
NM_014222	NDUFA8	0.00346971	2.3534691	up	4702	chr9	-
NM_018145	FAM82C	7.17E-06	2.35299	up	55177	chr15	-
NM_012225	NUBP2	0.00154162	2.345483	up	10101	chr16	+
NM_015871	ZNF593	0.00717268	2.3451731	up	51042	chr1	+
NM_020410	ATP13A1	0.00219357	2.3428767	up	57130	chr19	-
NM_001142864	FAM38A	9.33E-04	2.3427358	up	9780	chr16	-

NM_198949	NUDT1	0.00154865	2.341542	up	4521	chr7	+
NM_017836	SLC41A3	6.88E-04	2.3413253	up	54946	chr3	-
NM_138334	JOSD2	0.00265269	2.3398554	up	126119	chr19	-
NM_148920	PIGQ	0.03136537	2.3398054	up	9091	chr16	+
NM_001025235	TSPAN4	0.00323897	2.339367	up	7106	chr11	+
NM_024040	CUEDC2	1.12E-04	2.3382568	up	79004	chr10	-
NM_003278	CLEC3B	0.00800121	2.3380296	up	7123	chr3	+
NM_013370	OSGIN1	8.64E-04	2.3355541	up	29948	chr16	+
NM_006808	SEC61B	1.36E-04	2.3349297	up	10952	chr9	+
NM_000433	NCF2	0.01849582	2.3348649	up	4688	chr1	-
NM_198954	NUDT1	0.00229294	2.3322918	up	4521	chr7	+
NM_001127	AP1B1	0.0027788	2.331222	up	162	chr22	-
NM_001037126	EXOC4	0.002458	2.3310096	up	60412	chr7	+
NM_006715	MAN2C1	0.0012542	2.3303747	up	4123	chr15	-
NM_176677	FLJ36208	0.01056817	2.3300252	up	283948	chr16	+
NM_012320	LYPLA3	0.00356916	2.3270943	up	23659	chr16	+
NM_017722	TRMT1	0.00536996	2.3264775	up	55621	chr19	-
NM_001141936	C4orf48	0.00439623	2.321161	up	401115	chr4	+
NM_198536	UNQ501	0.00150387	2.3198986	up	374882	chr19	-
NM_006690	MMP24	0.00894303	2.3189847	up	10893	chr20	+
NM_001654	ARAF	0.00101588	2.3173773	up	369	chrX	+
NM_057174	PEX16	0.01464375	2.3119822	up	9409	chr11	-
NM_003313	TSTA3	0.00418688	2.3106055	up	7264	chr8	-
NM_001077262	UBXN11	0.04666573	2.309703	up	91544	chr1	-
NM_005529	HSPG2	0.00174781	2.308778	up	3339	chr1	-
NM_022078	GPATCH3	0.00573853	2.3074155	up	63906	chr1	-
NM_001007278	TRIM13	0.00611747	2.3061728	up	10206	chr13	+
NM_005313	PDIA3	0.0088073	2.304303	up	2923	chr15	+
NM_003166	SULT1A3	0.00428558	2.304096	up	6818	chr16	+
NM_004788	UBE4A	7.08E-04	2.3023725	up	9354	chr11	+
NM_033554	HLA-DPA1	0.00891668	2.3005004	up	3113	chr6	-
NM_004127	GPS1	0.01878115	2.2994611	up	2873	chr17	+
NM_201595	GTF2A1	0.02668868	2.2981012	up	2957	chr14	-
NM_001136501	ZNF844	0.01141069	2.297053	up	284391	chr19	+
NM_016134	PGCP	0.02369588	2.295995	up	10404	chr8	+
NM_001039178	ATP5J2	0.01740387	2.29525	up	9551	chr7	-
NM_003562	SLC25A11	0.00532917	2.2940714	up	8402	chr17	-
NM_001011668	CHCHD7	0.00183148	2.2919402	up	79145	chr8	+
NM_001032731	OAS2	0.03414135	2.2917457	up	4939	chr12	+
NM_006280	SSR4	0.02694721	2.2881725	up	6748	chrX	+
NM_002493	NDUFB6	0.01289488	2.2855434	up	4712	chr9	-
NM_001001561	GGA1	0.0123514	2.2849424	up	26088	chr22	+
NM_032829	C12orf34	0.03842977	2.2849128	up	84915	chr12	+
NM_080875	MIB2	0.00270576	2.284832	up	142678	chr1	+
NM_000147	FUCA1	0.00439697	2.2816398	up	2517	chr1	-
NM_001127229	AURKAIP1	6.18E-04	2.2806206	up	54998	chr1	-
NM_033200	TMEM112B	3.54E-05	2.2792158	up	91289	chr22	-
NM_005797	EVA1	0.04958678	2.2789824	up	10205	chr11	-
NM_015956	MRPL4	0.00236414	2.2787445	up	51073	chr19	+

NM_018447	TMEM111	0.00414471	2.278083	up	55831	chr3	-
NM_000532	PCCB	5.54E-04	2.270302	up	5096	chr3	+
NM_021806	FAM3A	8.50E-04	2.2699823	up	60343	chrX	-
NM_004556	NFKBIE	7.74E-04	2.2697918	up	4794	chr6	-
NM_001009921	VPS8	0.0030885	2.2690132	up	23355	chr3	+
NM_003443	ZBTB17	0.00140983	2.2688212	up	7709	chr1	-
NM_031287	SF3B5	0.00458427	2.2685602	up	83443	chr6	-
NM_001076552	ACSS2	0.00302426	2.2679486	up	55902	chr20	+
NM_144598	LRRC28	0.00131903	2.2678661	up	123355	chr15	+
NM_032843	FIBCD1	0.01245572	2.267235	up	84929	chr9	-
NM_002360	MAFK	0.04235547	2.2657802	up	7975	chr7	+
NM_005357	LIPE	6.70E-04	2.265676	up	3991	chr19	-
NM_178449	TIP39	0.04380433	2.2642987	up	113091	chr19	-
NM_152832	FAM89B	0.04759594	2.2640884	up	23625	chr11	+
NM_001251	CD68	0.00640295	2.2639506	up	968	chr17	+
NM_004366	CLCN2	0.02562174	2.2636979	up	1181	chr3	-
NM_021732	AVPI1	0.00498034	2.263608	up	60370	chr10	-
NM_182480	COQ6	0.00576621	2.2624009	up	51004	chr14	+
NM_033504	TMEM54	0.0161261	2.2611628	up	113452	chr1	-
NM_198850	PHLDB3	0.00771327	2.2610822	up	653583	chr19	-
NM_001136537	LOC149478	9.02E-04	2.2591176	up	149478	chr1	+
NM_016466	ANKRD39	7.46E-04	2.258568	up	51239	chr2	-
NM_024531	GPR172A	9.53E-04	2.2565415	up	79581	chr8	+
NM_012227	GTPBP6	0.00442557	2.2560449	up	8225	chrX	-
NM_005931	MICB	6.83E-04	2.2559493	up	4277	chr6_qbl_hap2	+
NM_001024212	S100A13	0.00802623	2.2544913	up	6284	chr1	-
NM_006680	ME3	0.01009977	2.2526903	up	10873	chr11	-
NM_001548	IFIT1	0.0119452	2.2490404	up	3434	chr10	+
NM_018207	TRIM62	0.04950671	2.2477973	up	55223	chr1	-
NM_015969	MRPS17	0.00195656	2.2452452	up	51373	chr7	+
NM_017607	PPP1R12C	0.00448042	2.2451768	up	54776	chr19	-
NM_004220	ZNF213	0.01405225	2.243671	up	7760	chr16	+
NM_001136538	ACAD10	0.02344596	2.243103	up	80724	chr12	+
NM_006509	RELB	0.03264803	2.2427604	up	5971	chr19	+
NM_030821	PLA2G12A	0.02972399	2.2411792	up	81579	chr4	-
NM_004636	SEMA3B	0.00176385	2.2409008	up	7869	chr3	+
NM_006383	CIB2	1.82E-04	2.2405422	up	10518	chr15	-
NM_001129981	ANKRD2	0.01162211	2.2386346	up	26287	chr10	+
NM_022044	SDF2L1	0.02793079	2.2381516	up	23753	chr22	+
NM_001002027	ATP5G1	1.27E-04	2.238134	up	516	chr17	+
NM_175039	ST6GALNAC	0.01594614	2.238048	up	27090	chr9	-
NM_002448	MSX1	0.01091156	2.2358153	up	4487	chr4	+
NM_006455	SC65	2.39E-04	2.235227	up	10609	chr17	-
NM_003365	UQCRC1	0.00213967	2.2339656	up	7384	chr3	-
NM_181755	HSD11B1	0.01686467	2.2336178	up	3290	chr1	+
NM_005094	SLC27A4	0.00131783	2.2332082	up	10999	chr9	+
NM_172087	TNFSF13	5.64E-04	2.2330916	up	8741	chr17	+
NM_005740	DNAL4	0.01968217	2.231486	up	10126	chr22	-
NM_004317	ASNA1	0.00253152	2.23116	up	439	chr19	+

NM_172251	MRPL54	0.00224333	2.229684	up	116541	chr19	+
NM_001144927	NKIRAS2	0.00330924	2.229301	up	28511	chr17	+
NM_020246	SLC12A9	0.01547614	2.2291448	up	56996	chr7	+
NM_018074	CCDC94	0.0417297	2.2287278	up	55702	chr19	+
NM_002846	PTPRN	0.02632093	2.2262871	up	5798	chr2	-
NM_001135642	INPP5K	0.01375411	2.2254815	up	51763	chr17	-
NM_000287	PEX6	0.00498247	2.2253442	up	5190	chr6	-
NM_012392	PEF1	2.72E-04	2.2250447	up	553115	chr1	-
NM_019037	EXOSC4	0.0012235	2.223839	up	54512	chr8	+
NM_006992	LRRC23	0.00175941	2.2232232	up	10233	chr12	+
NM_001136265	IFFO2	0.00315027	2.2215233	up	126917	chr1	-
NM_018891	LAMC2	0.01986164	2.2198148	up	3918	chr1	+
NM_007071	HHLA3	0.00134757	2.2194302	up	11147	chr1	+
NM_001040099	MOSPD3	0.00242332	2.219175	up	64598	chr7	+
NM_006932	SMTN	3.44E-04	2.218619	up	6525	chr22	+
NM_001012762	LOC348180	0.00330822	2.218141	up	348180	chr16	+
NM_198883	MTX1	0.00146227	2.217644	up	4580	chr1	+
NM_033247	PML	0.01949685	2.2159104	up	5371	chr15	+
NM_002461	MVD	0.00360229	2.213587	up	4597	chr16	-
NM_001008528	MXRA7	0.00648873	2.2127619	up	439921	chr17	-
NM_152482	C19orf25	0.00614038	2.2102914	up	148223	chr19	-
NM_006389	HYOU1	0.01686249	2.2088907	up	10525	chr11	-
NM_174905	FAM98C	0.00689878	2.2078278	up	147965	chr19	+
NM_032127	C11orf56	0.03945959	2.2074566	up	84067	chr11	-
NM_022168	IFIH1	0.02776488	2.2070632	up	64135	chr2	-
NM_001077399	PNKD	0.00303359	2.2053628	up	25953	chr2	+
NM_152850	PIGO	0.03348959	2.205322	up	84720	chr9	-
NM_004632	DAP3	0.00143655	2.2027402	up	7818	chr1	+
NM_023948	MOSPD3	0.00224767	2.2021158	up	64598	chr7	+
NM_006443	C6orf108	0.00286372	2.201362	up	10591	chr6	-
NM_018476	BEX1	0.02997475	2.199753	up	55859	chrX	-
NM_205767	P117	0.00237671	2.197249	up	125988	chr19	-
NM_001321	CSRP2	0.02040887	2.1966984	up	1466	chr12	-
NM_001004128	QSOX1	0.01226827	2.196456	up	5768	chr1	+
NM_014203	AP2A1	0.00828396	2.1960626	up	160	chr19	+
NM_015194	MYO1D	0.00309649	2.1953993	up	4642	chr17	-
NM_020761	KIAA1303	0.00888979	2.1951497	up	57521	chr17	+
NM_032928	TMEM141	7.53E-04	2.1940007	up	85014	chr9	+
NM_001355	DDT	0.00376155	2.1929553	up	1652	chr22	-
NM_012410	SEZ6L2	0.00170102	2.1910338	up	26470	chr16	-
NM_058164	OLFM2	0.04104616	2.1897843	up	93145	chr19	-
NM_001001716	NFKBIB	0.02417495	2.1887467	up	4793	chr19	+
NM_006569	CGREF1	0.00233425	2.1885486	up	10669	chr2	-
NM_013445	GAD1	0.02751117	2.1871717	up	2571	chr2	+
NM_173659	RPUSD3	0.01596922	2.18567	up	285367	chr3	-
NM_025008	ADAMTSL4	0.00391505	2.1847925	up	54507	chr1	+
NM_006404	PROCR	0.03948564	2.1844494	up	10544	chr20	+
NM_015720	PODXL2	0.00761415	2.1844091	up	50512	chr3	+
NM_001099432	BCAS3	0.00587295	2.1831474	up	54828	chr17	+

NM_147182	KCNIP4	0.04120698	2.1830573	up	80333	chr4	-
NM_015918	POP5	0.00310543	2.1813457	up	51367	chr12	-
NM_012237	SIRT2	5.22E-05	2.1807978	up	22933	chr19	-
NM_001002246	ANAPC11	0.01273564	2.180609	up	51529	chr17	+
NM_000504	F10	0.01647307	2.1805542	up	2159	chr13	+
NM_001537	HSBP1	0.02372681	2.1800222	up	3281	chr16	+
NM_002960	S100A3	0.00558655	2.179446	up	6274	chr1	-
NM_007065	CDC37	3.33E-04	2.1793408	up	11140	chr19	-
NM_020145	SH3GLB2	0.0091668	2.1775465	up	56904	chr9	-
NM_001025	RPS23	0.00124974	2.1766744	up	6228	chr5	-
NM_002539	ODC1	1.68E-04	2.176509	up	4953	chr2	-
NM_014583	LMCD1	1.94E-04	2.1762097	up	29995	chr3	+
NM_022089	ATP13A2	0.02877291	2.1755755	up	23400	chr1	-
NM_198282	TMEM173	0.00646454	2.1744986	up	340061	chr5	-
NM_201265	UBL7	8.80E-04	2.1727715	up	84993	chr15	-
NM_182491	ZFAND2A	0.00375519	2.1702406	up	90637	chr7	-
NM_002756	MAP2K3	0.00727104	2.1696541	up	5606	chr17	+
NM_052848	CCDC97	0.00231394	2.1692567	up	90324	chr19	+
NM_017617	NOTCH1	7.49E-05	2.1666272	up	4851	chr9	-
NM_032477	MRPL41	0.00595181	2.1656349	up	64975	chr9	+
NM_020745	AARS2	0.00207762	2.1643724	up	57505	chr6	-
NM_001002244	ANAPC11	0.01005343	2.1638353	up	51529	chr17_random	+
NM_016174	CEECAM1	0.0299689	2.1622288	up	51148	chr9	+
NM_001135054	SIGIRR	0.01051144	2.1620603	up	59307	chr11	-
NM_007121	NR1H2	0.03243291	2.1618116	up	7376	chr19	+
NM_024108	TRAPPC6A	0.00980455	2.1616366	up	79090	chr19	-
NM_001040165	C16orf13	0.0231184	2.1603572	up	84326	chr16	-
NM_003883	HDAC3	0.01632353	2.1600282	up	8841	chr5	-
NM_003793	CTSF	0.00812115	2.1561573	up	8722	chr11	-
NM_004567	PFKFB4	0.00556885	2.1555922	up	5210	chr3	-
NM_001202	BMP4	0.0019831	2.1550713	up	652	chr14	-
NM_033508	GCK	0.00536291	2.1546333	up	2645	chr7	-
NM_001039367	ATP6V1E1	0.00185612	2.1545358	up	529	chr22	-
NM_004135	IDH3G	0.00521474	2.1540458	up	3421	chrX	-
NM_001040455	SIDT2	0.0296322	2.152887	up	51092	chr11	+
NM_002705	PPL	0.04194187	2.1504087	up	5493	chr16	-
NM_006019	TCIRG1	4.56E-05	2.1493506	up	10312	chr11	+
NM_002826	QSOX1	0.01047837	2.148088	up	5768	chr1	+
NM_021729	VPS11	0.00474491	2.14664	up	55823	chr11	+
NM_014681	DHX34	0.02079238	2.1430402	up	9704	chr19	+
NM_052850	GADD45GIP1	0.00859346	2.1425643	up	90480	chr19	-
NM_201436	H2AFV	0.01860466	2.141791	up	94239	chr7	-
NM_015016	MAST3	0.00751264	2.139754	up	23031	chr19	+
NM_023933	C16orf24	1.70E-04	2.1390378	up	65990	chr16	+
NM_006844	ILVBL	0.00543112	2.1381006	up	10994	chr19	-
NM_006528	TFPI2	0.02007425	2.1360593	up	7980	chr7	-
NM_000941	POR	2.18E-04	2.135709	up	5447	chr7	+
NM_005857	ZMPSTE24	0.00134758	2.1346998	up	10269	chr1	+
NM_002872	RAC2	0.00214742	2.1334724	up	5880	chr22	-

NM_007175	ERLIN2	0.00134102	2.1331637	up	11160	chr8	+
NM_199336	FAHD2B	0.00490084	2.1311934	up	151313	chr2	-
NM_001007531	C6orf194	0.0060477	2.1302524	up	222698	chr6	+
NM_201589	MAFA	0.00468794	2.1300166	up	389692	chr8	-
NM_001082959	SCARB1	0.0233898	2.1276138	up	949	chr12	-
NM_000263	NAGLU	8.21E-04	2.127476	up	4669	chr17	+
NM_004704	RRP9	0.00133532	2.125848	up	9136	chr3	-
NM_003165	STXBP1	0.04415603	2.125359	up	6812	chr9	+
NM_005110	GFPT2	0.0027385	2.1246538	up	9945	chr5	-
NM_013330	NME7	0.02190535	2.12463	up	29922	chr1	-
NM_199444	COPE	9.49E-05	2.1241314	up	11316	chr19	-
NM_005273	GNB2	0.00115206	2.123887	up	2783	chr7	+
NM_001008485	SLC41A3	0.00565998	2.1228344	up	54946	chr3	-
NM_020451	SEPN1	3.99E-04	2.1224682	up	57190	chr1	+
NM_030622	CYP2S1	1.17E-04	2.1213322	up	29785	chr19	+
NM_001042472	ABHD12	0.02557985	2.1183276	up	26090	chr20	-
NM_000229	LCAT	0.00424521	2.117114	up	3931	chr16	-
NM_001130969	NELF	0.01089236	2.116841	up	26012	chr9	-
NM_018135	MRPS18A	9.50E-05	2.1166537	up	55168	chr6	-
NM_004886	APBA3	0.00102636	2.1162548	up	9546	chr19	-
NM_003491	ARD1A	7.51E-04	2.1158297	up	8260	chrX	-
NM_001130054	EEF1D	0.01463216	2.1152744	up	1936	chr8	-
NM_020470	YIF1A	0.00785281	2.114588	up	10897	chr11	-
NM_147781	CTSB	2.28E-04	2.1140177	up	1508	chr8	-
NM_025245	PBX4	0.04629785	2.1132417	up	80714	chr19	-
NM_006044	HDAC6	0.00624575	2.1123123	up	10013	chrX	+
NM_001001701	LOC401152	0.02299979	2.1116588	up	401152	chr4	-
NM_013241	FHOD1	0.00155349	2.111402	up	29109	chr16	-
NM_181738	PRDX2	0.00429714	2.1109707	up	7001	chr19	-
NM_001360	DHCR7	0.00367554	2.1103988	up	1717	chr11	-
NM_003897	IER3	0.00101607	2.108802	up	8870	chr6_qbl_hap2	-
NM_145729	MRPL24	9.67E-05	2.1087394	up	79590	chr1	-
NM_001134364	MAP4	0.00526728	2.107414	up	4134	chr3	-
NM_138362	FAM104B	0.00224447	2.1055949	up	90736	chrX	-
NM_014003	DHX38	0.00407338	2.10527	up	9785	chr16	+
NM_020442	VARS2	0.0259758	2.1052272	up	57176	chr6	+
NM_003283	TNNT1	0.0319762	2.1043758	up	7138	chr19	-
NM_000666	ACY1	0.00273703	2.103791	up	95	chr3	+
NM_005452	WDR46	0.00218356	2.1032333	up	9277	chr6	-
NM_016111	TELO2	0.03170927	2.1026342	up	9894	chr16	+
NM_183386	ACOT8	0.02427496	2.1024716	up	10005	chr20	-
NM_145109	MAP2K3	2.73E-04	2.1017365	up	5606	chr17	+
NM_015703	CTA-126B4.3	0.02133915	2.1009893	up	27341	chr22	-
NM_152369	SLC44A3	0.0136778	2.0996146	up	126969	chr1	+
NM_006227	PLTP	0.00471152	2.0993261	up	5360	chr20	-
NM_138392	SHKBP1	0.00150003	2.0982592	up	92799	chr19	+
NM_003009	SEPW1	0.00378265	2.097667	up	6415	chr19	+
NM_004943	DMWD	0.01646937	2.09617	up	1762	chr19	-
NM_016445	PLEK2	0.03032176	2.094703	up	26499	chr14	-

NM_001002233	RAB11FIP1	0.00701697	2.093583	up	80223	chr8	-
NM_025267	AARSD1	0.01042581	2.0922287	up	80755	chr17	-
NM_182915	STEAP3	0.00110799	2.0920572	up	55240	chr2	+
NM_002798	PSMB6	0.00258887	2.0881333	up	5694	chr17	+
NM_005034	POLR2K	8.88E-04	2.0878754	up	5440	chr8	+
NM_175932	PSMD13	0.01342417	2.0868487	up	5719	chr11	+
NM_016146	TRAPPC4	0.02465318	2.0866432	up	51399	chr11	+
NM_016293	BIN2	0.00426892	2.085913	up	51411	chr12	-
NM_007002	ADRM1	0.00531348	2.0847268	up	11047	chr20	+
NM_007017	SOX30	0.04287224	2.083987	up	11063	chr5	-
NM_005700	DPP3	0.00429465	2.0811496	up	10072	chr11	+
NM_182547	TMED4	0.0183401	2.077665	up	222068	chr7	-
NM_175573	ADRM1	0.02101931	2.0754483	up	11047	chr20	+
NM_022092	CHTF18	0.00247502	2.0749462	up	63922	chr16	+
NM_001018139	NME2	0.00602181	2.0745442	up	4831	chr17	+
NM_031210	C14orf156	0.00798241	2.0739985	up	81892	chr14	+
NM_001136202	ISOC2	2.46E-04	2.0739217	up	79763	chr19	-
NM_001005920	LOC339123	0.00192439	2.0734186	up	339123	chr16	-
NM_001249	ENTPD5	0.00983295	2.0723643	up	957	chr14	-
NM_001130955	ARHGEF18	0.04315596	2.0702016	up	23370	chr19	+
NM_001457	FLNB	0.00439888	2.0700908	up	2317	chr3	+
NM_001012456	SEC61G	0.0056711	2.0697925	up	23480	chr7	-
NM_022743	SMYD3	0.00302676	2.0693192	up	64754	chr1	-
NM_000404	GLB1	5.89E-04	2.0689936	up	2720	chr3	-
NM_006730	DNASE1L1	0.00192737	2.0684009	up	1774	chrX	-
NM_016055	MRPL48	3.75E-04	2.0679326	up	51642	chr11	+
NM_024308	MGC4172	0.01757576	2.0672903	up	79154	chr17	+
NM_178150	FBXO18	0.03028558	2.0661125	up	84893	chr10	+
NM_004470	FKBP2	0.00180536	2.065793	up	2286	chr11	+
NM_015407	ABHD14A	0.00892947	2.064499	up	25864	chr3	+
NM_020816	KIF17	0.00983011	2.0643985	up	57576	chr1	-
NM_153281	HYAL1	4.74E-04	2.063683	up	3373	chr3	-
NM_012162	FBXL6	0.00138044	2.0634353	up	26233	chr8	-
NM_014740	EIF4A3	0.00626957	2.061056	up	9775	chr17	-
NM_001005409	SF3A1	0.02170451	2.0606513	up	10291	chr22	-
NM_152743	C7orf27	0.01966328	2.0594623	up	221927	chr7	-
NM_021127	PMAIP1	0.00342028	2.0574458	up	5366	chr18	+
NM_018467	MDS032	0.03226776	2.0566123	up	55850	chr19	+
NM_002150	HPD	0.00125777	2.0557601	up	3242	chr12	-
NM_007173	PRSS23	0.02609389	2.0553422	up	11098	chr11	+
NM_006406	PRDX4	0.00143256	2.0534565	up	10549	chrX	+
NM_007135	ZNF79	0.0084628	2.0521472	up	7633	chr9	+
NM_080750	DPH3B	6.32E-04	2.0494778	up	100132911	chr20	+
NM_182476	COQ6	9.33E-04	2.0477283	up	51004	chr14	+
NM_001018138	NME2	0.00126101	2.0467687	up	4831	chr17	+
NM_175063	LOC284361	0.00236525	2.0464277	up	284361	chr19	+
NM_001126121	SLC25A19	0.01275729	2.043279	up	60386	chr17	-
NM_013291	CPSF1	0.00918688	2.041159	up	29894	chr8	-
NM_016291	IHPK2	0.00521431	2.0408032	up	51447	chr3	-

NM_130851	BMP4	0.01136839	2.0406663	up	652	chr14	-
NM_001916	CYC1	0.00195962	2.0404708	up	1537	chr8	+
NM_181462	MRPL55	0.00793218	2.0398586	up	128308	chr1	-
NM_004313	ARRB2	0.03712227	2.03679	up	409	chr17	+
NM_014520	MYBBP1A	0.00776014	2.034913	up	10514	chr17	-
NM_007263	COPE	0.00517099	2.034554	up	11316	chr19	-
NM_016829	OGG1	0.03223636	2.0330093	up	4968	chr3	+
NM_012470	TNPO3	0.00159333	2.03073	up	23534	chr7	-
NM_006392	NOL5A	0.01847294	2.0291452	up	10528	chr20	+
NM_024053	CENPM	0.00296214	2.028517	up	79019	chr22	-
NM_178122	C1orf201	0.00238626	2.0272226	up	90529	chr1	-
NM_001145526	AFMID	2.82E-04	2.026569	up	125061	chr17	+
NM_001003935	PARP3	0.00155632	2.0242805	up	10039	chr3	+
NM_017528	WBSCR22	0.011265	2.023351	up	114049	chr7	+
NM_198690	KRTAP10-9	0.00467772	2.023096	up	386676	chr21	+
NM_001901	CTGF	0.03980986	2.0230432	up	1490	chr6	-
NM_007100	ATP5I	0.03017756	2.0224016	up	521	chr4	-
NM_199173	BGLAP	0.03630505	2.0216074	up	632	chr1	+
NM_003355	UCP2	9.55E-04	2.0207899	up	7351	chr11	-
NM_005567	LGALS3BP	9.32E-04	2.019281	up	3959	chr17	-
NM_014984	AZI1	0.01476234	2.0192301	up	22994	chr17	-
NM_016256	NAGPA	0.01420351	2.0188272	up	51172	chr16	-
NM_018170	P15RS	0.00676879	2.018144	up	55197	chr18	-
NM_213604	ADAMTSL5	0.01133236	2.0179012	up	339366	chr19	-
NM_001001479	SLC35E4	0.01201292	2.0170398	up	339665	chr22	+
NM_001860	SLC31A2	0.00900476	2.0163825	up	1318	chr9	+
NM_138425	C12orf57	0.00205295	2.0159013	up	113246	chr12	+
NM_197964	HSPC268	0.01546283	2.0142841	up	154791	chr7	+
NM_019896	POLE4	0.00118345	2.0141137	up	56655	chr2	+
NM_001136050	DHRS1	0.00671191	2.013829	up	115817	chr14	-
NM_138374	ZNF845	0.02309608	2.0129225	up	91664	chr19	+
NM_002337	LRPAP1	1.97E-04	2.0125952	up	4043	chr4	-
NM_207122	EXT2	0.0041722	2.0125074	up	2132	chr11	+
NM_001084392	DDT	0.03523771	2.0120125	up	1652	chr22	-
NM_001127257	SLC39A10	0.00701849	2.0116735	up	57181	chr2	+
NM_005528	DNAJC4	0.01354649	2.0113106	up	3338	chr11	+
NM_052873	C14orf179	0.0199675	2.010727	up	112752	chr14	+
NM_001098540	HPSE	0.0039863	2.0103354	up	10855	chr4	-
NM_014017	MAPBPIP	7.68E-04	2.0095842	up	28956	chr1	+
NM_015388	YIPF3	3.44E-04	2.0095842	up	25844	chr6	-
NM_003062	SLIT3	0.01485245	2.0090516	up	6586	chr5	-
NM_024307	GDPD3	0.01252367	2.0084221	up	79153	chr16	-
NM_024779	PIP5K2C	0.00130464	2.0062313	up	79837	chr12	+
NM_000821	GGCX	0.03202755	2.0058575	up	2677	chr2	-
NM_018161	NADSYN1	7.93E-04	2.0057435	up	55191	chr11	+
NM_183057	VPS28	0.0033295	2.005449	up	51160	chr8	-
NM_006899	IDH3B	0.00202703	2.0046484	up	3420	chr20	-
NM_203473	PORCN	0.00176362	2.0044127	up	64840	chrX	+
NM_001014979	LOC90835	0.04582936	2.0038874	up	90835	chr16	-

NM_001410	MEGF8	0.0347033	2.0035436	up	1954	chr19	+
NM_001036646	HHLA3	0.0026256	2.0014257	up	11147	chr1	+
NM_014297	ETHE1	7.28E-05	2.000767	up	23474	chr19	-

SEQ_ID	GeneSymbol	p-value	FCAbsolute	regulation	EntrezGene	Chromosome	Strand
NM_006206	PDGFRA	4.55E-05	15.999931	down	5156	chr4	+
NM_005584	MAB21L1	9.27E-04	14.712783	down	4081	chr13	-
NM_181486	TBX5	1.04E-04	12.980625	down	6910	chr12	-
NM_005392	PHF2	0.001262	12.953551	down	5253	chr9	+
NM_014212	HOXC11	4.86E-05	12.8718605	down	3227	chr12	+
NM_000192	TBX5	3.41E-05	12.485831	down	6910	chr12	-
NM_170675	MEIS2	0.00167138	12.320673	down	4212	chr15	-
NM_002221	ITPKB	3.88E-04	12.316081	down	3707	chr1	-
NM_173576	MKX	0.00101995	11.540696	down	283078	chr10	-
NM_005786	TSHZ1	2.48E-04	10.631396	down	10194	chr18	+
NM_006883	SHOX	0.00205806	10.298605	down	6473	chrY	+
NM_002833	PTPN9	4.23E-04	10.066915	down	5780	chr15	-
NM_153000	APCDD1	0.00617118	9.64571	down	147495	chr18	+
NM_005157	ABL1	2.68E-04	9.640917	down	25	chr9	+
NM_080718	TBX5	1.72E-05	9.630111	down	6910	chr12	-
NM_014782	ARMCX2	0.00501837	9.282509	down	9823	chrX	-
NM_001343	DAB2	6.85E-04	9.216502	down	1601	chr5	-
NM_033260	FOXQ1	2.05E-05	9.141313	down	94234	chr6	+
NM_170677	MEIS2	0.0011767	9.028622	down	4212	chr15	-
NM_152857	WTAP	0.01502881	8.916066	down	9589	chr6	+
NM_015864	C6orf32	0.00130223	8.746736	down	9750	chr6	-
NM_175907	ZADH2	0.00209806	8.681764	down	284273	chr18	-
NM_000609	CXCL12	0.00203383	8.577887	down	6387	chr10	-
NM_001452	FOXF2	0.00437267	8.443264	down	2295	chr6	+
NM_001142462	OSR2	0.00116572	8.361232	down	116039	chr8	+
NM_003362	UNG	0.00401872	8.359181	down	7374	chr12	+
NM_004349	RUNX1T1	3.49E-05	8.309246	down	862	chr8	-
NM_020940	KIAA1600	1.59E-04	8.304444	down	57700	chr10	+
NM_003714	STC2	4.85E-04	8.169268	down	8614	chr5	-
NM_018689	KIAA1199	4.06E-04	8.121161	down	57214	chr15	+
NM_004282	BAG2	0.01136142	7.96778	down	9532	chr6	+
NM_177949	ARMCX2	0.00243099	7.744183	down	9823	chrX	-
NM_001079539	XBP1	0.00201321	7.7046924	down	7494	chr22	-
NM_004560	ROR2	0.00137653	7.6769366	down	4920	chr9	-
NM_053001	OSR2	7.59E-04	7.673738	down	116039	chr8	+
NM_012098	ANGPTL2	0.00717347	7.5692844	down	23452	chr9	-
NM_017519	ARID1B	0.00396631	7.5501375	down	57492	chr6	+
NM_030674	SLC38A1	0.01487794	7.539512	down	81539	chr12	-
NM_000522	HOXA13	1.62E-04	7.45821	down	3209	chr7	-
NM_006618	JARID1B	0.00173303	7.344133	down	10765	chr1	-
NM_001098812	SEPT8	4.02E-04	7.187704	down	23176	chr5	-
NM_016073	HDGFRP3	0.00659646	7.1511574	down	50810	chr15	-
NM_014899	RHOBTB3	0.00732655	7.126093	down	22836	chr5	+

NM_016604	JMJD1B	0.00129196	7.048702	down	51780 chr5	+
NM_005349	RBPJ	1.71E-04	6.9808946	down	3516 chr4	+
NM_005654	NR2F1	0.00116909	6.8620234	down	7025 chr5	+
NM_207036	TCF12	4.36E-04	6.7799106	down	6938 chr15	+
NM_001141979	TP53BP1	7.04E-05	6.6959686	down	7158 chr15	-
NM_018660	ZNF395	0.00468371	6.6391387	down	55893 chr8	-
NM_173469	UBE2Q2	4.54E-05	6.6326423	down	92912 chr15	+
NM_006265	RAD21	0.00108804	6.6275473	down	5885 chr8	-
NM_021623	PLEKHA2	7.79E-04	6.596968	down	59339 chr8	+
NM_001037340	PDE4B	1.87E-04	6.5181627	down	5142 chr1	+
NM_080911	UNG	0.01367755	6.4755855	down	7374 chr12	+
NM_057158	DUSP4	6.29E-04	6.261241	down	1846 chr8	-
NM_152493	FLJ25476	2.15E-04	6.2489977	down	149076 chr1	+
NM_001754	RUNX1	0.00685387	6.248913	down	861 chr21	-
NM_006026	H1FX	0.00466526	6.220482	down	8971 chr3	-
NM_053042	KIAA1729	2.79E-04	6.2191353	down	85460 chr4	-
NM_005195	CEBPD	6.00E-04	6.2039304	down	1052 chr8	-
NM_018475	TMEM165	0.00655132	6.19609	down	55858 chr4	+
NM_012176	FBXO4	0.00325758	6.0876503	down	26272 chr5	+
NM_021973	HAND2	4.00E-04	6.06067	down	9464 chr4	-
NM_001129994	KCTD15	0.00113057	6.0497584	down	79047 chr19	+
NM_000474	TWIST1	0.00230151	6.0224385	down	7291 chr7	-
NM_001136575	LANCL1	0.0031644	5.974221	down	10314 chr2	-
NM_001423	EMP1	0.00766343	5.910105	down	2012 chr12	+
NM_003845	DYRK4	2.00E-04	5.9053564	down	8798 chr12	+
NM_006902	PRRX1	9.94E-04	5.893575	down	5396 chr1	+
NM_001048200	HIPK3	0.00310538	5.8924203	down	10114 chr11	+
NM_001432	EREG	3.04E-04	5.8892016	down	2069 chr4	+
NM_015288	PHF15	7.12E-04	5.872612	down	23338 chr5	+
NM_016569	TBX3	5.76E-04	5.8337383	down	6926 chr12	-
NM_022051	EGLN1	0.00925718	5.757154	down	54583 chr1	-
NM_000623	BDKRB2	0.00808773	5.717361	down	624 chr14	+
NM_001136574	LANCL1	0.00229113	5.7097073	down	10314 chr2	-
NM_001101669	INPP4B	0.00331431	5.704448	down	8821 chr4	-
NM_002546	TNFRSF11B	3.14E-06	5.688826	down	4982 chr8	-
NM_173547	TRIM65	0.0313221	5.6801066	down	201292 chr17	-
NM_006887	ZFP36L2	0.00583725	5.677466	down	678 chr2	-
NM_003031	SIAH1	0.00362848	5.604022	down	6477 chr16	-
NM_015009	PDZRN3	0.00333277	5.582974	down	23024 chr3	-
NM_001077484	SLC38A1	0.00159287	5.578315	down	81539 chr12	-
NM_001012241	MSL1	0.01011978	5.553388	down	339287 chr17	+
NM_013233	STK39	0.00196505	5.5483975	down	27347 chr2	-
NM_001097599	TMEM22	0.01236878	5.547677	down	80723 chr3	+
NM_152278	TCEAL7	0.01831477	5.5227995	down	56849 chrX	+
NM_182948	PRKACB	0.00550134	5.517625	down	5567 chr1	+
NM_021629	GNB4	4.56E-04	5.514848	down	59345 chr3	-
NM_005098	MSC	0.01598607	5.4856377	down	9242 chr8	-
NM_005387	NUP98	0.00208361	5.4756603	down	4928 chr11	-
NM_001135993	TTC39C	7.78E-05	5.4314346	down	125488 chr18	+

NM_178012	TUBB2B	3.13E-04	5.430794	down	347733	chr6	-
NM_001033910	TRAF5	2.80E-04	5.402873	down	7188	chr1	+
NM_001786	CDC2	7.72E-04	5.397313	down	983	chr10	+
NM_003030	SHOX2	7.20E-04	5.373016	down	6474	chr3	-
NM_001135051	FAM160B1	0.00803785	5.370298	down	57700	chr10	+
NM_198240	CLIP1	0.00123363	5.3525896	down	6249	chr12	-
NM_018011	FLJ10154	0.00436956	5.3439794	down	55082	chr13	-
NM_018492	PBK	0.00176647	5.339447	down	55872	chr8	-
NM_001025580	AMMECR1	0.00137687	5.338014	down	9949	chrX	-
NM_016424	CROP	6.37E-05	5.321876	down	51747	chr17	+
NM_001003699	RREB1	0.00832079	5.298596	down	6239	chr6	+
NM_002356	MARCKS	9.60E-04	5.297251	down	4082	chr6	+
NM_080746	RPL10L	0.02227268	5.262586	down	140801	chr14	-
NM_001012761	RGMB	0.00826125	5.2350755	down	285704	chr5	+
NM_145260	OSR1	0.00277954	5.2251773	down	130497	chr2	-
NM_130781	RAB24	0.00134446	5.218855	down	53917	chr5	-
NM_020967	NCOA5	0.01967096	5.2177234	down	57727	chr20	-
NM_001033925	TIAL1	1.53E-04	5.209106	down	7073	chr10	-
NM_198309	TTC8	1.75E-04	5.206273	down	123016	chr14	+
NM_024618	NLRX1	0.001911	5.1846037	down	79671	chr11	+
NM_006714	SMPDL3A	0.0022799	5.132231	down	10924	chr6	+
NM_005096	ZMYM3	0.01474825	5.1254134	down	9203	chrX	-
NM_005095	ZMYM4	6.01E-04	5.1052804	down	9202	chr1	+
NM_001130688	HMGB2	7.70E-04	5.0829124	down	3148	chr4	-
NM_014720	SLK	0.01421946	5.076182	down	9748	chr10	+
NM_004523	KIF11	0.00447393	5.0618105	down	3832	chr10	+
NM_001130689	HMGB2	1.86E-04	5.061431	down	3148	chr4	-
NM_015153	PHF3	1.28E-05	5.050844	down	23469	chr6	+
NM_002514	NOV	0.0088838	5.0332494	down	4856	chr8	+
NM_183373	C6orf145	0.00210972	5.0172925	down	221749	chr6	-
NM_144949	SOCS5	0.00388175	4.9723577	down	9655	chr2	+
NM_014811	KIAA0649	7.11E-04	4.9413342	down	9858	chr9	+
NM_033286	C15orf23	0.0050707	4.935985	down	90417	chr15	+
NM_005631	SMO	0.00450025	4.9074836	down	6608	chr7	+
NM_018488	TBX4	0.00449122	4.903446	down	9496	chr17	+
NM_003686	EXO1	0.01711156	4.8853006	down	9156	chr1	+
NM_199460	CACNA1C	1.15E-04	4.8806124	down	775	chr12	+
NM_001127184	CFLAR	0.00654965	4.8724003	down	8837	chr2	+
NM_006045	ATP9A	1.41E-04	4.8694654	down	10079	chr20	-
NM_152858	WTAP	8.99E-05	4.8427114	down	9589	chr6	+
NM_133375	DIS3L	0.04758224	4.84206	down	115752	chr15	+
NM_001143976	WEE1	0.00101934	4.8411684	down	7465	chr11	+
NM_006055	LANCL1	1.34E-04	4.836818	down	10314	chr2	-
NM_007018	CEP110	0.00345662	4.8262916	down	11064	chr9	+
NM_001401	LPAR1	0.00866858	4.82278	down	1902	chr9	-
NM_133468	BMPER	5.82E-04	4.81338	down	168667	chr7	+
NM_002006	FGF2	5.51E-04	4.810447	down	2247	chr4	+
NM_001946	DUSP6	0.01260494	4.797034	down	1848	chr12	-
NM_005730	CTDSP2	6.60E-04	4.795181	down	10106	chr12	-

NM_032459	EFS	0.00696097	4.777275	down	10278	chr14	-
NM_025246	TMEM22	7.66E-05	4.760331	down	80723	chr3	+
NM_022836	DCLRE1B	6.60E-04	4.7517796	down	64858	chr1	+
NM_005279	GPR1	6.87E-04	4.7215505	down	2825	chr2	-
NM_015516	TSKU	6.53E-04	4.689827	down	25987	chr11	+
NM_032552	DAB2IP	0.00608616	4.6887975	down	153090	chr9	+
NM_018155	SLC25A36	0.00306603	4.6743965	down	55186	chr3	+
NM_024076	KCTD15	1.57E-04	4.6720843	down	79047	chr19	+
NM_018951	HOXA10	5.97E-04	4.6645637	down	3206	chr7	-
NM_003712	PPAP2C	0.02218296	4.649933	down	8612	chr19	-
NM_015146	SEPT8	6.96E-04	4.642215	down	23176	chr5	-
NM_001098624	MID1	0.02378511	4.638034	down	4281	chrX	-
NM_015419	MXRA5	0.00302961	4.636927	down	25878	chrX	-
NM_032883	TOX2	0.01429377	4.6315556	down	84969	chr20	+
NM_001017406	S100PBP	0.01002565	4.5943055	down	64766	chr1	+
NM_014315	KLHDC2	0.00668339	4.5895047	down	23588	chr14	+
NM_018012	KIF26B	0.01767489	4.5849032	down	55083	chr1	+
NM_001237	CCNA2	6.63E-05	4.577481	down	890	chr4	-
NM_005864	EFS	0.0023252	4.57476	down	10278	chr14	-
NM_022337	RAB38	9.78E-04	4.5395174	down	23682	chr11	-
NM_015048	SETD1B	1.08E-04	4.5354247	down	23067	chr12	+
NM_018357	LARP6	5.96E-05	4.5285063	down	55323	chr15	-
NM_014397	NEK6	0.00660058	4.506359	down	10783	chr9	+
NM_032199	ARID5B	7.85E-06	4.504907	down	84159	chr10	+
NM_014766	SCRN1	0.00964057	4.504505	down	9805	chr7	-
NM_015617	PYGO1	0.00361529	4.488564	down	26108	chr15	-
NM_012208	HARS2	0.00560115	4.4809585	down	23438	chr5	+
NM_152739	HOXA9	0.00443327	4.46998	down	3205	chr7	-
NM_001098515	MRGPRF	9.79E-04	4.4551373	down	116535	chr11	-
NM_021158	TRIB3	0.02446686	4.444476	down	57761	chr20	+
NM_007146	VEZF1	0.01723796	4.443352	down	7716	chr17	-
NM_030621	DICER1	0.00801266	4.44217	down	23405	chr14	-
NM_144599	NIPA1	0.00941351	4.442076	down	123606	chr15	-
NM_022347	IFRG15	0.02067666	4.4246097	down	64163	chr1	-
NM_032139	ANKRD27	0.01143313	4.4237385	down	84079	chr19	-
NM_001221	CAMK2D	0.0017936	4.4175563	down	817	chr4	-
NM_006497	HIC1	0.02186371	4.414185	down	3090	chr17	+
NM_019012	PLEKHA5	0.0014871	4.400156	down	54477	chr12	+
NM_178422	PAQR7	0.00558426	4.3893013	down	164091	chr1	-
NM_001032283	TMPO	1.11E-04	4.3892603	down	7112	chr12	+
NM_001145514	SCRN1	6.87E-05	4.3869805	down	9805	chr7	-
NM_152495	CNIH3	0.00443612	4.3686767	down	149111	chr1	+
NM_020744	MTA3	0.00865852	4.367724	down	57504	chr2	+
NM_006591	POLD3	0.00347495	4.3619	down	10714	chr11	+
NM_003861	WDR22	8.81E-04	4.3590546	down	8816	chr14	-
NM_017738	C9orf39	0.00469571	4.3525834	down	54875	chr9	+
NM_145214	TRIM11	9.52E-04	4.3517866	down	81559	chr1	-
NM_198433	AURKA	0.00364742	4.351585	down	6790	chr20	-
NM_007314	ABL2	0.00237695	4.3452425	down	27	chr1	-

NM_194328	RNF38	0.00151738	4.337546	down	152006	chr9	-
NM_005318	H1F0	1.03E-04	4.3331366	down	3005	chr22	+
NM_020824	ARHGAP21	0.02654615	4.331207	down	57584	chr10	-
NM_001010924	C10orf38	0.02851888	4.3164654	down	221061	chr10	-
NM_007294	BRCA1	4.51E-04	4.31375	down	672	chr17	-
NM_016500	CXorf26	1.45E-04	4.313519	down	51260	chrX	+
NM_001122842	NCOA7	3.21E-04	4.3099732	down	135112	chr6	+
NM_022353	OSGEPL1	0.01016034	4.306702	down	64172	chr2	-
NM_001145815	AMDHD2	0.00125823	4.300496	down	51005	chr16	+
NM_001098202	HIC1	0.00206009	4.2963977	down	3090	chr17	+
NM_001042481	FRMD6	0.00102004	4.2925677	down	122786	chr14	+
NM_201629	TJP2	0.04419846	4.2907906	down	9414	chr9	+
NM_001033030	FAIM	0.00192676	4.285528	down	55179	chr3	+
NM_152330	FRMD6	0.00441318	4.2802396	down	122786	chr14	+
NM_018348	FLJ11171	2.61E-04	4.2592263	down	55783	chr16	-
NM_002915	RFC3	5.51E-04	4.2552543	down	5983	chr13	+
NM_004780	TCEAL1	0.00917123	4.2526574	down	9338	chrX	+
NM_007156	ZXDA	1.03E-04	4.2469125	down	7789	chrX	-
NM_080836	STK35	0.00516813	4.2343693	down	140901	chr20	+
NM_031283	TCF7L1	0.00115332	4.231567	down	83439	chr2	+
NM_003557	PIP5K1A	0.0019584	4.2298374	down	8394	chr1	+
NM_012307	EPB41L3	0.01169112	4.2277827	down	23136	chr18	-
NM_001009555	SH3D19	0.00139068	4.220976	down	152503	chr4	-
NM_000963	PTGS2	8.28E-05	4.199105	down	5743	chr1	-
NM_003222	TFAP2C	0.00294905	4.189218	down	7022	chr20	+
NM_015168	C19orf7	0.00255487	4.175911	down	23211	chr19	-
NM_007083	NUDT6	7.06E-04	4.1640687	down	11162	chr4	-
NM_030963	RNF146	0.01388901	4.163593	down	81847	chr6	+
NM_001394	DUSP4	9.20E-04	4.162781	down	1846	chr8	-
NM_006938	SNRPD1	0.00459185	4.146288	down	6632	chr18	+
NM_175921	LOC285636	0.00386544	4.139172	down	285636	chr5	+
NM_022763	FNDC3B	8.48E-04	4.137883	down	64778	chr3	+
NM_001012989	UBE2NL	0.01252124	4.101838	down	389898	chrX	+
NM_001102654	NTF3	0.00267531	4.1014533	down	4908	chr12	+
NM_032501	ACSS1	0.0035874	4.092026	down	84532	chr20	-
NM_006526	ZNF217	2.11E-04	4.0906243	down	7764	chr20	-
NM_001112736	C3orf63	0.01646028	4.0882955	down	23272	chr3	-
NM_001135095	FNDC3B	3.26E-04	4.084477	down	64778	chr3	+
NM_003905	APPBP1	0.01179879	4.07418	down	8883	chr16	-
NM_018101	CDCA8	4.04E-04	4.059055	down	55143	chr1	+
NM_080616	C20orf112	0.00102989	4.0534415	down	140688	chr20	-
NM_001142966	KIAA1772	2.82E-04	4.0492945	down	80000	chr18	+
NM_001098813	SEPT8	0.00422716	4.0474377	down	23176	chr5	-
NM_001099294	KIAA1644	3.41E-04	4.031804	down	85352	chr22	-
NM_002388	MCM3	0.00291928	4.0313125	down	4172	chr6	-
NM_001144891	MBIP	0.00313732	4.023529	down	51562	chr14	-
NM_014812	CEP170	1.01E-04	4.0137134	down	9859	chr1	-
NM_001080383	GJC1	8.08E-04	4.0117173	down	10052	chr17	-
NM_003392	WNT5A	1.43E-05	4.010946	down	7474	chr3	-

NM_001198	PRDM1	9.94E-04	4.0069203	down	639	chr6	+
NM_022549	FEZ1	0.03314605	3.9818456	down	9638	chr11	-
NM_016009	SH3GLB1	7.04E-04	3.9800706	down	51100	chr1	+
NM_199005	ZNF322B	0.00182771	3.9790275	down	387328	chr9	-
NM_007313	ABL1	4.57E-04	3.9756532	down	25	chr9	+
NM_152528	WDSUB1	0.01765597	3.9726083	down	151525	chr2	-
NM_001083908	C14orf104	3.65E-04	3.9690897	down	55172	chr14	-
NM_018064	C6orf166	0.00275165	3.9672391	down	55122	chr6	-
NM_022149	MAGEF1	0.00165739	3.9642086	down	64110	chr3	-
NM_002912	REV3L	0.00857969	3.963906	down	5980	chr6	-
NM_001031723	DNAJB14	0.00565373	3.9619832	down	79982	chr4	-
NM_080683	PTPN13	0.02435526	3.9612577	down	5783	chr4	+
NM_030777	SLC2A10	0.00226686	3.9604082	down	81031	chr20	+
NM_006660	CLPX	0.00499049	3.9600651	down	10845	chr15	-
NM_001134793	HYLS1	0.01246925	3.9562385	down	219844	chr11	+
NM_003589	CUL4A	0.02773846	3.9531748	down	8451	chr13	+
NM_001144772	NSMAF	0.02626631	3.9477704	down	8439	chr8	-
NM_021224	ZNF462	1.12E-04	3.9272861	down	58499	chr9	+
NM_030665	RAI1	0.00537011	3.9270175	down	10743	chr17	+
NM_001007466	TULP4	0.00388	3.9185963	down	56995	chr6	+
NM_032040	CCDC8	0.02571642	3.9090533	down	83987	chr19	-
NM_182697	UBE2H	0.00106454	3.9073212	down	7328	chr7	-
NM_052903	TUBGCP5	0.04151361	3.9032733	down	114791	chr15	+
NM_145687	MAP4K4	0.00284124	3.8964224	down	9448	chr2	+
NM_000165	GJA1	0.00504543	3.8902574	down	2697	chr6	+
NM_020730	DLG3	0.00298913	3.8850815	down	1741	chrX	+
NM_006041	HS3ST3B1	0.00291112	3.8847709	down	9953	chr17	+
NM_016343	CENPF	0.00241262	3.8844986	down	1063	chr1	+
NM_033046	RTKN	0.01388227	3.8842187	down	6242	chr2	-
NM_017925	DENND4C	4.12E-04	3.8838143	down	55667	chr9	+
NM_004856	KIF23	0.00931733	3.871284	down	9493	chr15	+
NM_139045	SMARCA2	0.00954119	3.8647947	down	6595	chr9	+
NM_000527	LDLR	0.03067254	3.8572628	down	3949	chr19	+
NM_017943	FBXO34	0.02910074	3.8564675	down	55030	chr14	+
NM_021229	NTN4	8.38E-04	3.8518994	down	59277	chr12	-
NM_015271	TRIM2	0.00658292	3.8483908	down	23321	chr4	+
NM_001042734	SEC24B	0.00646416	3.845385	down	10427	chr4	+
NM_003489	NRIP1	0.00551908	3.8417506	down	8204	chr21	-
NM_001105570	NUDT19	0.00156693	3.8381956	down	390916	chr19	+
NM_017565	FAM20A	1.80E-04	3.8372214	down	54757	chr17	-
NM_080796	DIDO1	7.29E-05	3.831649	down	11083	chr20	-
NM_016644	PRR16	0.00474069	3.8289347	down	51334	chr5	+
NM_001145335	UBE2Q2	4.40E-04	3.8280022	down	92912	chr15	+
NM_138447	ZNF689	0.01442788	3.82655	down	115509	chr16	-
NM_001011645	AR	0.00357451	3.8143735	down	367	chrX	+
NM_004275	TRFP	3.55E-04	3.7987523	down	9477	chr6	-
NM_018139	C14orf104	7.24E-04	3.7972121	down	55172	chr14	-
NM_001987	ETV6	0.04943506	3.796429	down	2120	chr12	+
NM_022105	DIDO1	8.07E-04	3.7929988	down	11083	chr20	-

NM_006353	HMGN4	0.00206411	3.792728	down	10473	chr6	+
NM_001466	FZD2	0.01410856	3.7909584	down	2535	chr17	+
NM_001126050	HDGF	0.00117986	3.7899034	down	3068	chr1	-
NM_016328	GTF2IRD1	0.01163832	3.7835991	down	9569	chr7	+
NM_001083	PDE5A	0.01253229	3.7757328	down	8654	chr4	-
NM_031966	CCNB1	0.01052682	3.7731466	down	891	chr5	+
NM_001139517	PRKRA	0.00318521	3.7631805	down	8575	chr2	-
NM_025081	KIAA1305	0.02684831	3.7620625	down	57523	chr14	+
NM_003902	FUBP1	6.04E-05	3.745872	down	8880	chr1	-
NM_198991	KCTD1	0.03945226	3.7455006	down	284252	chr18	-
NM_018443	ZNF302	0.00101539	3.743523	down	55900	chr19	+
NM_003485	GPR68	0.00474317	3.7427433	down	8111	chr14	-
NM_172037	RDH10	0.00228935	3.740682	down	157506	chr8	+
NM_005168	RND3	7.88E-04	3.7401996	down	390	chr2	-
NM_004520	KIF2A	0.00436012	3.7373931	down	3796	chr5	+
NM_153768	CABYR	0.02405559	3.7330809	down	26256	chr18	+
NM_001123383	BCOR	0.01179359	3.7298484	down	54880	chrX	-
NM_015634	KIAA1279	0.00686028	3.7289572	down	26128	chr10	+
NM_023931	ZNF747	5.54E-04	3.7229095	down	65988	chr16	-
NM_012288	TRAM2	6.25E-04	3.7195308	down	9697	chr6	-
NM_015221	DNMBP	0.00249676	3.7150142	down	23268	chr10	-
NM_001042572	CHD2	0.00484382	3.7061646	down	1106	chr15	+
NM_017640	LRRC16	0.00842229	3.705426	down	55604	chr6	+
NM_014425	INVS	0.01103413	3.705355	down	27130	chr9	+
NM_002158	FOXN2	0.00139877	3.698706	down	3344	chr2	+
NM_005534	IFNGR2	0.00247845	3.6973236	down	3460	chr21	+
NM_018353	C14orf106	0.00160238	3.6823754	down	55320	chr14	-
NM_001621	AHR	3.58E-04	3.6722023	down	196	chr7	+
NM_183419	RNF19	0.00989021	3.6672368	down	25897	chr8	-
NM_003320	TUB	0.00144977	3.65419	down	7275	chr11	+
NM_138473	SP1	0.03119052	3.652618	down	6667	chr12	+
NM_001002243	AFTPH	0.04737176	3.6514819	down	54812	chr2	+
NM_130809	PRRC1	0.00473146	3.6514723	down	133619	chr5	+
NM_005734	HIPK3	3.76E-04	3.650046	down	10114	chr11	+
NM_001030007	AP1G1	0.00620527	3.6470325	down	164	chr16	-
NM_004600	TROVE2	0.0230304	3.6462383	down	6738	chr1	+
NM_021214	FAM108C1	9.93E-05	3.6372108	down	58489	chr15	+
NM_012449	STEAP1	0.03281049	3.6327102	down	26872	chr7	+
NM_014689	DOCK10	0.00106268	3.6312733	down	55619	chr2	-
NM_002467	MYC	3.46E-04	3.6285403	down	4609	chr8	+
NM_001081461	JMJD6	0.03020344	3.6236482	down	23210	chr17	-
NM_032102	SFRS2B	4.35E-04	3.620454	down	10929	chr11	+
NM_138555	KIF23	0.00118879	3.6144974	down	9493	chr15	+
NM_024792	FAM57A	0.00135928	3.6096933	down	79850	chr17	+
NM_017920	URG4	0.0057764	3.6026943	down	55665	chr7	-
NM_001042546	ATPAF1	0.02291305	3.6010504	down	64756	chr1	-
NM_032256	TMEM117	1.37E-05	3.6002364	down	84216	chr12	+
NM_173794	FUNDC1	0.04378701	3.5973673	down	139341	chrX	-
NM_017785	CCDC99	9.42E-05	3.5962367	down	54908	chr5	+

NM_014791	MELK	0.00245937	3.5948048	down	9833	chr9	+
NM_020801	ARRDC3	0.00491678	3.5947025	down	57561	chr5	-
NM_001039613	IAH1	8.63E-04	3.594471	down	285148	chr2	+
NM_020731	AHRR	0.00603059	3.5891583	down	57491	chr5	+
NM_004064	CDKN1B	3.33E-05	3.574358	down	1027	chr12	+
NM_153321	PMP22	0.01318984	3.573053	down	5376	chr17	-
NM_001822	CHN1	7.71E-04	3.5670445	down	1123	chr2	-
NM_014035	SNX24	1.21E-04	3.563812	down	28966	chr5	+
NM_133640	SURF5	0.00335349	3.5621233	down	6837	chr9	-
NM_001809	CENPA	7.36E-04	3.5579684	down	1058	chr2	+
NM_198474	OLFML1	0.00123077	3.5536554	down	283298	chr11	+
NM_182588	RGPD4	0.00331072	3.5489824	down	285190	chr2	+
NM_001675	ATF4	0.00301418	3.5448735	down	468	chr22	+
NM_139118	YY1AP1	0.00884741	3.5440922	down	55249	chr1	-
NM_152379	C1orf131	0.00696331	3.537688	down	128061	chr1	-
NM_013441	DSCR1L2	0.00844398	3.536844	down	11123	chr1	+
NM_006537	USP3	0.00882559	3.5350325	down	9960	chr15	+
NM_080867	SOCS4	0.008426	3.5322714	down	122809	chr14	+
NM_144636	CHCHD4	0.00958555	3.5303724	down	131474	chr3	-
NM_003472	DEK	7.29E-06	3.522973	down	7913	chr6	-
NM_052913	KIAA1913	0.04902292	3.515187	down	114801	chr6	+
NM_005270	GLI2	0.02041809	3.5139048	down	2736	chr2	+
NM_001100163	MRVI1	0.01094772	3.5095868	down	10335	chr11	-
NM_018136	ASPM	0.00154471	3.5056417	down	259266	chr1	-
NM_033071	SYNE1	0.02496397	3.5032127	down	23345	chr6	-
NM_001142293	ARFGAP3	0.0280264	3.502032	down	26286	chr22	-
NM_003762	VAMP4	0.00299506	3.4947023	down	8674	chr1	-
NM_153711	FAM26E	0.00131862	3.4945037	down	254228	chr6	+
NM_006190	ORC2L	0.01570034	3.4902546	down	4999	chr2	-
NM_032237	FLJ23356	0.00773372	3.4863772	down	84197	chr8	+
NM_001100594	SNRK	0.00566207	3.4862022	down	54861	chr3	+
NM_015055	SWAP70	8.72E-05	3.4841368	down	23075	chr11	+
NM_001135920	KIAA1432	0.0224952	3.4836578	down	57589	chr9	+
NM_003873	NRP1	0.01820153	3.48038	down	8829	chr10	-
NM_173824	C3orf38	0.01030985	3.4802628	down	285237	chr3	+
NM_032804	C10orf22	0.0031648	3.4756866	down	84890	chr10	+
NM_001080533	MGC5139	0.03931555	3.473951	down	84747	chr12	+
NM_001040022	SIRPA	3.86E-04	3.472695	down	140885	chr20	+
NM_001127183	CFLAR	5.34E-04	3.471348	down	8837	chr2	+
NM_183048	ZMYND8	0.0333094	3.4705334	down	23613	chr20	-
NM_020886	USP28	0.01650212	3.4669874	down	57646	chr11	-
NM_020232	TNFSF5IP1	0.01543654	3.465624	down	56984	chr18	+
NM_001142761	C15orf23	0.00278747	3.462575	down	90417	chr15	+
NM_004654	USP9Y	0.00936595	3.4590085	down	8287	chrY	+
NM_001008895	CUL4A	0.00983392	3.4550893	down	8451	chr13	+
NM_022346	NCAPG	0.00213478	3.4485896	down	64151	chr4	+
NM_178586	PPP2R5C	0.00380169	3.4395013	down	5527	chr14	+
NM_001031700	C4orf18	0.00351466	3.439376	down	51313	chr4	-
NM_016303	WBP5	1.17E-04	3.4337142	down	51186	chrX	+

NM_022071	SH2D4A	0.00401147	3.4329083	down	63898	chr8	+
NM_017896	C20orf11	2.62E-05	3.4324658	down	54994	chr20	+
NM_001077702	MIER1	0.00821585	3.4305978	down	57708	chr1	+
NM_001042604	XRN1	0.01215179	3.4237075	down	54464	chr3	-
NM_153686	LCORL	0.01952454	3.4159827	down	254251	chr4	-
NM_205834	LSR	0.00230189	3.4133284	down	51599	chr19	+
NM_000961	PTGIS	0.02439042	3.413212	down	5740	chr20	-
NM_022566	MESDC1	0.01649011	3.4095938	down	59274	chr15	+
NM_001039887	C19orf55	0.02432732	3.4057229	down	148137	chr19	+
NM_001130079	MOV10	0.04133338	3.4028141	down	4343	chr1	+
NM_016121	KCTD3	0.00418464	3.392671	down	51133	chr1	+
NM_005483	CHAF1A	0.00723745	3.3899844	down	10036	chr19	+
NM_020235	BBX	0.00567651	3.383046	down	56987	chr3	+
NM_001071	TYMS	0.01085412	3.3830369	down	7298	chr18	+
NM_018047	RBM22	0.01632459	3.3810036	down	55696	chr5	-
NM_003575	ZNF282	0.02581786	3.3785393	down	8427	chr7	+
NM_020245	TULP4	0.01644313	3.3780134	down	56995	chr6	+
NM_002358	MAD2L1	9.14E-04	3.3757353	down	4085	chr4	-
NM_033319	CENPL	0.01033751	3.374022	down	91687	chr1	-
NM_002518	NPAS2	7.42E-04	3.3737075	down	4862	chr2	+
NM_001049	SSTR1	0.00217387	3.3717167	down	6751	chr14	+
NM_022130	GOLPH3	2.93E-04	3.3696277	down	64083	chr5	-
NM_014918	CHSY1	0.035538	3.3677273	down	22856	chr15	-
NM_015695	BRPF3	5.81E-04	3.3668356	down	27154	chr6	+
NM_000044	AR	0.00148236	3.3583956	down	367	chrX	+
NM_019043	APBB1IP	0.00996474	3.358075	down	54518	chr10	+
NM_152261	C12orf23	0.01387094	3.356081	down	90488	chr12	+
NM_130898	CREB3L4	0.00156116	3.3555176	down	148327	chr1	+
NM_015443	KIAA1267	0.0011486	3.3520136	down	284058	chr17	-
NM_003705	SLC25A12	0.00693526	3.351637	down	8604	chr2	-
NM_001006640	TCEAL1	9.47E-04	3.3466125	down	9338	chrX	+
NM_182660	UTY	0.01308562	3.3398898	down	7404	chrY	-
NM_001130842	ZNF286A	0.01543883	3.3389075	down	57335	chr17	+
NM_144572	TBC1D2B	0.00280436	3.3380048	down	23102	chr15	-
NM_020169	LXN	0.00106221	3.3348553	down	56925	chr3	-
NM_005080	XBP1	0.00397372	3.3347163	down	7494	chr22	-
NM_005496	SMC4	0.0014405	3.3326824	down	10051	chr3	+
NM_145739	OSBPL6	0.00143691	3.3307257	down	114880	chr2	+
NM_001040409		0.02407987	3.330621	down			
NM_014473	DIMT1L	0.0473281	3.3258786	down	27292	chr5	-
NM_006169	NNMT	0.00279903	3.3234668	down	4837	chr11	+
NM_001134376	CCNJ	0.03682208	3.3227913	down	54619	chr10	+
NM_003592	CUL1	1.06E-04	3.3211958	down	8454	chr7	+
NM_016265	ZNF12	0.01941443	3.3155277	down	7559	chr7	-
NM_001034	RRM2	0.00476214	3.3132284	down	6241	chr2	+
NM_024297	PHF23	0.02373238	3.3126566	down	79142	chr17	-
NM_001290	LDB2	0.012764	3.3053596	down	9079	chr4	-
NM_018571	ALS2CR2	0.02068527	3.298071	down	55437	chr2	+
NM_015339	ADNP	0.00272514	3.2978551	down	23394	chr20	-

NM_001002800	SMC4	0.00339611	3.294223	down	10051	chr3	+
NM_080550	AP1GBP1	0.00270698	3.2931552	down	11276	chr17	-
NM_000671	ADH5	0.0175812	3.2908583	down	128	chr4	-
NM_014703	VPRBP	0.04483065	3.2904873	down	9730	chr3	-
NM_003390	WEE1	0.02764049	3.288888	down	7465	chr11	+
NM_006386	DDX17	0.02795106	3.2859716	down	10521	chr22	-
NM_001130067	TRIM2	0.00713532	3.2853515	down	23321	chr4	+
NM_001080392	KIAA1147	2.18E-05	3.284608	down	57189	chr7	-
NM_005626	SFRS4	3.33E-04	3.283632	down	6429	chr1	-
NM_001806	CEBPG	0.03501173	3.2807822	down	1054	chr19	+
NM_199334	THRA	0.00272431	3.2794898	down	7067	chr17	+
NM_006884	SHOX2	1.50E-05	3.2772806	down	6474	chr3	-
NM_004494	HDGF	0.00362531	3.2745118	down	3068	chr1	-
NM_025134	CHD9	5.35E-04	3.270085	down	80205	chr16	+
NM_001042728	RARG	1.23E-04	3.269133	down	5916	chr12	-
NM_173468	MOBKL1A	0.02077737	3.2682915	down	92597	chr4	+
NM_000208	INSR	0.00239863	3.2602298	down	3643	chr19	-
NM_003453	ZMYM2	0.00183926	3.2598453	down	7750	chr13	+
NM_178007	STARD13	0.02772206	3.2584362	down	90627	chr13	-
NM_016586	MBIP	2.70E-04	3.2574046	down	51562	chr14	-
NM_001013739	LOC442578	0.00265887	3.2570753	down	442578	chr7	-
NM_014321	ORC6L	0.00136297	3.256805	down	23594	chr16	+
NM_022151	MOAP1	0.00496143	3.256507	down	64112	chr14	-
NM_001123384	BCOR	0.04720151	3.2554953	down	54880	chrX	-
NM_001100423	LOC26010	0.0043552	3.252501	down	26010	chr2	+
NM_001001938	C9orf47	0.01307757	3.2497792	down	286223	chr9	+
NM_205850	SLC24A5	0.00633769	3.2473173	down	283652	chr15	+
NM_133458	ZFP90	0.00578181	3.2434802	down	146198	chr16	+
NM_001142571	RAD51L3	8.26E-04	3.2354126	down	5892	chr17	-
NM_001029882	AHDC1	0.004595	3.2351153	down	27245	chr1	-
NM_001042683	SHPRH	0.01341669	3.2339468	down	257218	chr6	-
NM_014722	FAM65B	0.01186319	3.2338336	down	9750	chr6	-
NM_001129891	LOC10013189	0.00194718	3.2335439	down	100131897	chr5	-
NM_058241	CCNT2	0.00763029	3.2331078	down	905	chr2	+
NM_024315	C7orf23	0.0025553	3.2241495	down	79161	chr7	-
NM_006988	ADAMTS1	0.00589165	3.2235515	down	9510	chr21	-
NM_002485	NBN	0.01186099	3.2207232	down	4683	chr8	-
NM_004348	RUNX2	0.03969744	3.2162626	down	860	chr6	+
NM_012238	SIRT1	0.00579197	3.2114604	down	23411	chr10	+
NM_181656	C17orf58	0.01310024	3.2086742	down	284018	chr17	-
NM_001130009	GEN1	1.43E-04	3.2003534	down	348654	chr2	+
NM_001136195	TNPO2	0.02678374	3.1976805	down	30000	chr19	-
NM_207292	MBNL1	0.00152124	3.1969259	down	4154	chr3	+
NM_001014445	NLE1	0.01633326	3.196553	down	54475	chr17	-
NM_005842	SPRY2	0.00445087	3.1915574	down	10253	chr13	-
NM_020724	RNF150	0.01720033	3.1903942	down	57484	chr4	-
NM_013372	GREM1	0.00641999	3.18981	down	26585	chr15	+
NM_001100621	C17orf80	0.00463677	3.1888475	down	55028	chr17	+
NM_020714	ZNF490	0.00336915	3.188089	down	57474	chr19	-

NM_001439	EXTL2	0.00815993	3.184641	down	2135	chr1	-
NM_057159	EDG2	1.15E-04	3.1843598	down	1902	chr9	-
NM_001135811	FAM60A	0.03721422	3.1816003	down	58516	chr12	-
NM_015483	KBTBD2	0.00403148	3.1796007	down	25948	chr7	-
NM_001048197	SNHG3-RCC1	0.00112601	3.1772654	down	751867	chr1	+
NM_022739	SMURF2	9.70E-04	3.1767752	down	64750	chr17	-
NM_001124	ADM	6.00E-05	3.1766818	down	133	chr11	+
NM_145021	8-Mar	0.02259643	3.1757274	down	220972	chr10	-
NM_020762	SRGAP1	0.00267066	3.1737263	down	57522	chr12	+
NM_030672	ARHGAP28	0.00407964	3.169946	down	79822	chr18	+
NM_004606	TAF1	0.00192824	3.1680315	down	6872	chrX	+
NM_173557	RNF152	2.17E-04	3.1672053	down	220441	chr18	-
NM_130398	EXO1	0.01197797	3.1662319	down	9156	chr1	+
NM_022769	CRTC3	0.00110297	3.1642516	down	64784	chr15	+
NM_018086	FIGN	0.00109535	3.1622348	down	55137	chr2	-
NM_014350	TNFAIP8	5.50E-04	3.1622076	down	25816	chr5	+
NM_181442	ADNP	0.04143131	3.161007	down	23394	chr20	-
NM_001144013	RGPD3	0.0086857	3.1607907	down	653489	chr2	-
NM_004865	TBPL1	0.00187432	3.160285	down	9519	chr6	+
NM_007172	NUP50	0.00857061	3.1593294	down	10762	chr22	+
NM_020194	C2orf33	0.00517872	3.1569517	down	56947	chr2	+
NM_001145664	LOC731220	0.00641097	3.1539052	down	731220	chr2	-
NM_020905	RDH14	0.00242653	3.1525607	down	57665	chr2	-
NM_013942	PAX3	0.00828499	3.1524847	down	5077	chr2	-
NM_003777	DNAH11	0.00179656	3.1515574	down	8701	chr7	+
NM_001274	CHEK1	0.01977916	3.150794	down	1111	chr11	+
NM_001131027	PDLIM4	0.00432804	3.1500568	down	8572	chr5	+
NM_003257	TJP1	0.00568483	3.148378	down	7082	chr15	-
NM_003152	STAT5A	0.02771885	3.1455674	down	6776	chr17	+
NM_007006	NUDT21	0.02385491	3.1440115	down	11051	chr16	-
NM_023927	GRAMD3	0.02548988	3.1436334	down	65983	chr5	+
NM_006881	MDM2	0.0417188	3.140796	down	4193	chr12	+
NM_182757	IBRDC2	0.02471125	3.1396043	down	255488	chr6	+
NM_021649	TICAM2	0.01301768	3.1369386	down	353376	chr5	-
NM_006885	ATBF1	0.00705005	3.1366246	down	463	chr16	-
NM_198887	NUP43	4.67E-04	3.1361217	down	348995	chr6	-
NM_052947	ALPK2	5.89E-04	3.1289437	down	115701	chr18	-
NM_032195	SON	0.01204558	3.1277928	down	6651	chr21	+
NM_002874	RAD23B	0.0184028	3.1254861	down	5887	chr9	+
NM_001121	ADD3	0.01355123	3.1240351	down	120	chr10	+
NM_001006622	WDR33	0.00134831	3.1228108	down	55339	chr2	-
NM_006090	CEPT1	0.04161441	3.120183	down	10390	chr1	+
NM_003129	SQLE	0.005167	3.1119673	down	6713	chr8	+
NM_004491	GRLF1	0.00139561	3.109653	down	2909	chr19	+
NM_005228	EGFR	6.99E-04	3.1010242	down	1956	chr7	+
NM_014044	UNC50	0.00112496	3.0993211	down	25972	chr2	+
NM_021089	ZNF8	0.00221088	3.0991595	down	7554	chr19	+
NM_021238	FAM60A	0.00148871	3.0948498	down	58516	chr12	-
NM_001025201	CHN1	0.00541589	3.09404	down	1123	chr2	-

NM_024090	ELOVL6	0.00622148	3.0906303	down	79071	chr4	-
NM_207037	TCF12	7.28E-05	3.0889657	down	6938	chr15	+
NM_017969	IWS1	0.00617092	3.0870938	down	55677	chr2	-
NM_181312	TAZ	0.0023905	3.0864816	down	6901	chrX	+
NM_145686	MAP4K4	0.00369049	3.0859838	down	9448	chr2	+
NM_144573	NEXN	0.03455432	3.0847774	down	91624	chr1	+
NM_002757	MAP2K5	0.00190964	3.0830762	down	5607	chr15	+
NM_001098504	DDX17	1.09E-04	3.0820942	down	10521	chr22	-
NM_001042581	SNUPN	0.02570267	3.0818548	down	10073	chr15	-
NM_198041	NUDT6	8.64E-04	3.0812345	down	11162	chr4	-
NM_020310	MNT	0.01216238	3.0805562	down	4335	chr17	-
NM_005484	PARP2	0.00672318	3.0792665	down	10038	chr14	+
NM_001083893	STRN3	1.43E-05	3.0762117	down	29966	chr14	-
NM_001104647	SLC25A36	0.00608463	3.0760896	down	55186	chr3	+
NM_018989	RBM27	0.0116108	3.072654	down	54439	chr5	+
NM_032852	ATG4C	0.00423195	3.071509	down	84938	chr1	+
NM_012404	ANP32D	0.0076441	3.0708642	down	23519	chr12	+
NM_004130	GYG1	0.00145458	3.0680885	down	2992	chr3	+
NM_032181	TMEM166	0.00635992	3.0676808	down	84141	chr2	-
NM_032367	ZBED3	9.60E-04	3.0606644	down	84327	chr5	-
NM_012250	RRAS2	0.0200156	3.0576897	down	22800	chr11	-
NM_001679	ATP1B3	0.0149062	3.0572422	down	483	chr3	+
NM_198057	TSC22D3	0.01673647	3.0564842	down	1831	chrX	-
NM_024607	PPP1R3B	0.00313618	3.0560348	down	79660	chr8	-
NM_005667	RNF103	3.62E-04	3.0551834	down	7844	chr2	-
NM_182691	SRPK2	0.00142178	3.053446	down	6733	chr7	-
NM_015021	ZNF292	0.00682615	3.0509865	down	23036	chr6	+
NM_015911	ZNF691	0.01182012	3.0499551	down	51058	chr1	+
NM_006449	CDC42EP3	0.00119771	3.0480897	down	10602	chr2	-
NM_002148	HOXD10	0.00190838	3.0443666	down	3236	chr2	+
NM_145014	HYLS1	0.00196644	3.038529	down	219844	chr11	+
NM_013436	NCKAP1	0.00162066	3.0376663	down	10787	chr2	-
NM_033512	TSPYL5	0.02730212	3.0346198	down	85453	chr8	-
NM_014847	UBAP2L	6.63E-06	3.033182	down	9898	chr1	+
NM_003939	BTRC	0.04234272	3.0316012	down	8945	chr10	+
NM_170735	BDNF	0.00802525	3.0231411	down	627	chr11	-
NM_024597	MAP7D3	0.02349256	3.0204186	down	79649	chrX	-
NM_152581	MOSPD2	0.02101004	3.0198765	down	158747	chrX	+
NM_006670	TPBG	4.18E-04	3.0183048	down	7162	chr6	+
NM_012180	FBXO8	0.02826995	3.01707	down	26269	chr4	-
NM_003247	THBS2	0.00650993	3.0157542	down	7058	chr6	-
NM_001006613	WBP5	0.00465715	3.0150526	down	51186	chrX	+
NM_004273	CHST3	0.00120727	3.0144367	down	9469	chr10	+
NM_006015	ARID1A	2.58E-04	3.0137851	down	8289	chr1	+
NM_020378	NAT14	0.00180862	3.0068939	down	57106	chr19	+
NM_007207	DUSP10	0.00400082	3.0068066	down	11221	chr1	-
NM_001008224	UACA	0.01011727	3.0060663	down	55075	chr15	-
NM_019054	FAM35A	0.00184343	3.0056221	down	54537	chr10	+
NM_005226	EDG3	0.00413312	3.0048752	down	1903	chr9	+

NM_014326	DAPK2	0.00444465	3.001516	down	23604	chr15	-
NM_001042762	FIGNL1	0.00277092	2.9941335	down	63979	chr7	-
NM_173084	TRIM59	0.00276413	2.9941196	down	286827	chr3	-
NM_001127370	CDCA7L	9.83E-04	2.9926484	down	55536	chr7	-
NM_013390	TMEM2	0.04948927	2.9869208	down	23670	chr9	-
NM_022838	ARMCX5	0.01222218	2.9866476	down	64860	chrX	+
NM_006441	MTHFS	0.03208034	2.985323	down	10588	chr15	-
NM_007019	UBE2C	0.00532746	2.9829097	down	11065	chr20	+
NM_145798	OSBPL7	0.0080711	2.9825408	down	114881	chr17	-
NM_003410	ZFX	0.02298259	2.9824452	down	7543	chrX	+
NM_001949	E2F3	0.00736402	2.981444	down	1871	chr6	+
NM_032642	WNT5B	0.0047609	2.980361	down	81029	chr12	+
NM_181523	PIK3R1	0.00505611	2.9769824	down	5295	chr5	+
NM_001003682	TTMB	0.00736776	2.9761677	down	399474	chr1	-
NM_017693	BIVM	0.01664076	2.9750051	down	54841	chr13	+
NM_032408	BAZ1B	5.34E-04	2.9749029	down	9031	chr7	-
NM_005180	BMI1	0.00349258	2.973451	down	648	chr10	+
NM_001128636	ELFN1	3.61E-04	2.9651365	down	392617	chr7	+
NM_001017415	USP1	0.03101261	2.9623976	down	7398	chr1	+
NM_032427	MAML2	0.00835607	2.9605625	down	84441	chr11	-
NM_004326	BCL9	0.01055646	2.9596124	down	607	chr1	+
NM_021872	CDC25B	0.03194456	2.9594157	down	994	chr20	+
NM_001777	CD47	0.01237044	2.9572031	down	961	chr3	-
NM_017542	POGK	0.03141721	2.952442	down	57645	chr1	+
NM_005707	PDCD7	0.01840263	2.9511716	down	10081	chr15	-
NM_052905	FMNL2	1.17E-04	2.9475648	down	114793	chr2	+
NM_016374	ARID4B	0.04922053	2.9464574	down	51742	chr1	-
NM_024496	C14orf4	0.00679568	2.9459658	down	64207	chr14	-
NM_021198	CTDSP1	0.02604948	2.94107	down	58190	chr2	+
NM_033550	TP53RK	3.32E-04	2.9385128	down	112858	chr20	-
NM_001033031	FAIM	0.00256435	2.9376135	down	55179	chr3	+
NM_182715	SYPL1	0.00803048	2.9359643	down	6856	chr7	-
NM_005497	GJA7	3.52E-06	2.9350882	down	10052	chr17	-
NM_022166	XYLT1	4.15E-05	2.9347167	down	64131	chr16	-
NM_005342	HMGB3	0.00315863	2.9343007	down	3149	chrX	+
NM_020947	KIAA1609	0.006671	2.933165	down	57707	chr16	-
NM_005494	DNAJB6	0.00100952	2.9307609	down	10049	chr7	+
NM_012287	CENTB2	0.01803801	2.930418	down	23527	chr3	-
NM_002403	MFAP2	0.0070587	2.9299366	down	4237	chr1	-
NM_078626	CDKN2C	9.59E-04	2.9292006	down	1031	chr1	+
NM_004926	ZFP36L1	0.01658882	2.92901	down	677	chr14	-
NM_025139	ARMC9	0.0093276	2.9289637	down	80210	chr2	+
NM_000381	MID1	0.03884151	2.9274867	down	4281	chrX	-
NM_001143853	WIBG	0.0020831	2.926701	down	84305	chr12	-
NM_016101	NIP7	0.01715371	2.9265242	down	51388	chr16	+
NM_005012	ROR1	0.00400095	2.926523	down	4919	chr1	+
NM_014913	ZNF508	0.00792205	2.9258752	down	22850	chr18	+
NM_133433	NIPBL	0.00385056	2.9228604	down	25836	chr5	+
NM_001136022	NFATC4	5.05E-04	2.92281	down	4776	chr14	+

NM_201282	EGFR	0.00420694	2.918525	down	1956	chr7	+
NM_003153	STAT6	0.01508174	2.9145958	down	6778	chr12	-
NM_172244	SGCD	0.0022906	2.914461	down	6444	chr5	+
NM_153769	CABYR	0.00900244	2.9140835	down	26256	chr18	+
NM_138347	ZNF551	0.01233299	2.9106114	down	90233	chr19	+
NM_015214	DDHD2	2.34E-05	2.9084275	down	23259	chr8	+
NM_001130709	RUFY3	6.66E-04	2.9082613	down	22902	chr4	+
NM_198066	GNPNAT1	0.01258689	2.9066823	down	64841	chr14	-
NM_138499	PWWP2	0.02998346	2.9066505	down	170394	chr10	+
NM_170753	PGBD3	0.00903432	2.9047577	down	267004	chr10	-
NM_007159	SLMAP	0.04433086	2.9045656	down	7871	chr3	+
NM_004101	F2RL2	0.04656069	2.9023643	down	2151	chr5	-
NM_016831	PER3	0.01058187	2.9015307	down	8863	chr1	+
NM_001790	CDC25C	0.00215178	2.8930426	down	995	chr5	-
NM_023930	KCTD14	0.00199658	2.8879256	down	65987	chr11	-
NM_001145513	SCRN1	0.00163571	2.8868692	down	9805	chr7	-
NM_001080469	FBXO46	0.00283475	2.884314	down	23403	chr19	-
NM_144726	RNF145	0.00565047	2.883909	down	153830	chr5	-
NM_022802	CTBP2	0.01046816	2.8826091	down	1488	chr10	-
NM_170695	TGIF1	9.64E-04	2.881685	down	7050	chr18	+
NM_003484	HMGA2	0.04857101	2.8810394	down	8091	chr12	+
NM_001077401	ACVRL1	0.01588838	2.880749	down	94	chr12	+
NM_000627	LTBP1	0.00347159	2.880355	down	4052	chr2	+
NM_181340	WDR21A	0.0019083	2.8797352	down	26094	chr14	+
NM_007042	RPP14	0.02561485	2.8789737	down	11102	chr3	+
NM_012207	HNRPH3	0.00107954	2.8764951	down	3189	chr10	+
NM_005359	SMAD4	4.07E-06	2.8755388	down	4089	chr18	+
NM_003318	TTK	0.00374502	2.8720396	down	7272	chr6	+
NM_001102399	HNRNPR	0.01932396	2.870544	down	10236	chr1	-
NM_001042552	TATDN3	0.00192662	2.867941	down	128387	chr1	+
NM_001004051	GPRASP2	0.00241866	2.8678062	down	114928	chrX	+
NM_003607	CDC42BPA	0.0062581	2.8671513	down	8476	chr1	-
NM_001142522	FBXO5	7.85E-05	2.8671002	down	26271	chr6	-
NM_005126	NR1D2	0.00189226	2.862699	down	9975	chr3	+
NM_153693	HOXC6	0.00860825	2.86005	down	3223	chr12	+
NM_001145102	SMAD3	1.46E-04	2.8596501	down	4088	chr15	+
NM_001122	ADFP	1.48E-05	2.8586428	down	123	chr9	-
NM_018230	NUP133	0.02986016	2.858536	down	55746	chr1	-
NM_012102	RERE	0.01924548	2.8580136	down	473	chr1	-
NM_005983	SKP2	0.00279883	2.8573089	down	6502	chr5	+
NM_004454	ETV5	0.00348278	2.8558512	down	2119	chr3	-
NM_001200	BMP2	5.11E-04	2.8553631	down	650	chr20	+
NM_145061	C13orf3	0.00789817	2.8551536	down	221150	chr13	-
NM_139015	UNQ1887	0.00169319	2.854463	down	121665	chr12	-
NM_138410	CMTM7	0.04020856	2.8532019	down	112616	chr3	+
NM_001080417	ZNF629	0.00119461	2.853198	down	23361	chr16	-
NM_004376	COX15	0.0327361	2.8530972	down	1355	chr10	-
NM_006565	CTCF	0.0441709	2.8530293	down	10664	chr16	+
NM_012154	EIF2C2	0.00460364	2.8527286	down	27161	chr8	-

NM_001010883	FAM102B	5.81E-04	2.852212 down	284611	chr1	+
NM_001006614	WBP5	0.00317818	2.848116 down	51186	chrX	+
NM_152736	ZNF187	0.02474813	2.8480444 down	7741	chr6	+
NM_032211	LOXL4	0.00199172	2.8478994 down	84171	chr10	-
NM_003998	NFKB1	0.00462982	2.8447647 down	4790	chr4	+
NM_014949	KIAA0907	0.00451929	2.8423314 down	22889	chr1	-
NM_006029	PNMA1	4.65E-05	2.8422818 down	9240	chr14	-
NM_001135597	CCDC88A	0.00528723	2.8402476 down	55704	chr2	-
NM_000089	COL1A2	0.00353724	2.834454 down	1278	chr7	+
NM_001114122	CHEK1	0.00838774	2.8311377 down	1111	chr11	+
NM_012112	TPX2	0.00118279	2.8285112 down	22974	chr20	+
NM_001001420	SMAD5	0.00642909	2.8284523 down	4090	chr5	+
NM_001037305	THYN1	0.00685456	2.8284216 down	29087	chr11	-
NM_001077664	URG4	9.22E-04	2.8283224 down	55665	chr7	-
NM_152289	ZNF561	0.01706095	2.8236382 down	93134	chr19	-
NM_031465	C12orf32	0.00559052	2.8234189 down	83695	chr12	+
NM_014048	MKL2	2.19E-04	2.8230615 down	57496	chr16	+
NM_001003702	FLJ43692	0.03542871	2.8190103 down	445328	chr7	-
NM_015042	ZNF609	0.01743422	2.8165665 down	23060	chr15	+
NM_203351	MAP3K3	0.00226181	2.812213 down	4215	chr17	+
NM_003749	IRS2	0.0463906	2.8083825 down	8660	chr13	-
NM_153240	NPHP3	1.48E-04	2.8075461 down	27031	chr3	-
NM_004242	HMGN3	0.011844	2.8074934 down	9324	chr6	-
NM_000028	AGL	0.01184442	2.8060665 down	178	chr1	+
NM_201613	IKIP	0.04885346	2.8059218 down	121457	chr12	-
NM_015549	PLEKHG3	0.00605484	2.8054535 down	26030	chr14	+
NM_198595	AFAP1	0.01092085	2.804025 down	60312	chr4	-
NM_006876	B3GNT1	7.43E-04	2.8002768 down	11041	chr11	-
NM_001135248	MFAP2	0.01138885	2.796068 down	4237	chr1	-
NM_012194	C11orf41	0.00687428	2.7908459 down	25758	chr11	+
NM_001142565	FLJ12529	0.04237116	2.7893214 down	79869	chr11	-
NM_144596	TTC8	0.00530762	2.7851012 down	123016	chr14	+
NM_015139	SLC35D1	0.00949309	2.7769868 down	23169	chr1	-
NM_006079	CITED2	0.00169709	2.7758856 down	10370	chr6	-
NM_005308	GRK5	0.01813026	2.7754855 down	2869	chr10	+
NM_017657	AFTPH	0.01529405	2.7722712 down	54812	chr2	+
NM_016272	TOB2	0.00352513	2.7708082 down	10766	chr22	-
NM_019042	PUS7	0.00163786	2.7689707 down	54517	chr7	-
NM_021873	CDC25B	0.00467885	2.7681565 down	994	chr20	+
NM_001080470	ZNF697	0.00335058	2.7680795 down	90874	chr1	-
NM_152913	TMEM130	0.0058339	2.7678928 down	222865	chr7	-
NM_005902	SMAD3	0.00630722	2.7667625 down	4088	chr15	+
NM_024675	PALB2	0.00603521	2.765413 down	79728	chr16	-
NM_015906	TRIM33	0.01421121	2.7630422 down	51592	chr1	-
NM_212479	ZMYND11	0.00832543	2.7628503 down	10771	chr10	+
NM_018647	TNFRSF19	0.00238131	2.7600493 down	55504	chr13	+
NM_003070	SMARCA2	0.0159893	2.7598777 down	6595	chr9	+
NM_006792	MORF4	0.0269114	2.7597098 down	10934	chr4	-
NM_020239	CDC42SE1	0.0343963	2.7583475 down	56882	chr1	-

NM_014802	KIAA0528	0.03444291	2.7575846	down	9847	chr12	-
NM_016498	MTP18	0.01082451	2.7556367	down	51537	chr22	+
NM_001018068	SERBP1	0.00765514	2.754919	down	26135	chr1	-
NM_003482	MLL2	0.00827424	2.7545085	down	8085	chr12	-
NM_007235	XPOT	0.00557442	2.7527046	down	11260	chr12	+
NM_007211	RASSF8	0.01456551	2.7513366	down	11228	chr12	+
NM_003713	PPAP2B	0.02044498	2.7480066	down	8613	chr1	-
NM_005885	MARCH6	0.00173358	2.7463195	down	10299	chr5	+
NM_005047	PSMD5	0.02268883	2.7450218	down	5711	chr9	-
NM_005207	CRKL	0.00522233	2.7438128	down	1399	chr22	+
NM_003507	FZD7	3.77E-04	2.7407928	down	8324	chr2	+
NM_025198	MTERFD3	8.34E-04	2.7367609	down	80298	chr12	-
NM_018383	WDR33	8.80E-04	2.7365093	down	55339	chr2	-
NM_006510	TRIM27	0.01099357	2.7355833	down	5987	chr6_cox_hap1	-
NM_015641	TES	0.00421704	2.7333636	down	26136	chr7	+
NM_004620	TRAF6	0.0025919	2.7329247	down	7189	chr11	-
NM_203459	CAMSAP1L1	0.02851856	2.726482	down	23271	chr1	+
NM_032144	RAB6C	0.01331964	2.7262602	down	84084	chr2	+
NM_003045	SLC7A1	8.70E-04	2.7257934	down	6541	chr13	-
NM_013229	APAF1	0.02981225	2.725108	down	317	chr12	+
NM_152271	LONRF1	0.00936948	2.7244694	down	91694	chr8	-
NM_007282	RNF13	0.02957254	2.721952	down	11342	chr3	+
NM_001136494	C1orf198	0.01252949	2.7218719	down	84886	chr1	-
NM_139157	ST5	0.00941483	2.7214258	down	6764	chr11	-
NM_002075	GNB3	0.00282802	2.720789	down	2784	chr12	+
NM_003794	SNX4	3.19E-04	2.7200077	down	8723	chr3	-
NM_197968	ZMYM2	0.00219699	2.7193944	down	7750	chr13	+
NM_018027	FRMD4A	2.90E-04	2.7192364	down	55691	chr10	-
NM_018062	FANCL	0.0449477	2.7188401	down	55120	chr2	-
NM_022474	MPP5	0.00310514	2.7165027	down	64398	chr14	+
NM_002056	GFPT1	0.00498977	2.7129092	down	2673	chr2	-
NM_000304	PMP22	0.00717985	2.7127254	down	5376	chr17	-
NM_139321	ATRN	0.00207397	2.7125676	down	8455	chr20	+
NM_014764	DAZAP2	0.0103335	2.711268	down	9802	chr12	+
NM_003619	PRSS12	0.00148484	2.7096043	down	8492	chr4	-
NM_181491	MED22	1.55E-04	2.7088926	down	6837	chr9	-
NM_205842	NCKAP1	0.00964182	2.7081816	down	10787	chr2	-
NM_032637	SKP2	1.40E-04	2.705774	down	6502	chr5	+
NM_015169	RRS1	0.03529555	2.7057338	down	23212	chr8	+
NM_024325	ZNF343	0.00903595	2.7041907	down	79175	chr20	-
NM_022145	CENPK	0.00529906	2.7011375	down	64105	chr5	-
NM_001211	BUB1B	0.00174847	2.7005973	down	701	chr15	+
NM_001104595	FAM118A	0.00128343	2.700119	down	55007	chr22	+
NM_178835	LOC152485	4.95E-04	2.6965613	down	152485	chr4	-
NM_002336	LRP6	0.01705883	2.694636	down	4040	chr12	-
NM_032947	MST150	0.00652557	2.6928349	down	85027	chr5	+
NM_004739	MTA2	0.00352666	2.692801	down	9219	chr11	-
NM_016578	RSF1	0.00593364	2.6918917	down	51773	chr11	-
NM_016426	GTSE1	0.0016646	2.6900682	down	51512	chr22	+

NM_001488	TADA2L	0.00375029	2.6897874	down	6871	chr17	+
NM_000123	ERCC5	0.00321119	2.6897395	down	2073	chr13	+
NM_018370	DRAM	0.00789748	2.6889448	down	55332	chr12	+
NM_024429	PRKAG2	0.03832503	2.6884089	down	51422	chr7	-
NM_014706	SART3	0.04882195	2.6871233	down	9733	chr12	-
NM_198507	TMEM157	0.02738652	2.6863012	down	345757	chr5	+
NM_004854	CHST10	0.03923923	2.6855493	down	9486	chr2	-
NM_014890	FILIP1L	0.00276292	2.6839104	down	11259	chr3	-
NM_001033112	PAIP2	0.03104478	2.683597	down	51247	chr5	+
NM_000393	COL5A2	1.73E-04	2.6830056	down	1290	chr2	-
NM_001112734	ZNF397OS	0.00281968	2.681386	down	100101467	chr18	-
NM_021982	SEC24A	0.00985933	2.680764	down	10802	chr5	+
NM_012300	FBXW11	0.00763404	2.6775477	down	23291	chr5	-
NM_032287	LDOC1L	1.68E-04	2.6774018	down	84247	chr22	-
NM_020205	OTUD7B	0.00846114	2.677153	down	56957	chr1	-
NM_017530	LOC55565	1.67E-04	2.676768	down	55565	chr16	-
NM_005107	ENDOGL1	0.0198105	2.676425	down	9941	chr3	+
NM_003846	PEX11B	0.00474087	2.676333	down	8799	chr1	+
NM_001142279	RNASEH2B	0.02784127	2.6749806	down	79621	chr13	+
NM_198310	TTC8	0.00145712	2.674895	down	123016	chr14	+
NM_024835	ZNF403	0.01101465	2.6745625	down	79893	chr17	+
NM_031217	KIF18A	0.0044148	2.6720788	down	81930	chr11	-
NM_080668	CDCA5	0.00100367	2.671978	down	113130	chr11	-
NM_000314	PTEN	0.00156145	2.671444	down	5728	chr10	+
NM_004230	EDG5	0.00508356	2.6706493	down	9294	chr19	-
NM_020983	ADCY6	0.03850253	2.670261	down	112	chr12	-
NM_001300	KLF6	9.03E-05	2.6660385	down	1316	chr10	-
NM_006656	NEU3	0.00843873	2.66145	down	10825	chr11	+
NM_130385	MRVI1	0.00523911	2.6610875	down	10335	chr11	-
NM_001080463	DYNC2H1	0.00862792	2.6606548	down	79659	chr11	+
NM_152649	MLKL	0.0025165	2.65668	down	197259	chr16	-
NM_018657	MYNN	0.01266528	2.655926	down	55892	chr3	+
NM_024493	ZKSCAN3	0.01964921	2.654042	down	80317	chr6	+
NM_198794	MAP4K5	0.01865008	2.6538236	down	11183	chr14	-
NM_015397	WDR40A	0.03518948	2.6535473	down	25853	chr9	-
NM_016074	BOLA1	4.84E-04	2.6514695	down	51027	chr1	+
NM_000430	PAFAH1B1	0.00979338	2.6500144	down	5048	chr17	+
NM_004833	AIM2	0.00199774	2.649953	down	9447	chr1	-
NM_012248	SEPHS2	0.00180337	2.6483192	down	22928	chr16	-
NM_003866	INPP4B	0.02780627	2.6473863	down	8821	chr4	-
NM_024600	C16orf30	0.01501899	2.6464834	down	79652	chr16	+
NM_014903	NAV3	0.0491754	2.6401248	down	89795	chr12	+
NM_022757	CCDC14	0.03227716	2.639904	down	64770	chr3	-
NM_001127713	ATL1	0.01574433	2.6378047	down	51062	chr14	+
NM_031433	MFRP	0.02886966	2.6372626	down	83552	chr11	-
NM_000332	ATXN1	0.00118561	2.6355798	down	6310	chr6	-
NM_005148	UNC119	0.02336465	2.633323	down	9094	chr17	-
NM_014191	SCN8A	0.004525	2.6312063	down	6334	chr12	+
NM_018715	RCC2	0.00354795	2.6310306	down	55920	chr1	-

NM_153449	SLC2A14	0.02149189	2.6278775	down	144195 chr12	-
NM_153033	KCTD7	0.04990126	2.6269205	down	154881 chr7	+
NM_017669	ERCC6L	0.03785986	2.6265142	down	54821 chrX	-
NM_007145	ZNF146	0.00425747	2.6232574	down	7705 chr19	+
NM_004779	CNOT8	0.00967342	2.6226306	down	9337 chr5	+
NM_144721	THAP6	0.0455623	2.6204262	down	152815 chr4	+
NM_001955	EDN1	2.40E-04	2.6202357	down	1906 chr6	+
NM_145160	MAP2K5	0.00103972	2.6189587	down	5607 chr15	+
NM_001098525	CKAP2	0.02427479	2.6178794	down	26586 chr13	+
NM_000168	GLI3	0.00460105	2.6167696	down	2737 chr7	-
NM_033411	RWDD2	0.03999583	2.6136258	down	112611 chr6	+
NM_032051	PATZ1	5.13E-04	2.6114404	down	23598 chr22	-
NM_001136027	VRK2	9.31E-04	2.6088004	down	7444 chr2	+
NM_003341	UBE2E1	0.00542389	2.6087213	down	7324 chr3	+
NM_206861	TACC2	0.00217554	2.6086547	down	10579 chr10	+
NM_198128	RNF138	0.02746593	2.6071575	down	51444 chr18	+
NM_001031695	RBM9	0.00706887	2.6067111	down	23543 chr22	-
NM_001098511	KIF2A	0.00146012	2.6057172	down	3796 chr5	+
NM_031486	ZNF484	0.00747485	2.605519	down	83744 chr9	-
NM_001102575	SNX18	1.10E-04	2.6054363	down	112574 chr5	+
NM_020814	MARCH4	0.00428928	2.6045685	down	57574 chr2	-
NM_015874	RBPJ	0.04177477	2.6041243	down	3516 chr4	+
NM_001122633	CPS1	0.00688587	2.603664	down	1373 chr2	+
NM_005022	PFN1	0.01246267	2.603097	down	5216 chr17	-
NM_021080	DAB1	0.0097914	2.6028035	down	1600 chr1	-
NM_213618	ST5	0.00171948	2.6020079	down	6764 chr11	-
NM_001042749	STAG2	0.03076377	2.6009235	down	10735 chrX	+
NM_032302	C7orf48	0.01836077	2.600028	down	84262 chr7	-
NM_015093	MAP3K7IP2	0.02123727	2.5992882	down	23118 chr6	+
NM_001037174	ARL5A	0.01582228	2.5986457	down	26225 chr2	-
NM_203462	MRFAP1L1	0.01858527	2.596454	down	114932 chr4	-
NM_018303	EXOC2	0.00243283	2.592241	down	55770 chr6	-
NM_005242	F2RL1	0.01809633	2.5920646	down	2150 chr5	+
NM_020786	PDP2	0.02926396	2.5873303	down	57546 chr16	+
NM_013386	SLC25A24	0.01068584	2.584878	down	29957 chr1	-
NM_013374	PDCD6IP	0.004333	2.5835576	down	10015 chr3	+
NM_015107	PHF8	0.00357691	2.5835447	down	23133 chrX	-
NM_015439	CCDC28A	8.11E-04	2.5824263	down	25901 chr6	+
NM_182907	PRDM1	0.03236028	2.5796402	down	639 chr6	+
NM_139013	MAPK14	9.56E-05	2.5790017	down	1432 chr6	+
NM_020439	CAMK1G	6.81E-05	2.577334	down	57172 chr1	+
NM_004612	TGFBR1	0.00805848	2.575956	down	7046 chr9	+
NM_019035	PCDH18	0.00223536	2.5759034	down	54510 chr4	-
NM_001122634	CPS1	0.0038327	2.575317	down	1373 chr2	+
NM_001421	ELF4	0.03004374	2.57515	down	2000 chrX	-
NM_005418	ST5	0.00110304	2.5737135	down	6764 chr11	-
NM_014344	FJX1	0.00553369	2.5726182	down	24147 chr11	+
NM_015441	OLFML2B	0.00194658	2.569306	down	25903 chr1	-
NM_004354	CCNG2	0.00534953	2.56855	down	901 chr4	+

NM_001035254	FAM102A	6.47E-04	2.5679984	down	399665	chr9	-
NM_001938	DR1	0.04788958	2.566601	down	1810	chr1	+
NM_007034	DNAJB4	0.00318108	2.5661132	down	11080	chr1	+
NM_003887	DDEF2	0.00355364	2.5644438	down	8853	chr2	+
NM_004468	FHL3	0.01480978	2.5635557	down	2275	chr1	-
NM_001082577	RBM9	0.00643119	2.5628939	down	23543	chr22	-
NM_001143688	DIS3L	0.00748489	2.561131	down	115752	chr15	+
NM_001003819	TRIM6-TRIM:	0.01334977	2.5581243	down	445372	chr11	+
NM_014324	AMACR	0.0280609	2.5575628	down	23600	chr5	-
NM_015508	TIPARP	0.01553939	2.5570414	down	25976	chr3	+
NM_006734	HIVEP2	0.03286791	2.5570152	down	3097	chr6	-
NM_173490	TMEM171	0.00353444	2.5561135	down	134285	chr5	+
NM_018180	DHX32	0.002333	2.555317	down	55760	chr10	-
NM_004733	SLC33A1	0.00226459	2.552945	down	9197	chr3	-
NM_030666	SERPINB1	0.04659597	2.5514686	down	1992	chr6	-
NM_207584	IFNAR2	0.03095403	2.5506456	down	3455	chr21	+
NM_018847	KLHL9	3.20E-04	2.550279	down	55958	chr9	-
NM_003687	PDLIM4	0.03841877	2.5497842	down	8572	chr5	+
NM_006956	ZNF12	0.01571091	2.549132	down	7559	chr7	-
NM_001018074	NR3C1	0.04991019	2.54879	down	2908	chr5	-
NM_000077	CDKN2A	3.91E-04	2.548181	down	1029	chr9	-
NM_006506	RASA2	3.39E-04	2.5478685	down	5922	chr3	+
NM_052861	MGC21675	0.0044767	2.5477943	down	92070	chr4	+
NM_004614	TK2	0.04755069	2.5468388	down	7084	chr16	-
NM_014909	VASH1	0.04788504	2.5463204	down	22846	chr14	+
NM_172114	CAMK2D	0.00349483	2.5461597	down	817	chr4	-
NM_144607	CYB5D1	0.0458821	2.5441475	down	124637	chr17	+
NM_052940	LRRC42	1.74E-04	2.5437539	down	115353	chr1	+
NM_006978	RNF113A	0.00988007	2.5433571	down	7737	chrX	-
NM_007066	PKIG	0.00467424	2.5429688	down	11142	chr20	+
NM_198273	LYSMD3	0.03091782	2.5426452	down	116068	chr5	-
NM_030799	YIPF5	0.00462285	2.541305	down	81555	chr5	-
NM_001236	CBR3	7.54E-04	2.5405443	down	874	chr21	+
NM_024520	C2orf47	0.0314212	2.5404906	down	79568	chr2	+
NM_144576	COQ10A	0.00314814	2.5394108	down	93058	chr12	+
NM_004580	RAB27A	0.00590371	2.539139	down	5873	chr15	-
NM_023016	ANKRD57	0.00321616	2.5377152	down	65124	chr2	+
NM_000710	BDKRB1	0.00167615	2.537535	down	623	chr14	+
NM_001142520	FAM111A	0.00522215	2.5359988	down	63901	chr11	+
NM_004232	SOCS6	0.00234916	2.5357106	down	9306	chr18	+
NM_024753	TTC21B	0.00750355	2.5319176	down	79809	chr2	-
NM_014892	RBM16	0.00801884	2.5308843	down	22828	chr6	+
NM_001099289	SH3RF3	0.00232557	2.5297344	down	344558	chr2	+
NM_002956	CLIP1	0.00378998	2.5284972	down	6249	chr12	-
NM_139006	HFE	0.04047745	2.5282874	down	3077	chr6	+
NM_001145354	MKLN1	0.02538241	2.527868	down	4289	chr7	+
NM_001105192	TLE3	0.00518787	2.527808	down	7090	chr15	-
NM_032436	C13orf8	0.02793037	2.5271597	down	283489	chr13	+
NM_152407	GRPEL2	0.00635666	2.5270686	down	134266	chr5	+

NM_006777	ZBTB33	0.00307734	2.5258677	down	10009	chrX	+
NM_000090	COL3A1	0.0064955	2.5240092	down	1281	chr2	+
NM_032581	FAM126A	0.02365384	2.5237064	down	84668	chr7	-
NM_006674	HCP5	0.03970558	2.522058	down	10866	chr6_cox_hap1	+
NM_014071	NCOA6	0.01937878	2.5208728	down	23054	chr20	-
NM_018098	ECT2	0.00290352	2.5162487	down	1894	chr3	+
NM_014213	HOXD9	0.01065647	2.5157032	down	3235	chr2	+
NM_144653	BTBD14A	0.03731737	2.5143557	down	138151	chr9	-
NM_005531	IFI16	0.00140738	2.5139136	down	3428	chr1	+
NM_017613	DONSON	0.003843	2.5131044	down	29980	chr21	-
NM_182976	ZNF326	2.44E-04	2.5124907	down	284695	chr1	+
NM_030971	SFXN3	0.00643618	2.5118024	down	81855	chr10	+
NM_007079	PTP4A3	0.00285885	2.5117974	down	11156	chr8	+
NM_025205	MED28	0.02218626	2.5099392	down	80306	chr4	+
NM_002519	NPAT	0.00287432	2.5090551	down	4863	chr11	-
NM_016307	PRRX2	0.00294356	2.5087235	down	51450	chr9	+
NM_004731	SLC16A7	1.57E-04	2.508609	down	9194	chr12	+
NM_013401	RAB3IL1	0.01121754	2.5077584	down	5866	chr11	-
NM_014363	SACS	9.75E-04	2.5060723	down	26278	chr13	-
NM_015317	PUM2	0.00128437	2.5053036	down	23369	chr2	-
NM_003872	NRP2	0.01183174	2.5043948	down	8828	chr2	+
NM_001031703	TMEM103	0.04085357	2.5037756	down	54859	chr3	-
NM_022745	ATPAF1	0.01199719	2.5027926	down	64756	chr1	-
NM_080671	KCNE4	4.34E-06	2.501424	down	23704	chr2	+
NM_007375	TARDBP	0.0223706	2.5001	down	23435	chr1	+
NM_001135000	FERMT2	2.75E-04	2.4995637	down	10979	chr14	-
NM_015534	ZZZ3	6.94E-05	2.4990408	down	26009	chr1	-
NM_016213	TRIP4	0.00799609	2.4982214	down	9325	chr15	+
NM_017860	C1orf56	0.01467125	2.4973226	down	54964	chr1	+
NM_002645	PIK3C2A	0.00984976	2.4970493	down	5286	chr11	-
NM_003908	EIF2S2	0.00392954	2.4917152	down	8894	chr20	-
NM_004235	KLF4	0.00867223	2.4866118	down	9314	chr9	-
NM_181519	SYT15	0.00366946	2.4859815	down	83849	chr10	-
NM_003496	TRRAP	0.00495021	2.4826667	down	8295	chr7	+
NM_203284	RBPJ	0.02282809	2.4811163	down	3516	chr4	+
NM_001235	SERPINH1	0.00626594	2.4806392	down	871	chr11	+
NM_001130483	VRK2	0.0123987	2.4801824	down	7444	chr2	+
NM_002742	PRKD1	0.01180398	2.479789	down	5587	chr14	-
NM_001005404	YPEL2	0.01214086	2.478913	down	388403	chr17	+
NM_001130914	BTG3	2.80E-04	2.4779513	down	10950	chr21	-
NM_005441	CHAF1B	0.0036169	2.4775648	down	8208	chr21	+
NM_144975	SLFN5	0.03053564	2.4769557	down	162394	chr17	+
NM_022748	TNS3	0.02496287	2.4767246	down	64759	chr7	-
NM_000321	RB1	1.46E-04	2.4763162	down	5925	chr13	+
NM_005081	ZNF142	0.04409391	2.4761355	down	7701	chr2	-
NM_015423	AASDHPPT	0.00949826	2.4752321	down	60496	chr11	+
NM_197974	BTN3A3	9.93E-04	2.4736185	down	10384	chr6	+
NM_019843	EIF4ENIF1	0.0043422	2.4733715	down	56478	chr22	-
NM_007247	AP1GBP1	0.01955102	2.4703667	down	11276	chr17	-

NM_001032372	ZNF226	0.02054164	2.4698505	down	7769	chr19	+
NM_183047	ZMYND8	0.01251537	2.4684675	down	23613	chr20	-
NM_144775	SMCR8	2.39E-04	2.4676476	down	140775	chr17	+
NM_001032290	PSRC1	0.00429314	2.467632	down	84722	chr1	-
NM_002913	RFC1	0.02075142	2.4668932	down	5981	chr4	-
NM_006575	MAP4K5	0.01459095	2.4657357	down	11183	chr14	-
NM_182969	XRRA1	0.00658149	2.4654326	down	143570	chr11	-
NM_020457	THAP11	0.00615634	2.4652581	down	57215	chr16	+
NM_198088	ZNF200	0.01755331	2.4647222	down	7752	chr16	-
NM_004290	RNF14	0.02726172	2.464195	down	9604	chr5	+
NM_015562	UBXN7	0.00197487	2.4637291	down	26043	chr3	-
NM_002267	KPNA3	0.03077244	2.463686	down	3839	chr13	-
NM_001006681	SPIN2B	0.01925287	2.462767	down	474343	chrX	-
NM_001037161	ACOT1	0.04823947	2.4627075	down	641371	chr14	+
NM_005871	SMNDC1	0.00227579	2.4624536	down	10285	chr10	-
NM_005171	ATF1	0.00264355	2.4622223	down	466	chr12	+
NM_001134438	PHLDB2	0.0124479	2.4620004	down	90102	chr3	+
NM_001004720	NCK2	0.03003534	2.4612427	down	8440	chr2	+
NM_001002265	MARCH8	0.0020173	2.4585915	down	220972	chr10	-
NM_152600	ZNF579	5.44E-04	2.458386	down	163033	chr19	-
NM_152316	C11orf46	0.00113216	2.458334	down	120534	chr11	+
NM_016067	MRPS18C	0.00598179	2.457415	down	51023	chr4	+
NM_005935	AFF1	0.01036815	2.4544337	down	4299	chr4	+
NM_021996	GBGT1	0.01036785	2.4540637	down	26301	chr9	-
NM_018023	YEATS2	0.00336445	2.453521	down	55689	chr3	+
NM_178170	NEK8	0.009066	2.4510076	down	284086	chr17	+
NM_181552	CUTL1	0.00292776	2.4503136	down	1523	chr7	+
NM_003503	CDC7	0.00840241	2.449528	down	8317	chr1	+
NM_153005	RIOK1	0.00848955	2.4482453	down	83732	chr6	+
NM_178313	SPTBN1	0.00248364	2.4462957	down	6711	chr2	+
NM_003368	USP1	0.0079379	2.4461462	down	7398	chr1	+
NM_002582	PARN	0.00175599	2.445544	down	5073	chr16	-
NM_032016	STARD3NL	0.00513747	2.4449823	down	83930	chr7	+
NM_053023	ZFP91	0.00147059	2.4431882	down	80829	chr11	+
NM_013411	AK2	9.70E-04	2.4410014	down	204	chr1	-
NM_001620	AHNAK	0.02034149	2.4398391	down	79026	chr11	-
NM_016042	EXOSC3	0.00142958	2.4389088	down	51010	chr9	-
NM_003026	SH3GL2	0.01878634	2.437019	down	6456	chr9	+
NM_017918	CCDC109B	0.01419841	2.4349139	down	55013	chr4	+
NM_005779	LHFPL2	0.0015844	2.4340754	down	10184	chr5	-
NM_024815	NUDT18	0.00706276	2.4330337	down	79873	chr8	-
NM_003995	NPR2	0.00753879	2.4330242	down	4882	chr9	+
NM_018121	C10orf6	0.00316928	2.4327161	down	55719	chr10	+
NM_002957	RXRA	0.0386654	2.431737	down	6256	chr9	+
NM_181659	NCOA3	0.00397307	2.431143	down	8202	chr20	+
NM_017719	SNRK	0.02758154	2.430286	down	54861	chr3	+
NM_001145001	NEK6	0.00706027	2.424365	down	10783	chr9	+
NM_016513	ICK	0.01398937	2.4241502	down	22858	chr6	-
NM_001143821	PLEKHA5	0.00459383	2.4230816	down	54477	chr12	+

NM_007214	SEC63	9.16E-04	2.420169	down	11231	chr6	-
NM_004331	BNIP3L	0.01052594	2.4191775	down	665	chr8	+
NM_024573	C6orf211	0.0454773	2.4183252	down	79624	chr6	+
NM_181805	PKIG	0.01673655	2.4181645	down	11142	chr20	+
NM_001017371	SP3	0.00280255	2.417331	down	6670	chr2	-
NM_004817	TJP2	0.00824893	2.4171538	down	9414	chr9	+
NM_004329	BMPR1A	0.01074665	2.4167998	down	657	chr10	+
NM_024691	ZNF419	0.01862013	2.4163685	down	79744	chr19	+
NM_203418	RCAN1	0.00413369	2.4156673	down	1827	chr21	-
NM_015322	FEM1B	0.00253815	2.4147398	down	10116	chr15	+
NM_152411	ZNF786	0.03765298	2.4137576	down	136051	chr7	-
NM_201999	ELF2	0.02921862	2.41331	down	1998	chr4	-
NM_052854	CREB3L1	0.00105524	2.4130085	down	90993	chr11	+
NM_001024847	TGFBR2	0.00461307	2.4127197	down	7048	chr3	+
NM_003198	TCEB3	0.04340597	2.412357	down	6924	chr1	+
NM_004985	KRAS	0.00634565	2.4122112	down	3845	chr12	-
NM_014055	IFT81	0.00593801	2.4120517	down	28981	chr12	+
NM_003359	UGDH	0.01049664	2.411768	down	7358	chr4	-
NM_152910	DGKH	0.00637464	2.4103565	down	160851	chr13	+
NM_175875	SIX5	0.00702697	2.409674	down	147912	chr19	-
NM_138394	HNRPLL	0.00209325	2.4088187	down	92906	chr2	-
NM_006994	BTN3A3	0.00388716	2.4067385	down	10384	chr6	+
NM_001130145	YAP1	0.00289828	2.4060464	down	10413	chr11	+
NM_001080512	BICC1	7.81E-06	2.4059367	down	80114	chr10	+
NM_080685	PTPN13	0.00678292	2.4052975	down	5783	chr4	+
NM_153271	SH3PX3	0.00113925	2.4045663	down	257364	chr15	+
NM_001128921	MARK3	0.01632553	2.4023657	down	4140	chr14	+
NM_015306	USP24	0.04398708	2.4022658	down	23358	chr1	-
NM_201283	EGFR	0.00240969	2.4020195	down	1956	chr7	+
NM_001128429	SMARCAD1	0.00968327	2.4005527	down	56916	chr4	+
NM_001098798	TOX2	0.01637975	2.3990324	down	84969	chr20	+
NM_001386	DPYSL2	0.03062122	2.398142	down	1808	chr8	+
NM_053053	TADA1L	0.01955052	2.3963933	down	117143	chr1	-
NM_001098797	TOX2	0.02175101	2.3956347	down	84969	chr20	+
NM_001706	BCL6	0.04375453	2.3939621	down	604	chr3	-
NM_004992	MECP2	1.88E-04	2.3934155	down	4204	chrX	-
NM_015578	LSM14A	0.02062157	2.3925178	down	26065	chr19	+
NM_022470	ZMAT3	0.00942496	2.391637	down	64393	chr3	-
NM_024057	NUP37	5.42E-04	2.390842	down	79023	chr12	-
NM_025135	FHOD3	0.02243176	2.390634	down	80206	chr18	+
NM_014570	ARFGAP3	0.01375651	2.3904185	down	26286	chr22	-
NM_006258	PRKG1	0.01180743	2.3902092	down	5592	chr10	+
NM_152231	FBXO34	8.05E-04	2.386763	down	55030	chr14	+
NM_022774	C1orf176	0.00576916	2.386506	down	64789	chr1	+
NM_005646	TARBP1	0.00830072	2.38567	down	6894	chr1	-
NM_021736	CLCN6	0.03924422	2.3844244	down	1185	chr1	+
NM_001127222	CACNA1A	0.01562144	2.382871	down	773	chr19	-
NM_201267	NRP2	0.01555446	2.3826015	down	8828	chr2	+
NM_020791	TAOK1	0.00289753	2.3822489	down	57551	chr17	+

NM_006259	PRKG2	0.00488922	2.3816237 down	5593	chr4	-
NM_014299	BRD4	0.02894887	2.3780777 down	23476	chr19	-
NM_014679	CEP57	6.91E-04	2.376632 down	9702	chr11	+
NM_015276	USP22	1.48E-04	2.3753629 down	23326	chr17	-
NM_015345	DAAM2	0.00533703	2.374596 down	23500	chr6	+
NM_001459	FLT3LG	0.0237124	2.3740044 down	2323	chr19	+
NM_000725	CACNB3	0.02482096	2.3728237 down	784	chr12	+
NM_032560	SMEK1	0.04622026	2.371686 down	55671	chr14	-
NM_015927	TGFB1I1	0.00288583	2.3712354 down	7041	chr16	+
NM_005444	RQCD1	0.01330801	2.3709016 down	9125	chr2	+
NM_138371	FAM113B	0.0190782	2.3686059 down	91523	chr12	+
NM_016248	AKAP11	0.0431833	2.3673153 down	11215	chr13	+
NM_006164	NFE2L2	0.028083	2.366805 down	4780	chr2	-
NM_020863	ZFAT1	0.00783797	2.366008 down	57623	chr8	-
NM_018137	PRMT6	0.01704108	2.3658345 down	55170	chr1	+
NM_007187	WBP4	6.21E-05	2.3651528 down	11193	chr13	+
NM_005400	PRKCE	0.04878024	2.3638585 down	5581	chr2	+
NM_178229	IQGAP3	0.00121843	2.3635375 down	128239	chr1	-
NM_006243	PPP2R5A	0.0022024	2.36275 down	5525	chr1	+
NM_005250	FOXL1	0.02311565	2.3615477 down	2300	chr16	+
NM_001143937	PSMA1	0.01228909	2.360947 down	5682	chr11	-
NM_016396	CTDSPL2	0.00269667	2.360332 down	51496	chr15	+
NM_002092	GRSF1	0.01364009	2.3602448 down	2926	chr4	-
NM_152415	VPS37A	0.01665621	2.3601675 down	137492	chr8	+
NM_145805	ISL2	0.043688	2.3590086 down	64843	chr15	+
NM_030817	APOLD1	0.03495482	2.3589065 down	81575	chr12	+
NM_144698	ANKRD35	0.01832108	2.3584387 down	148741	chr1	+
NM_152380	TBX15	5.76E-04	2.3577886 down	6913	chr1	-
NM_145036	CCDC46	0.0017424	2.3576133 down	201134	chr17	-
NM_001135937	SMAD2	0.01998612	2.356975 down	4087	chr18	-
NM_015506	MMACHC	0.01127109	2.356902 down	25974	chr1	+
NM_001098790	MID1IP1	0.00110616	2.3562524 down	58526	chrX	+
NM_002906	RDX	0.00629514	2.3561652 down	5962	chr11	-
NM_017875	SLC25A38	7.31E-04	2.35363 down	54977	chr3	+
NM_005982	SIX1	0.0183058	2.3518982 down	6495	chr14	-
NM_020899	ZBTB4	0.0186404	2.3518112 down	57659	chr17	-
NM_002718	PPP2R3A	0.04052142	2.349865 down	5523	chr3	+
NM_031915	SETDB2	6.23E-04	2.3494642 down	83852	chr13	+
NM_003879	CFLAR	0.00177556	2.3483517 down	8837	chr2	+
NM_152715	TBCEL	0.00174976	2.3480465 down	219899	chr11	+
NM_003010	MAP2K4	5.44E-04	2.3457358 down	6416	chr17	+
NM_003107	SOX4	0.03293786	2.3455987 down	6659	chr6	+
NM_173512	FLJ39822	3.05E-04	2.3444538 down	151258	chr2	-
NM_153713	LIX1L	0.03774288	2.3441098 down	128077	chr1	+
NM_033025	SYDE1	0.00162475	2.3438604 down	85360	chr19	+
NM_001130158	MYO1B	0.03004053	2.342649 down	4430	chr2	+
NM_138704	NDNL2	0.01679131	2.342433 down	56160	chr15	-
NM_003174	SVIL	0.04022186	2.3417666 down	6840	chr10	-
NM_198526	ZNF710	2.08E-04	2.3416457 down	374655	chr15	+

NM_001105209	LAMA4	0.02773753	2.3409772	down	3910	chr6	-
NM_000875	IGF1R	2.56E-04	2.3405578	down	3480	chr15	+
NM_058179	PSAT1	0.03266694	2.3389401	down	29968	chr9	+
NM_012460	TIMM9	2.32E-04	2.3385148	down	26520	chr14	-
NM_024036	LRFN4	0.00185282	2.3362143	down	78999	chr11	+
NM_199190	LARP4	6.29E-05	2.3338518	down	113251	chr12	+
NM_001017423	ALDH18A1	0.024801	2.3336682	down	5832	chr10	-
NM_032260	RGPD5	0.0249669	2.3331122	down	84220	chr2	-
NM_005701	SNUPN	0.00204546	2.3329103	down	10073	chr15	-
NM_182551	LYCAT	0.02437336	2.3327582	down	253558	chr2	+
NM_022497	MRPS25	0.02179105	2.3325648	down	64432	chr3	-
NM_173091	NFATC2	0.01639103	2.331639	down	4773	chr20	-
NM_001099858	C7orf25	0.02756349	2.330582	down	79020	chr7	-
NM_153333	TCEAL8	0.01249998	2.3300052	down	90843	chrX	-
NM_021252	RAB18	0.04336001	2.328992	down	22931	chr10	+
NM_001031725	DDX59	3.12E-04	2.3276775	down	83479	chr1	-
NM_032862	TIGD5	0.00718448	2.324689	down	84948	chr8	+
NM_001042459	FILIP1L	0.0100822	2.3226974	down	11259	chr3	-
NM_030801	MAGED4	9.24E-04	2.322458	down	81557	chrX	+
NM_002657	PLAGL2	0.01218906	2.322243	down	5326	chr20	-
NM_144600	C16orf63	0.03715458	2.3218162	down	123811	chr16	-
NM_001025247	TAF5L	0.01556825	2.3214273	down	27097	chr1	-
NM_020772	NUFIP2	0.0085501	2.3201716	down	57532	chr17	-
NM_001116	ADCY9	0.03783839	2.3180804	down	115	chr16	-
NM_173515	CNKSR3	0.01942761	2.317925	down	154043	chr6	-
NM_001033561	PHF12	1.46E-04	2.3153844	down	57649	chr17	-
NM_012068	ATF5	5.10E-04	2.3148022	down	22809	chr19	+
NM_006301	MAP3K12	0.01260326	2.3123703	down	7786	chr12	-
NM_003450	ZNF174	0.00983072	2.3120837	down	7727	chr16	+
NM_172210	CSF1	0.00306337	2.311646	down	1435	chr1	+
NM_002830	PTPN4	0.02338705	2.3113916	down	5775	chr2	+
NM_016205	PDGFC	0.02012678	2.3104193	down	56034	chr4	-
NM_153770	CABYR	0.01335594	2.3102055	down	26256	chr18	+
NM_013286	RBM15B	0.01781711	2.309561	down	29890	chr3	+
NM_003276	TMPO	0.0322003	2.3092399	down	7112	chr12	+
NM_019096	GTPBP2	0.01062875	2.3087978	down	54676	chr6	-
NM_020177	FEM1C	0.04996236	2.3067415	down	56929	chr5	-
NM_032373	PCGF5	0.01618874	2.3043997	down	84333	chr10	+
NM_022740	HIPK2	0.00543505	2.299269	down	28996	chr7	-
NM_182811	PLCG1	0.00606004	2.2971566	down	5335	chr20	+
NM_006372	SYNCRIP	0.00421677	2.296742	down	10492	chr6	-
NM_015308	FNBP4	0.00591651	2.2967246	down	23360	chr11	-
NM_022074	FAM111A	0.02561124	2.2951498	down	63901	chr11	+
NM_018200	HMG20A	0.00558121	2.2945604	down	10363	chr15	+
NM_175085	GART	0.0337188	2.2934687	down	2618	chr21	-
NM_033254	BOC	0.00119327	2.2934225	down	91653	chr3	+
NM_005044	PRKX	0.00217726	2.2924743	down	5613	chrX	-
NM_032145	FBXO30	0.03650091	2.2886422	down	84085	chr6	-
NM_005744	ARIH1	0.02961285	2.285548	down	25820	chr15	+

NM_017771	PXK	0.00189293	2.2824576	down	54899	chr3	+
NM_000408	GPD2	0.0221734	2.2818797	down	2820	chr2	+
NM_001012979	TCEAL5	0.00316945	2.2818162	down	340543	chrX	-
NM_015045	WAPAL	0.02731607	2.28098	down	23063	chr10	-
NM_001048265	C9orf116	0.01157084	2.2793944	down	138162	chr9	-
NM_005032	PLS3	0.0481642	2.2789147	down	5358	chrX	+
NM_199290	NACA2	0.0126924	2.2779026	down	342538	chr17	-
NM_001145306	CDK6	0.0078043	2.2766018	down	1021	chr7	-
NM_001039650	ZMYM5	0.00259047	2.2763236	down	9205	chr13	-
NM_001007101	ZNF484	0.00112166	2.2762911	down	83744	chr9	-
NM_033389	SSH2	0.0109634	2.2762754	down	85464	chr17	-
NM_003797	EED	0.01479803	2.2761917	down	8726	chr11	+
NM_003246	THBS1	1.98E-04	2.272655	down	7057	chr15	+
NM_000416	IFNGR1	0.02272826	2.2712042	down	3459	chr6	-
NM_012110	CHIC2	0.00994339	2.270401	down	26511	chr4	-
NM_032728	PPAPDC3	0.01059708	2.268993	down	84814	chr9	+
NM_020437	ASPHD2	0.04855453	2.266216	down	57168	chr22	+
NM_001033059	AMD1	0.02015499	2.2630036	down	262	chr6	+
NM_001114636	FANCL	0.00210039	2.2613018	down	55120	chr2	-
NM_194313	KIF24	0.02675938	2.2612593	down	347240	chr9	-
NM_012096	APPL1	0.00849825	2.2565534	down	26060	chr3	+
NM_032231	FAM96A	0.0010913	2.253645	down	84191	chr15	-
NM_001065	TNFRSF1A	4.04E-05	2.253122	down	7132	chr12	-
NM_032849	C13orf33	0.03410089	2.2530813	down	84935	chr13	+
NM_001015891	TAF9	0.00515851	2.251937	down	6880	chr5	-
NM_001145031	PLAU	4.40E-04	2.251684	down	5328	chr10	+
NM_001122607	RUNX1	0.02352668	2.2488353	down	861	chr21	-
NM_016080	GLOD4	0.00435694	2.2484102	down	51031	chr17	-
NM_017798	YTHDF1	0.01500113	2.2453299	down	54915	chr20	-
NM_177414	PPAP2B	0.01807712	2.2442229	down	8613	chr1	-
NM_182485	CPEB2	6.83E-04	2.2440655	down	132864	chr4	+
NM_014089	NUPL1	0.04784878	2.2431343	down	9818	chr13	+
NM_206909	PSD3	0.02843227	2.2430384	down	23362	chr8	-
NM_001007075	KLHL5	0.00889132	2.2420208	down	51088	chr4	+
NM_024605	ARHGAP10	7.87E-04	2.2416592	down	79658	chr4	+
NM_030786	SYNC1	0.00499218	2.2408621	down	81493	chr1	-
NM_001136540	APOL1	0.00712928	2.239183	down	8542	chr22	+
NM_030775	WNT5B	0.04091916	2.236776	down	81029	chr12	+
NM_003109	SP1	0.03160788	2.2367005	down	6667	chr12	+
NM_022652	DUSP6	0.02037264	2.2352743	down	1848	chr12	-
NM_001789	CDC25A	0.00309822	2.2350314	down	993	chr3	-
NM_006931	SLC2A3	7.27E-04	2.234809	down	6515	chr12	-
NM_018232	FAM21B	0.04388657	2.234165	down	55747	chr10	+
NM_001099270	ZBTB34	0.01130162	2.231767	down	403341	chr9	+
NM_000636	SOD2	0.00571472	2.2290049	down	6648	chr6	-
NM_016002	SCCPDH	0.0378165	2.2270534	down	51097	chr1	+
NM_001037872	REV1	0.01492767	2.2247095	down	51455	chr2	-
NM_024095	ASB8	0.02165519	2.2240205	down	140461	chr12	-
NM_001802	CDR2	0.00407495	2.222747	down	1039	chr16	-

NM_006598	SLC12A7	0.04216797	2.2193775	down	10723	chr5	-
NM_181861	APAF1	0.03953788	2.2184665	down	317	chr12	+
NM_019593	RP5-1022P6.2	0.00398713	2.2175627	down	56261	chr20	-
NM_024685	BBS10	0.04811101	2.2169082	down	79738	chr12	-
NM_001025356	TMEM16F	0.02534123	2.2154155	down	196527	chr12	+
NM_001007246	BRWD1	0.04793275	2.2149177	down	54014	chr21	-
NM_006807	CBX1	0.01476815	2.2146277	down	10951	chr17	-
NM_001039091	PRPS2	0.02251317	2.214354	down	5634	chrX	+
NM_001068	TOP2B	0.02114281	2.213018	down	7155	chr3	-
NM_080632	UPF3B	0.00813392	2.2128592	down	65109	chrX	-
NM_198833	SERPINB8	0.01001297	2.2124782	down	5271	chr18	+
NM_015995	KLF13	0.01225838	2.2115088	down	51621	chr15	+
NM_031157	HNRPA1	0.01360525	2.2112296	down	3178	chr12	+
NM_181302	WDR20	0.03504906	2.2089906	down	91833	chr14	+
NM_206826	GNL3	0.02194847	2.2085593	down	26354	chr3	+
NM_001114121	CHEK1	0.00423291	2.2080994	down	1111	chr11	+
NM_001098512	PRKG1	3.23E-04	2.2075958	down	5592	chr10	+
NM_004661	CDC23	0.03304815	2.2072778	down	8697	chr5	-
NM_003821	RIPK2	0.03987789	2.2071822	down	8767	chr8	+
NM_002731	PRKACB	0.01129723	2.2071757	down	5567	chr1	+
NM_002285	AFF3	0.01342876	2.2067292	down	3899	chr2	-
NM_016463	CXXC5	0.00455747	2.206312	down	51523	chr5	+
NM_024911	GPR177	0.03143137	2.20621	down	79971	chr1	-
NM_005402	RALA	0.00118308	2.2050188	down	5898	chr7	+
NM_018355	ZNF415	0.02156345	2.2047288	down	55786	chr19	-
NM_006999	POLS	0.0130648	2.2033882	down	11044	chr5	+
NM_003076	SMARCD1	0.02752818	2.2024155	down	6602	chr12	+
NM_018427	RRN3	0.01419502	2.2022169	down	54700	chr16	-
NM_199357	ARHGAP11A	0.04199719	2.201546	down	9824	chr15	+
NM_001001414	NCCRP1	0.04883329	2.2011013	down	342897	chr19	+
NM_017622	C17orf59	0.00327203	2.1981983	down	54785	chr17	-
NM_014774	KIAA0494	7.55E-04	2.1981823	down	9813	chr1	-
NM_032876	JUB	0.02010996	2.1979403	down	84962	chr14	-
NM_017730	QRICH1	0.01657326	2.196642	down	54870	chr3	-
NM_001128833	ZBTB4	0.02643222	2.196273	down	57659	chr17	-
NM_004914	RAB36	0.01088503	2.1955407	down	9609	chr22	+
NM_017437	CPSF2	0.02475247	2.192901	down	53981	chr14	+
NM_172129	CAMK2D	0.00171647	2.1926408	down	817	chr4	-
NM_001145464	EXOG	0.0052286	2.1917083	down	9941	chr3	+
NM_001605	AARS	0.01293176	2.1903446	down	16	chr16	-
NM_006311	NCOR1	0.04282363	2.189968	down	9611	chr17	-
NM_006973	ZNF32	0.01502588	2.1896808	down	7580	chr10	-
NM_012430	SEC22A	0.03413583	2.188864	down	26984	chr3	+
NM_005016	PCBP2	0.00899275	2.1878529	down	5094	chr12	+
NM_194441	BTN3A1	0.03938532	2.1865993	down	11119	chr6	+
NM_005156	ROD1	0.00794523	2.1864612	down	9991	chr9	-
NM_014830	ZBTB39	0.03307746	2.1862175	down	9880	chr12	-
NM_022754	SFXN1	0.01859687	2.1848783	down	94081	chr5	+
NM_000899	KITLG	0.0248405	2.1821332	down	4254	chr12	-

NM_016138	COQ7	0.01149953	2.1818662	down	10229	chr16	+
NM_203327	SLC23A2	0.04607092	2.181722	down	9962	chr20	-
NM_014757	MAML1	0.00669576	2.1810882	down	9794	chr5	+
NM_004688	NMI	0.00304565	2.180883	down	9111	chr2	-
NM_003473	STAM	0.03263955	2.1804338	down	8027	chr10	+
NM_015130	TBC1D9	8.80E-05	2.1794806	down	23158	chr4	-
NM_021783	EDA2R	0.01893687	2.1792407	down	60401	chrX	-
NM_001134340	PSMG3	0.04227895	2.1786897	down	84262	chr7	-
NM_144628	TBC1D20	0.02191909	2.177997	down	128637	chr20	-
NM_006494	ERF	0.00684401	2.1777194	down	2077	chr19	-
NM_032424	KIAA1826	0.00883046	2.176601	down	84437	chr11	-
NM_033624	FBXO21	0.01081204	2.1765606	down	23014	chr12	-
NM_017811	UBE2R2	0.0045994	2.1755085	down	54926	chr9	+
NM_001080425	BEXL1	0.00570376	2.172135	down	56271	chrX	+
NM_001130829	CDC2	0.00259569	2.171014	down	983	chr10	+
NM_001813	CENPE	5.86E-04	2.1683564	down	1062	chr4	-
NM_000057	BLM	0.02668149	2.1682117	down	641	chr15	+
NM_022648	TNS1	7.04E-04	2.1649206	down	7145	chr2	-
NM_012121	CDC42EP4	0.00542936	2.163869	down	23580	chr17	-
NM_152288	TMEM142C	0.00273816	2.1610672	down	93129	chr16	+
NM_145060	C18orf24	0.02712835	2.1605659	down	220134	chr18	+
NM_014600	EHD3	0.01619548	2.1592994	down	30845	chr2	+
NM_001033886	CXCL12	0.0250373	2.1591623	down	6387	chr10	-
NM_016333	SRRM2	0.01435939	2.1587114	down	23524	chr16	+
NM_018668	VPS33B	0.00988505	2.15871	down	26276	chr15	-
NM_021644	HNRNPH3	0.00104408	2.1586394	down	3189	chr10	+
NM_005067	SIAH2	3.83E-04	2.1576128	down	6478	chr3	-
NM_003584	DUSP11	0.00743061	2.1559162	down	8446	chr2	-
NM_018453	EAPP	0.00403983	2.1557338	down	55837	chr14	-
NM_004206	SEC22C	0.00433514	2.1544476	down	9117	chr3	-
NM_020931	KIAA1586	0.01419422	2.153788	down	57691	chr6	+
NM_138799	MBOAT2	0.00397979	2.1511126	down	129642	chr2	-
NM_015360	SKIV2L2	0.01568279	2.1503165	down	23517	chr5	+
NM_015455	CNOT6	0.02077194	2.1488023	down	57472	chr5	+
NM_021242	MID1IP1	2.48E-04	2.1470928	down	58526	chrX	+
NM_001417	EIF4B	0.01810893	2.14684	down	1975	chr12	+
NM_006277	ITSN2	0.01309806	2.146184	down	50618	chr2	-
NM_174916	UBR1	2.95E-04	2.1457648	down	197131	chr15	-
NM_004792	PPIG	0.00803048	2.1451492	down	9360	chr2	+
NM_016340	RAPGEF6	0.04690317	2.1429632	down	51735	chr5	-
NM_022039	FBXW4	0.00761771	2.1424708	down	6468	chr10	-
NM_052860	ZNF300	0.02437108	2.1418288	down	91975	chr5	-
NM_173825	RABL3	0.00681682	2.1411562	down	285282	chr3	-
NM_006101	NDC80	0.04156726	2.140114	down	10403	chr18	+
NM_004776	B4GALT5	0.03448589	2.139018	down	9334	chr20	-
NM_006264	PTPN13	0.03382872	2.1382716	down	5783	chr4	+
NM_004799	ZFYVE9	0.04959225	2.13807	down	9372	chr1	+
NM_017527	LY6K	0.02559366	2.1380632	down	54742	chr8	+
NM_005873	RGS19	0.00380013	2.1374915	down	10287	chr20	-

NM_138957	MAPK1	3.34E-04	2.1370027	down	5594	chr22	-
NM_014810	CEP350	0.01081364	2.1369326	down	9857	chr1	+
NM_017749	FLJ20294	0.00393584	2.1368473	down	55626	chr11	-
NM_058219	EXOSC6	0.00418341	2.1359632	down	118460	chr16	-
NM_024561	NARG1L	0.03645242	2.134743	down	79612	chr13	+
NM_001145638	GPSM1	0.02462605	2.1329112	down	26086	chr9	+
NM_021926	ALX4	0.01657125	2.1323621	down	60529	chr11	-
NM_000179	MSH6	0.0080923	2.13225	down	2956	chr2	+
NM_001010983	GLT8D1	0.00569343	2.1301017	down	55830	chr3	-
NM_001143779	IFT81	0.00647448	2.1295457	down	28981	chr12	+
NM_057167	COL6A3	0.01370822	2.129492	down	1293	chr2	-
NM_022492	TTC31	7.33E-04	2.1289573	down	64427	chr2	+
NM_173811	C11orf77	0.00987169	2.1289234	down	283254	chr11	-
NM_004009	DMD	0.00129862	2.1284711	down	1756	chrX	-
NM_001136039	NIF3L1	0.0199452	2.1282313	down	60491	chr2	+
NM_016448	DTL	7.39E-04	2.127272	down	51514	chr1	+
NM_006823	PKIA	0.02612715	2.1263294	down	5569	chr8	+
NM_000946	PRIM1	0.00252869	2.1243849	down	5557	chr12	-
NM_015382	HECTD1	0.02672214	2.1241455	down	25831	chr14	-
NM_001130031	ZNF562	0.00798285	2.123869	down	54811	chr19	-
NM_032773	LRCH3	0.01733072	2.123285	down	84859	chr3	+
NM_021908	ST7	0.00391015	2.1231172	down	7982	chr7	+
NM_005911	MAT2A	0.00160304	2.121287	down	4144	chr2	+
NM_031473	IFT81	0.00899867	2.1212633	down	28981	chr12	+
NM_018151	RIF1	0.02019609	2.1208098	down	55183	chr2	+
NM_001067	TOP2A	0.00462319	2.1202204	down	7153	chr17	-
NM_004215	EBAG9	0.03749155	2.1174471	down	9166	chr8	+
NM_032423	ZNF528	0.0354295	2.1173842	down	84436	chr19	+
NM_005030	PLK1	0.0023029	2.1173365	down	5347	chr16	+
NM_152243	CDC42EP1	0.00296115	2.1139786	down	11135	chr22	+
NM_017420	SIX4	5.10E-04	2.1136963	down	51804	chr14	-
NM_005077	TLE1	0.04468322	2.1136384	down	7088	chr9	-
NM_002760	PRKY	0.02328938	2.1134994	down	5616	chrY	+
NM_032926	TCEAL3	4.41E-04	2.1129282	down	85012	chrX	+
NM_017741	C4orf30	0.00380774	2.1128945	down	54876	chr4	-
NM_145658	SPESP1	0.01215637	2.111864	down	246777	chr15	+
NM_017761	PNRC2	0.00241126	2.1109917	down	55629	chr1	+
NM_000784	CYP27A1	0.00230798	2.1107697	down	1593	chr2	+
NM_001112704	VAX1	0.00173122	2.1086597	down	11023	chr10	-
NM_173546	KLHDC8B	0.02310354	2.1084952	down	200942	chr3	+
NM_003804	RIPK1	0.00586566	2.1079226	down	8737	chr6	+
NM_000859	HMGCR	0.02153323	2.1057472	down	3156	chr5	+
NM_003581	NCK2	0.00197612	2.1057153	down	8440	chr2	+
NM_021824	NIF3L1	0.00312015	2.1049802	down	60491	chr2	+
NM_001040444	PHF11	0.01584358	2.1046228	down	51131	chr13	+
NM_001145	ANG	0.00660669	2.1026974	down	283	chr14	+
NM_182488	USP12	0.01846943	2.101608	down	219333	chr13	-
NM_001033504	TMEM98	0.03999928	2.1009433	down	26022	chr17	+
NM_001033024	FBXO7	0.0044843	2.0991526	down	25793	chr22	+

NM_005973	PRCC	0.01156136	2.0981593	down	5546	chr1	+
NM_145267	C6orf57	0.00410503	2.0973246	down	135154	chr6	+
NM_004251	RAB9A	0.00446241	2.0959246	down	9367	chrX	+
NM_001127454	DFNA5	0.03365798	2.0956948	down	1687	chr7	-
NM_001101392	LOC645545	0.01079901	2.0950243	down	645545	chr17	-
NM_145251	STYX	0.00255611	2.0947418	down	6815	chr14	+
NM_006401	ANP32B	5.34E-05	2.094357	down	10541	chr9	+
NM_003875	GMPS	0.03563012	2.0931695	down	8833	chr3	+
NM_016587	CBX3	0.01692713	2.0922356	down	11335	chr7	+
NM_014345	ZNF318	0.00669227	2.090885	down	24149	chr6	-
NM_001145009	BTN3A1	0.02307651	2.088455	down	11119	chr6	+
NM_021144	PSIP1	0.0016858	2.0883307	down	11168	chr9	-
NM_001143979	NDE1	0.01858048	2.0871675	down	54820	chr16	+
NM_001008567	TSC1	0.03105689	2.0870192	down	7248	chr9	-
NM_000189	HK2	0.00738602	2.0858958	down	3099	chr2	+
NM_001142643	CASKIN2	0.03608215	2.0849555	down	57513	chr17	-
NM_001100426	RAP1GDS1	0.03133414	2.0838802	down	5910	chr4	+
NM_152515	CKAP2L	0.02415486	2.0830896	down	150468	chr2	-
NM_001077704	MIER1	0.00689428	2.082994	down	57708	chr1	+
NM_012453	TBL2	0.00313475	2.0829513	down	26608	chr7	-
NM_198268	HIPK1	0.00989573	2.0826945	down	204851	chr1	+
NM_001033	RRM1	0.01681196	2.08249	down	6240	chr11	+
NM_001077703	MIER1	0.0068794	2.0816214	down	57708	chr1	+
NM_018195	C11orf57	0.02035226	2.0815217	down	55216	chr11	+
NM_002908	REL	0.04585659	2.0806365	down	5966	chr2	+
NM_015459	DKFZP564J08	0.01083403	2.076767	down	25923	chr11	-
NM_014967	MTMR15	0.01095125	2.076181	down	22909	chr15	+
NM_001023560	ZNF187	0.00621522	2.0758276	down	7741	chr6	+
NM_003934	FUBP3	0.02038708	2.075488	down	8939	chr9	+
NM_001040023	SIRPA	6.19E-05	2.0748146	down	140885	chr20	+
NM_001102398	HNRNPR	0.04070677	2.074381	down	10236	chr1	-
NM_001015051	RUNX2	0.04877402	2.0718505	down	860	chr6	+
NM_001008699	IL4R	0.01151952	2.0712242	down	3566	chr16	+
NM_001129897	NUSAP1	0.00704303	2.071101	down	51203	chr15	+
NM_032828	ZNF587	0.00629637	2.06993	down	84914	chr19	+
NM_006390	IPO8	3.33E-04	2.0684357	down	10526	chr12	-
NM_182964	NAV2	0.02675766	2.0673585	down	89797	chr11	+
NM_001040443	PHF11	0.0054152	2.0670934	down	51131	chr13	+
NM_001099666	PTAR1	0.03322042	2.066801	down	375743	chr9	-
NM_145301	FAM18B2	0.01097511	2.0665271	down	201158	chr17	-
NM_003436	ZNF135	0.0219198	2.0661993	down	7694	chr19	+
NM_001007559	SS18	0.00682152	2.0655432	down	6760	chr18	-
NM_004723	ARHGEF2	0.00479644	2.06518	down	9181	chr1	-
NM_024018	BTN2A3	0.01031158	2.0645719	down	54718	chr6	+
NM_030918	SNX27	0.01681823	2.0645657	down	81609	chr1	+
NM_001541	HSPB2	0.01489805	2.0639558	down	3316	chr11	+
NM_003799	RNMT	0.02086327	2.0638154	down	8731	chr18	+
NM_003720	DSCR2	0.01394988	2.0632055	down	8624	chr21	-
NM_002428	MMP15	0.01933135	2.0624814	down	4324	chr16	+

NM_003342	UBE2G1	0.02121799	2.059227	down	7326	chr17	-
NM_052827	CDK2	0.02276572	2.0578036	down	1017	chr12	+
NM_001002258	ATP5G3	0.02354072	2.0553539	down	518	chr2	-
NM_002199	IRF2	0.04387054	2.0550978	down	3660	chr4	-
NM_024552	LASS4	0.00351162	2.054096	down	79603	chr19	+
NM_015449	C1orf43	0.01579087	2.0531702	down	25912	chr1	-
NM_152703	SAMD9L	0.00131107	2.0513816	down	219285	chr7	-
NM_012485	HMMR	0.00577726	2.05121	down	3161	chr5	+
NM_020946	DENND1A	0.03561471	2.0500712	down	57706	chr9	-
NM_182796	MAT2B	0.00406184	2.049356	down	27430	chr5	+
NM_015974	CRYL1	0.00157126	2.0484633	down	51084	chr13	-
NM_203406	LOC153364	0.02100905	2.048248	down	153364	chr5	-
NM_194330	RNF38	0.03535767	2.0472627	down	152006	chr9	-
NM_001083335	ZFP112	0.03278717	2.0471096	down	7771	chr19	-
NM_006936	SUMO3	0.00873707	2.0470326	down	6612	chr21	-
NM_020876	ARHGAP23	0.04124451	2.0465603	down	57636	chr17	+
NM_006473	TAF6L	0.02087801	2.0463243	down	10629	chr11	+
NM_001002000	GMPR2	0.00912326	2.044647	down	51292	chr14	+
NM_001142498	SIRT1	0.02628323	2.0446064	down	23411	chr10	+
NM_001128303	C13orf31	0.00635388	2.0444362	down	144811	chr13	+
NM_002113	CFHR1	0.02647628	2.0432134	down	3078	chr1	+
NM_006412	AGPAT2	0.00293319	2.0430987	down	10555	chr9	-
NM_016648	LARP7	0.00675176	2.043046	down	51574	chr4	+
NM_002497	NEK2	0.00671451	2.0417662	down	4751	chr1	-
NM_001099337	COQ10A	0.0126595	2.041242	down	93058	chr12	+
NM_017512	ENOSF1	0.01789838	2.0411313	down	55556	chr18	-
NM_014393	STAU2	2.18E-04	2.0411224	down	27067	chr8	-
NM_145271	ZNF688	0.01451892	2.041094	down	146542	chr16	-
NM_006357	UBE2E3	0.00194924	2.040479	down	10477	chr2	+
NM_012423	RPL13A	0.00219239	2.0403278	down	23521	chr19	+
NM_053279	C8orf13	0.03817987	2.0393975	down	83648	chr8	-
NM_153035	C1orf83	0.01979151	2.0388448	down	127428	chr1	+
NM_022373	HERPUD2	0.02884603	2.0374596	down	64224	chr7	-
NM_002993	CXCL6	0.01447649	2.0373816	down	6372	chr4	+
NM_014839	LPPR4	2.59E-04	2.0371413	down	9890	chr1	+
NM_018686	CMAS	0.0049334	2.036221	down	55907	chr12	+
NM_004701	CCNB2	2.63E-04	2.035275	down	9133	chr15	+
NM_001024629	NRP1	8.81E-04	2.0352602	down	8829	chr10	-
NM_014672	KIAA0391	0.003464	2.034004	down	9692	chr14	+
NM_201566	SLC16A13	0.00142749	2.0337498	down	201232	chr17	+
NM_030925	CAB39L	0.02650607	2.032938	down	81617	chr13	-
NM_013296	GPSM2	0.00230203	2.0328991	down	29899	chr1	+
NM_004239	TRIP11	0.00327635	2.0327728	down	9321	chr14	-
NM_001099679	TRIM32	0.00979057	2.0322306	down	22954	chr9	+
NM_006350	FST	0.0015173	2.0319908	down	10468	chr5	+
NM_015365	AMMECR1	0.02544943	2.0307755	down	9949	chrX	-
NM_152374	FLJ38984	0.00354741	2.0304508	down	127703	chr1	-
NM_006092	NOD1	0.02910663	2.0287757	down	10392	chr7	-
NM_020242	KIF15	5.72E-04	2.0282373	down	56992	chr3	+

NM_001799	CDK7	0.00219976	2.0281367	down	1022	chr5	+
NM_012291	ESPL1	0.00846236	2.0280852	down	9700	chr12	+
NM_024771	NAT11	0.01706212	2.0275805	down	79829	chr11	+
NM_181802	UBE2C	0.01243532	2.0271194	down	11065	chr20	+
NM_144658	DOCK11	0.04635722	2.026944	down	139818	chrX	+
NM_001142568	BBX	0.00755644	2.0229282	down	56987	chr3	+
NM_003692	TMEFF1	0.00495798	2.0228922	down	8577	chr9	+
NM_001412	EIF1AX	0.01287575	2.022477	down	1964	chrX	-
NM_184234	RBM39	0.01149351	2.02232	down	9584	chr20	-
NM_020710	LRRC47	0.02366046	2.0212667	down	57470	chr1	-
NM_021154	PSAT1	0.04293658	2.0211117	down	29968	chr9	+
NM_153181	NETO1	0.00227864	2.0208566	down	81832	chr18	-
NM_019005	FLJ20323	1.03E-04	2.0185122	down	54468	chr7	+
NM_015990	KLHL5	0.01506164	2.018156	down	51088	chr4	+
NM_001142422	MORF4L2	0.02359052	2.017768	down	9643	chrX	-
NM_001039661	TIRAP	0.00110546	2.0169842	down	114609	chr11	+
NM_000070	CAPN3	0.01094511	2.0169637	down	825	chr15	+
NM_001142497	MLKL	0.0349524	2.0165818	down	197259	chr16	-
NM_152716	FLJ36874	0.00808821	2.0158334	down	219988	chr11	-
NM_031491	RBP5	7.05E-04	2.0150566	down	83758	chr12	-
NM_178342	C3orf35	0.00168443	2.0145745	down	339883	chr3	+
NM_152624	DCP2	3.04E-04	2.0139453	down	167227	chr5	+
NM_001076786	QSER1	0.02203129	2.0135672	down	79832	chr11	+
NM_019606	BCDIN3	0.00481554	2.012095	down	56257	chr7	+
NM_012175	FBXO3	0.00275738	2.011256	down	26273	chr11	-
NM_001080443	KIF18B	0.04454244	2.0087314	down	146909	chr17	-
NM_152272	CHMP7	0.01889934	2.0085561	down	91782	chr8	+
NM_207009	FAM45A	0.03235519	2.0063694	down	404636	chr10	+
NM_001002010	NT5C3	0.02459374	2.0063016	down	51251	chr7	-
NM_015454	LARP7	0.00386197	2.0062644	down	51574	chr4	+
NM_153260	LRRC57	0.00232606	2.0048897	down	255252	chr15	-
NM_001005743	NUMB	0.01730024	2.004495	down	8650	chr14	-
NM_001130048	DOCK9	0.02401522	2.0043795	down	23348	chr13	-
NM_012228	MSRB2	0.04801792	2.003823	down	22921	chr10	+
NM_005429	VEGFC	0.00271024	2.0008185	down	7424	chr4	-
NM_007040	HNRPUL1	0.00605413	2.0001085	down	11100	chr19	+
